# Clinical outcomes of antimicrobial resistance in cancer patients: a systematic review of multivariable models

**DOI:** 10.1186/s12879-023-08182-3

**Published:** 2023-04-18

**Authors:** Anders Skyrud Danielsen, Léa Franconeri, Samantha Page, Anders Eivind Myhre, Ragnhild Agathe Tornes, Oliver Kacelnik, Jørgen Vildershøj Bjørnholt

**Affiliations:** 1 Department of Microbiology, Oslo University Hospital, Oslo, Norway; 2 Institute of Clinical Medicine, Faculty of Medicine, University of Oslo, Oslo, Norway; 3 Department of Infection Control and Preparedness, Norwegian Institute of Public Health, Oslo, Norway; 4 ECDC Fellowship Programme, Field Epidemiology Path (EPIET), European Centre for Disease Prevention and Control, (ECDC), Stockholm, Sweden; 5 Department of Haematology, Oslo University Hospital, Oslo, Norway; 6 The Library for the Healthcare Administration, Norwegian Institute of Public Health, Oslo, Norway

**Keywords:** Antimicrobial resistance, Infection, Mortality, Risk factors, Cancer, Malignancy, Systematic review

## Abstract

**Background:**

Infections are major causes of disease in cancer patients and pose a major obstacle to the success of cancer care. The global rise of antimicrobial resistance threatens to make these obstacles even greater and hinder continuing progress in cancer care. To prevent and handle such infections, better models of clinical outcomes building on current knowledge are needed. This internally funded systematic review (PROSPERO registration: CRD42021282769) aimed to review multivariable models of resistant infections/colonisations and corresponding mortality, what risk factors have been investigated, and with what methodological approaches.

**Methods:**

We employed two broad searches of antimicrobial resistance in cancer patients, using terms associated with antimicrobial resistance, in MEDLINE and Embase through Ovid, in addition to Cinahl through EBSCOhost and Web of Science Core Collection. Primary, observational studies in English from January 2015 to November 2021 on human cancer patients that explicitly modelled infection/colonisation or mortality associated with antimicrobial resistance in a multivariable model were included. We extracted data on the study populations and their malignancies, risk factors, microbial aetiology, and methods for variable selection, and assessed the risk of bias using the NHLBI Study Quality Assessment Tools.

**Results:**

Two searches yielded a total of 27,151 unique records, of which 144 studies were included after screening and reading. Of the outcomes studied, mortality was the most common (68/144, 47%). Forty-five per cent (65/144) of the studies focused on haemato-oncological patients, and 27% (39/144) studied several bacteria or fungi. Studies included a median of 200 patients and 46 events. One-hundred-and-three (72%) studies used a *p*-value-based variable selection. Studies included a median of seven variables in the final (and largest) model, which yielded a median of 7 events per variable. An in-depth example of vancomycin-resistant enterococci was reported.

**Conclusions:**

We found the current research to be heterogeneous in the approaches to studying this topic. Methodological choices resulting in very diverse models made it difficult or even impossible to draw statistical inferences and summarise what risk factors were of clinical relevance. The development and adherence to more standardised protocols that build on existing literature are urgent.

**Supplementary Information:**

The online version contains supplementary material available at 10.1186/s12879-023-08182-3.

## Background

Cancer patients have a higher risk and worse outcomes of infectious diseases, compared with healthy people [1]. Autopsy studies have indicated that infections may play a role in more than half of all cancer patient fatalities [1]. Importantly, infections often necessitate caregivers to postpone or withhold adequate cancer treatment, which may impair cancer outcomes. In recent years, there have been major changes in disease-causing microbial ecology, particularly in hospitals [2]. Bacteria and fungi are becoming increasingly resistant to antimicrobial drugs, and in Europe alone it is estimated that more than 33,000 people die each year from resistant microbes [3, 4]. Not only are microbes acquiring antimicrobial resistance, but the microbial spectrum is changing, with an increasing proportion of species with a propensity for intrinsic resistance [5]. Infections in cancer patients are increasingly often caused by resistant organisms, which threaten recent years’ advances in the treatment of cancer [6]. Recent studies has shown that cancer patients have a higher risk of contracting infections with antimicrobial-resistant organisms [7]. In other words, there is a pressing need to understand the changing epidemiology of bacterial and fungal infections among cancer patients, but also to design better preventive measures.

To adapt to this new reality, it is essential to understand the mechanisms by which infections occur and cause disease. The research that leads to the discovery and description of these mechanisms, as well as testing them in preliminary models, has been called ‘prognostic factor research’ by the PROGRESS Group [8]. Prognostic factor research forms the basis for more advanced risk stratification tools and scoring systems clinicians may use to guide anti-infective therapy. Such risk stratification tools or scoring systems are usually based on clinical prediction models, which are typically regression models where individual-level clinical data are used to predict a clinical outcome of interest [9, 10]. Some examples widely used in the management of infectious complications in cancer patients are the Multinational Association for Supportive Care in Cancer (MASCC) risk indices for febrile neutropenic patients or the Pitt bacteraemia score. These risk indices were developed in relatively small patient cohorts in the early 1990s [11, 12].

Because of the changing epidemiology, there is a need to update the multivariable regression models that both estimate and predict the risks associated with antimicrobial resistance in cancer care, like the additional risk of death attributable to resistance. In this work, it is important to build on already existing multivariable models and use factors that previously have been shown to be associated with the outcomes of interest. It is thus necessary to map the existing literature on such multivariable models to facilitate the use of current knowledge in future research. In this systematic review, we aimed to review multivariable models of resistant infections/colonisations and corresponding mortality in cancer patients, what risk factors have been included, and with what methodological approaches.

## Methods

### Protocol registration and reporting standards

To review what risk factors for resistant infections and/or carriage/colonisation (hereafter “infections/colonisations”) and corresponding mortality in cancer patients have been investigated, we conducted a systematic review employing a broad and extensive search for literature published from 1^st^ of January 2015 to 19^th^ of November 2021. It was not possible to separate studies on infection and colonisation, so these outcomes were combined. Although first coined in 1961, the term ‘risk factor’ remains an elusive term as it is used to describe any covariate associated with an outcome [13, 14]. We will here use the term ‘risk factor’ as a common designation for both causal risk factors and predictive risk markers, as this definition is often used in the primary studies included [15]. This systematic review was registered at PROSPERO (ID: CRD42021282769) [16] and follows the Preferred Reporting Items for Systematic Reviews and Meta-Analyses (PRISMA) guideline (checklist can be found in Supplementary Material 7) [17].

### Search strategy

Our search strategy was implemented in three steps. First, we performed preliminary searches in PubMed to identify some main keywords to be included and to get an overview of the size of the literature. We then performed a first search and, after screening and sorting the results of the search, we manually reviewed the records and references to discover keywords that were not covered by our initial search. We then performed a second search designed to expand the first search findings.

The first search was conducted by a research librarian (RT) from 22nd to 24th June, 2021. It consisted of terms covering all cancers and terms covering antibiotic resistance and infections. A spectrum of synonyms with appropriate truncations and proximity operators was used for searching title, abstract, and author keywords. In addition, controlled subject headings were searched when available. The search strategy was tailored to each database’s search interface. The search was run in the OVID MEDLINE and OVID Embase, in addition to EBSCO Cinahl and Web of Science Core Collection (Science Citation Index Expanded, Social Sciences Citation Index, Arts & Humanities Citation Index and Emerging Sources Citation Index—searched simultaneously). The strategies were limited to Danish, English, Norwegian, Spanish, and Swedish. They also included a time limit for publications from the 1^st^ of January 2015 onwards. A total of 25,881 records were retrieved. After removing duplicate records in EndNote, 14,153 references were identified.

The second search expanded on this by including specific antibiotics, resistance mechanisms, and bacteria and fungi often associated with either acquired resistance or high levels of intrinsic resistance. The terms covering antibiotics were variations of piperacillin/tazobactam, meticillin/methicillin, cephalosporin, carbapenem, aminoglycoside, gentamicin, amikacin, fluoroquinolone, linezolid, vancomycin, echinocandin, azole, colistin. The terms covering mechanisms of resistance or microbial properties associated with resistance were beta-lactamase/β-lactamase, extended-spectrum beta-lactamase/β-lactamase/betalactamase, carbapenemase, biofilm-producing, non-fermenting. The terms covering bacteria and fungi were *Pseudomonas aeruginosa, Acinetobacter spp., Acinetobacter baumannii, Stenotrophomonas maltophilia, Clostridium/Clostridioides difficile, Enterococcus faecium, Enterococcus faecalis*, coagulase-negative staphylococci, Candida non-albicans, *Candida auris, Aspergillus fumigatus*. Those were selected from a wide range of terms during a multidisciplinary meeting which led to a consensus. This search was run on 19^th^ of November 2021 in the same databases (except for Cinahl) and with the same limitations as the June version.

Search strategies can be found in Supplementary Materials 1 and 2.

### Study selection

The titles of all records were first screened by AD and LF to exclude any records that were not about antimicrobial resistance and cancer using the Rayyan tool [18]**.** We then screened all abstracts of the records where the subject was antimicrobial resistance and cancer to sort these into different study designs. The remaining original records with an observational study design were then read in full text by both AD and LF to see if they matched the eligibility criteria (Table 1). If there was uncertainty about the inclusion of a record, both authors discussed it until reaching a decision.

**Table 1:** Eligibility criteria

Inclusion criteria	Exclusion criteria
● Primary study with original data ● Observational study design (prospective/retrospective cohort study, case–control study, cross-sectional study) ● The study population consists only of cancer patients, haematological or otherwise, of any age, sex, or ethnicity, and in any institution in any country ● The study includes a multivariable model ● A resistant bacterial or resistant fungal infection is either a risk factor or an outcome ● The outcome is either infection (or colonisation) or death ● Published after 1st of January 2015 ● Published in English ● Studies in humans	● Any other publication type than a primary observational study ● Cancer is a covariate or comparator, not everyone in the study population has cancer ● Resistance to antimicrobial drugs is not included in the model (neither as a risk factor nor as an outcome) ● The outcome is not infection or mortality, e.g. distribution of bacteria or bacterial strains ● Studies where the causal assumption is reversed, so that infection causes cancer (e.g. increased risk of gastric cancer after *Helicobacter pylori* infection)

### Data extraction and statistical analysis

#### Data extraction

The risk of bias in all included studies was assessed by AD and LF separately in a blinded process using the National Institutes of Health study quality assessment tools [19], and an arbitration meeting was held where OK/JB acted as an arbiter to come to a consensus about the final risk of bias assessment. The guidance for the use of these tools was followed, but the tool was modified to include an item of whether the outcome was well defined in case–control studies.

Data extraction was then performed by AD and LF. We extracted the year of publication, the title of the study, the authors, the number of patients, the country of the study setting, the study aim statement, the patient population statement, the risk factors included in the final model, the microbial aetiology, whether the studies employed a screening for statistical significance, and the events per variable in the final model. This was included together with the risk of bias assessment and a short commentary in three different tables of all included studies, one for studies with an infection/colonisation outcome, one for studies with a mortality outcome and one for studies with both outcomes. These three tables may be found in the Supplementary Material S3, S4, and S5. The studies were checked against pre-specified criteria for a potential meta-analysis, being that models would have the same aetiology, outcome, and risk factors. These criteria were not met for any aetiology. To provide a genuine example of the heterogeneity of the models, population, variables and outcomes investigated, a qualitative in-depth example of the investigation of risk factors was reported. We chose researches that included vancomycin-resistant enterococci (VRE), as they represented a typical cross-section of the studies selected. Furthermore, VRE is a typical hospital-associated microbe that may readily be prevented.

#### Statistical analysis

A table describing the outcomes and extracted data on the country, microbial aetiology, patients, events, events per variable, variables screened, variables in the final model, and *p*-value-based variable selection were created. The table summarised the findings by presenting frequencies with percentages for categorical variables and medians with interquartile ranges for continuous variables. To summarise what risk factors have been investigated, we grouped the microbial aetiology into five large groups (*Clostridioides difficile*, fungi, Gram-negative bacteria, Gram-positive bacteria, and several bacteria/fungi) and categorised the risk factors. We then created a table of how many times the different types of risk factors had been included in the final multivariable model in the included studies for each of the large microbial aetiology groups. A full list of the risk factors and their respective categories can be found in the appendix. Analyses were performed in R using RStudio version 4.1.1 [20], and the scripts used to produce the results can be found on GitHub [21].

## Results

### Study selection

**Fig. 1:**
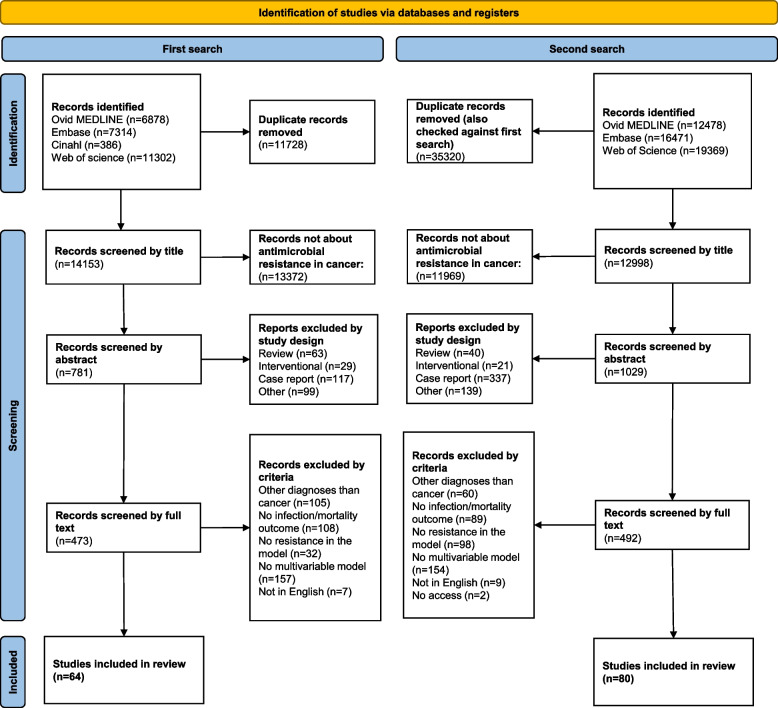
PRISMA flowchart of the study selection [17]

After excluding 47,048 duplicates, the two searches yielded a total of 27,151 unique records from a total of three major databases—Ovid MEDLINE, Ovid Embase, and Web of Science—in addition to the Cinahl database (EBSCO) (Fig. 1). Title screening for relevance to both antimicrobial resistance and cancer excluded 25,341 records, whereas most excluded records were only about cancer, the drug resistance of the cancer disease. Abstract screening for a non-observational study design excluded a further 845 records, where 103 had a review design, 50 had an interventional design, 454 were case reports, and 238 were miscellaneous, mostly commentary articles and conference abstracts. Finally, after full-text screening; 821 records were excluded, of which 165 included other diagnoses than cancer (including healthy individuals), 197 did not include an infection/colonisation or mortality outcome (often microbial distribution or endpoints like the length of stay), 130 did not explicitly include antimicrobial resistance in the model, 311 did not include a multivariable model, 16 were not in English, and we failed to gain access to two leaving 144 studies included.

### Study characteristics

Of the 144 studies, 55/144 (38%) had an infection/colonisation outcome, 66/144 (46%) had a mortality outcome, and 23/144 (16%) had both outcomes, all of which are listed in detail and cited in tables with all extracted data, including the investigated risk factors, in Supplementary Material S3, S4, and S5, respectively. In total, there were 23/144 (16%) studies of patients with solid cancers [22–44], 65/144 (45%) studies of patients with haematological cancers [45–109], and 56/144 (39%) studies with patients of both or unspecified cancer types [110–165]. Most studies selected (39/144, 27%) reported and modelled several bacteria and/or fungi that were tested for resistance towards several antimicrobials [22–37, 45–57, 110–119]. Eight of 144 (6%) reporting and modelling several microorganisms focused only on Gram-negative bacteria [58–62, 120–122] and 1/144 (1%) focused only on fungi [63]. Twelve of 144 (8%) studies studied the family *Enterobacterales* (especially *Escherichia coli* and *Klebsiella pneumoniae* combined), either ESBL- or carbapenemase-producing [38, 64–68, 123–128]. The most commonly studied single organism was *Clostridioides difficile* with 27/144 (19%) studies [39–41, 69–81, 129–139]. Also studied were three non-fermenters with 5/144 (3%) studies about *Acinetobacter baumannii* [82, 140–143], 4/144 (3%) studies about *Pseudomonas aeruginosa* [83, 84, 144, 145], and 5/144 (3%) studies about *Stenotrophomonas maltophilia* [85–88, 146], often specified as either multidrug- or extensively drug-resistant. We also found several studies focusing on four well-known healthcare-associated bacteria, with 11/144 (8%) focusing on VRE [89–98, 147, 148], 7/144 (5%) focusing on carbapenemase-producing *K. pneumoniae* [99–102, 149–151], 2/144 (1%) focusing on methicillin-resistant *Staphylococcus aureus* [42, 152], and 6/144 (4%) focusing on extended-spectrum beta-lactamase-producing *Escherichia coli* [103, 104, 153–156]. We also found that fungi typically resistant to antifungals were studied, 3/144 (2%) studies about *Aspergillus* spp. [43, 105, 106] and 9/144 (6%) studies about *Candida* non-albicans [44, 107, 157–163], respectively. Finally, there was one of 144 (1%) studies for each of the microbes *Staphylococcus epidermidis* (with linezolid-resistance) [108], *Bacillus* spp. [164], *Streptococcus pneumoniae* (several resistance mechanisms) [165], and *Aeromonas sobria* [109].

### Summary of findings

The most common microbial aetiologies were several bacteria/fungi (39, 25%), followed by *C. difficile* (27, 19%) and Enterobacteriaceae (12, 8%) (Table 2). The most common country for the study setting was the United States of America (USA) (40, 28%), followed by China (15, 10%), and Italy (11, 8%). The selected studies included a median of 200 patients (IQR 102–338) and 46 events (IQR 25–83.5). 103 (72%) studies used a *p*-value-based variable selection, either bivariable screening or stepwise regression. These studies screened a median of 16 variables (IQR 9–27) and included a median of 7 variables in the final (and largest) model. The median events per variable was close to 7.

**Table 2:** Summary of findings

Characteristics	Frequencies
**Outcome**	**% (n)**
Infection/colonisation	38 (55)
Death	46 (66)
Both	16 (23)
**Country/setting**	**% (n)**
USA	28 (40)
China	10 (15)
Italy	8 (11)
Spain	7 (10)
Germany	6 (9)
South Korea	6 (9)
**Microbial aetiology**	**% (n)**
Several bacteria and / or fungi	27 (39)
*C. difficile*	19 (27)
Enterobacteriaceae	8 (12)
Vancomycin-resistant enterococci	8 (11)
Carbapenemase-producing *K. pneumoniae*	5 (7)
**Patients (median)**	200 (IQR 102–338)
**Events (median)**	46 (IQR 25–84)
**Events per variable (median)**	7 (IQR 4–13)
**Variables screened (median)**	16 (IQR 9–27)
**Variables in the final model (median)**	7 (IQR 4–10)
**Stepwise regression**	**% (n)**
	72 (103)
**Total**	**144**

The most commonly investigated risk factors in models of resistant infection and/or colonisation in cancer patients were antibiotic use, with a total of 118 occurrences in the included studies (Table 3). In the models of mortality, however, the most commonly investigated risk factors were related to infection with a total of 208 occurrences. A full list of the risk factors and their respective categories may be found in Supplementary material S6.

**Table 3:** Categories of risk factors investigated in the final, multivariable model in the included studies, by large groups of microbial aetiology

Infection/colonisation		Mortality	
**Risk factors by microbial aetiology**	Count	**Risk factors by microbial aetiology**	Count
**Clostridioides difficile**		**Clostridioides difficile**	
Basic characteristics	29	Basic characteristics	10
Antibiotic use	28	Comorbidities/clinical condition	8
Cancer-related	18	Hospital-related	6
Comorbidities/clinical condition	14	Infection-related	6
Hospital-related	14	Other	3
**Fungi**		**Fungi**	
Antifungal use	7	Infection-related	21
Basic characteristics	7	Hospital-related	9
Other	6	Basic characteristics	7
Infection-related	6	Comorbidities/clinical condition	7
Cancer-related	4	Antibiotic use	6
**Gram-negative bacteria**		**Gram-negative bacteria**	
Antibiotic use	65	Infection-related	71
Infection-related	35	Comorbidities/clinical condition	40
Hospital-related	23	Cancer-related	31
Basic characteristics	21	Antibiotic use	27
Cancer-related	15	Basic characteristics	24
**Gram-positive bacteria**		**Gram-positive bacteria**	
Antibiotic use	14	Infection-related	14
Infection-related	11	Cancer-related	11
Cancer-related	7	Basic characteristics	8
Basic characteristics	5	Neutropenia	6
Chemotherapy/immunosuppressants	5	Antibiotic use	5
**Several bacteria and/or fungi**		**Several bacteria and/or fungi**	
Transplantation-related	5	Infection-related	96
Antibiotic use	4	Comorbidities/clinical condition	39
Laboratory findings (non-microbiological)	4	Cancer-related	31
Surgery-related	4	Basic characteristics	31
Comorbidities/clinical condition	2	Antibiotic use	27

### In-depth example of VRE

As a model organism, we explored how risk factors for and of VRE in cancer patients were described and modelled in the selected studies. Of our selection, 46/144 (32%) of the studies mentioned either *Enterococcus* spp., *E. faecium*, or *E. faecalis*, which was either described as vancomycin-resistant or “multidrug-resistant” (sometimes not further specified), or where the resistance mechanism was not described at all. Most of the studies (30/46, 65%) that mentioned the organism included it in a larger group, like Gram-positive bacteria or multidrug-resistant organisms, which hampered a specific focus on VRE [22–34, 45–53, 110–117].

Eight studies modelled the risk of being either colonised or infected with VRE. The number of patients included ranged from 72 to 342, and the variables screened ranged from 6 to 46. One study focused on patients undergoing liver transplantation for hilar cholangiocarcinoma, and seven focused on haematological cancer patients. Although a plethora of risk factors was investigated, five of the studies selected variables based on their corresponding *p*-values in bivariable analyses, and as such dropped several variables from the analysis due to their failure to achieve the *p*-value criterion. The authors did not necessarily conclude that all risk factors investigated in the multivariable model were of importance. The risk of bias was rated low for three, medium for five, while none had a “fatal flaw”. However, five of eight studies had less than ten events per variable in the final model. Among the common conclusions from the studies of VRE infection or colonisation, four concluded that antibiotic exposure was a risk factor for VRE infection or colonisation, and two highlighted neutropenia. None of the antibiotic exposure risk factors was the same (one was vancomycin, one was carbapenem, one was general antibiotic exposure and one was daptomycin). When looking at the findings in detail, Aktürk et al. found that severe neutropenia and previous bacteraemia with another pathogen may increase the risk of progressing from VRE colonisation to VRE infection in paediatric haematological cancer patients [147]. In 2015, Ford et al. found that severe neutropenia and the number of stools per day were associated with VRE bloodstream infections in leukaemia patients, and in 2019 some of the same authors concluded that VRE colonisation rates fell when the hospital started using less carbapenem [89, 90]. Herc et al. reported that only previous daptomycin exposure was associated with daptomycin-resistant VRE infections in patients with haematological malignancies [91]. Hefazi et al. found that VRE colonisation is associated with VRE infection in stem cell transplantation patients and Ramanan et al. found that VRE colonisation pre-transplantation was associated with any infection post-transplantation [35, 92]. Heisel et al. found that cephalosporin use and intravenous vancomycin were associated with VRE infections in patients with acute myeloid leukaemia or myelodysplastic syndrome undergoing intensive induction therapy, and finally, Klein et al. found that in multiple myeloma patients, granulocyte-colony stimulating factor was associated with fewer VRE cases than antibiotic prophylaxis [93, 94].

Another eight studies analyse the deaths associated with VRE in cancer patients. The number of patients included ranged from 95 to 1424, and the variables screened ranged from 11 to 56, although the exact number was indeterminable for one of the studies. Six studies selected variables based on a specified *p*-value threshold, but for one study the method for variable selection could not be determined. We assessed the risk of bias among these studies and found four studies at low risk and four studies at a medium risk of bias. Of the eight studies, six had less than 10 events per variable in the final model. The studies had few conclusions about risk factors for mortality among cancer patients associated with VRE in common. However, three studies found that VRE bacteremia was a risk factor for death and two studies found no risk factors after running their model. When looking at the findings in detail, Akhtar et al. found that only shock (not further specified) was associated with the difference in mortality between VRE and vancomycin-susceptible enterococci bacteraemia in cancer patients [148]. Kamboj et al. did not find any factors that were associated with higher mortality in stem cell transplantation patients with VRE bacteraemia [95]. Kern et al. modelled the mortality associated with enterococcal bacteraemia in haematological cancer patients but did not specify vancomycin resistance [54]. Kirkizlar et al. found that in leukaemia patients colonised with VRE, a low neutrophil count and coinfection were associated with increased mortality [96]. Mendes et al. included VRE in a bivariable screening but discarded the factor as it did not meet the criterion of *p* < 0.1 [55]. Ornstein et al. found that leukaemia patients with a VRE bacteraemia at the induction of chemotherapy had poorer survival than patients with other bloodstream infections [97]. Papanicolaou et al. found that VRE bacteraemia increased the mortality in patients receiving their first stem cell transplantation, but did not disclose how variables were selected for the multivariable model [98]. Finally, Pugliese et al. modelled the risk of mortality associated with several bacteria in leukaemia inpatients, among them *Enterococcus* spp. (no vancomycin resistance mentioned), but did not find an association [56].

## Discussion

In our systematic review of studies with multivariable models of risk factors for infection/colonisation and mortality associated with antimicrobial resistance in cancer patients, we selected 144 studies that were eligible for inclusion. Most studies focused on haematological cancer patients and explored a host of different microbes. Studies on infection/colonisation with resistant microbes as outcomes most often investigated risk factors relating to antibiotic use, while studies with mortality as an outcome often included risk factors relating to the infection itself. Studies often had small sample sizes, screened a large number of variables, and used *p*-value-based methods for variable selection like bivariable screening or stepwise regression. In general, the models were highly heterogeneous, with nuances in the study populations and microbial aetiologies, and major differences in which risk factors were modelled, as we have exemplified through the in-depth example of VRE in cancer patients.

The issues with heterogeneity when performing a systematic review of basic prognostic factor research have been described before [166]. Although it is not possible to infer which factors are most important, we found that known general risk factors for infections like immunosuppression and specific risk factors for resistant infections like previous antibiotic use were recurring, as other non-systematic reviews do [1, 5–7]. A comprehensive list of these factors can be found in the supplementary material. To the best of our knowledge, no previous systematic review has investigated risk factors for any resistant infections in cancer patients. However, three systematic reviews have looked at risk factors for methicillin-resistant *S. aureus*, extended-spectrum β-lactamase-producing *Enterobacteriaceae*, and vancomycin-resistant enterococci, specifically [167–169]. These studies were able to pool the prevalence of such infections highlighting some common risk factors, providing a good basis for future research. Some systematic reviews also investigated the changing epidemiology of antimicrobial-resistant infections in cancer patients without a particular focus on risk factors [5, 170].

Some conservative choices were made in the selection process which may have reduced the final number of studies included. Some studies were excluded because study participants had other diagnoses than cancer, e.g. recipients of haematopoietic stem cell transplantation due to non-malignant haematological disorders [171, 172]. Several of these studies did not describe the full diagnostic panorama, even though comprehensive description of patient characteristics remain an important backbone in epidemiological research. Other studies did not explicitly model resistant microbes by including antimicrobial resistance as a variable or an outcome [173, 174]. Given the rapid increase in antimicrobial resistance, current research in the epidemiology of infectious diseases in healthcare settings should include detailed data on antimicrobial resistance. The most common reason for excluding observational studies during full-text reading was the lack of a multivariable model [175, 176]. These studies seemed to have a low number of patients, and a multivariable model may have been avoided due to low statistical power. As discussed by the PROGRESS Research Group in their recommendations for prognostic factor research, the discovery and investigation into new prognostic factors should rely on multivariable modelling to discern these from factors already known to be of importance, and to provide a basic adjustment of confounders [8]. Studies simply testing whether factors differ between groups often rely heavily on null-hypothesis statistical significance testing and are subject to the multiple comparison problem [177]. Although exploratory studies are important, such studies sometimes either test risk factors that are already known to be associated with the outcome, test factors in too small samples, or test too many factors at once. This may lead to wrong inferences due to known issues such as multiple comparisons, sparse data bias or winner’s curse inflating effect sizes [178, 179]. An alternative can be to establish larger research collaborations that can pool data into larger cohorts as we found several examples of [63, 144]. Worth noting, we found two clinical prediction models. In one of them, the IRONIC group developed a scoring system for the risk of multidrug resistance in bloodstream infections by *P. aeruginosa*, and in another, Colombian researchers developed a scoring system for the risk of ESBL-producing *Enterobacteriacaea* [128, 144].

Studies were assessed for their risk of bias using the NIH Quality Assessment tool, in which we found several recurring issues. Most studies did not include a sample size calculation or described the blinding of exposure assessors. Differences in the risk of bias assessments were often determined by allowing continuous variables to be treated continuously or by the correct definition of the exposure. We also found that several studies lacked a reliable definition of the outcome, in particular the definition of mortality. As discussed by the NIH Quality Assessment reviewers, even though death as an outcome seems to be objective by nature (i.e. researchers rely on “face validity”), even this outcome should be clearly defined. Such a definition should include information on particulars like from which register or medical record information about the death was collected, if the patient perished within or outside of the institution, and if the latter was the case, who reported the death. Most studies did have a sufficient follow-up time to capture infectious disease outcomes (in particular mortality), but these follow-up times varied greatly for all studies. However, the NIH Quality Assessment tool included items that were not relevant to all studies (e.g. “the adjustment of confounders” in a prediction context). The issue with finding good quality assessment tools for prognostic factor research has been described previously [180]. Systematic reviews of clinical prediction models should use other more specialised tools like PROBAST [181].

We chose to summarise the research by describing an in-depth example of studies on VRE, which provided us with a representative cross-section in terms of the patient population, sample size, methodological approaches, and the number of variables. This example showed how heterogeneity in approaches may hamper the ability to build on this research, either through the development of more comprehensive predictive models, through pooling or meta-analysis. Most studies studying resistant enterococci group the bacteria together with other bacteria and/or fungi. Furthermore, authors often study the bacteria in a highly selected patient population, in which it is difficult to infer how patient characteristics relate to the risk factors studied, either from a table of characteristics or a regression table. However, the major source of heterogeneity that reduces the ability to build on the research is the way in which variables are selected.

We find that throughout the entire material, there was widespread use of either stepwise regression or a bivariable screening of variables as a method for variable selection in regression models, where variables that achieve some pre-specified *p*-value threshold are included in the final model. This method is, however, not recommended neither for estimating the effect of an exposure or predicting an outcome [10, 182–184]. In short, the reason is its reliance on *p*-values as a criterion for variable selection, although the *p*-value is not an indicator of a causal relationship or how well the model can predict an outcome. A factor confounding a causal effect may not be statistically significant, and there may be situations in which a variable that is not statistically significant in a bivariable analysis may increase the predictive power of a multivariable model. Consequently, including all candidate factors in a multivariable model and testing them by statistical significance may not be a valid method of discovering new prognostic factors. Unfortunately, there is no alternative to these methods based on statistical significance that is as simple and practical, or as automated and data-driven. The basis for all modelling is a theoretical understanding of covariates and outcomes and the relationship between them. All in all, it is difficult and maybe even impossible to summarise what risk factors are shown to be of relevance in this literature. Simply counting how many times a certain risk factor is found to be statistically significant tells us little of its relative importance.

The strengths of our study are the large scope of our searches, which has resulted in a comprehensive overview of the current state of this research topic. However, there are several limitations to our study. First, we only searched for studies where any type of antimicrobial resistance was mentioned in the title, abstract, controlled vocabulary, or keywords, which may have excluded some studies that only mention infections in broader terms, but still model the risk of contracting resistant infections or any potential outcomes of such infections. Furthermore, we narrowed our inclusion criteria to studies that model either infection/colonisation or mortality as an outcome, but this does not fully cover how resistant microbes may increase the disease burden among cancer patients, like repeated hospitalisations, increased costs and/or increased length of hospital stays. Several of the studies that were excluded mainly had patients with a haematological malignancy, but also some patients with aplastic anaemia or other haematological disorders. Other studies did not explicitly include (acquired or intrinsic) resistance in the models, although they may have included resistance in other implicit ways, like the failure of an empirical antibiotic cure. Patients with other haematological disorders or other infectious aetiologies may have similar risk profiles, and their exclusion may have unreasonably narrowed the scope of the review. We also searched for records in Swedish, Danish and Spanish, but records in these languages were not included to ensure this systematic review would be available and reproducible for all readers. As many of the studies we excluded were from countries where English is not the native language, the exclusion of other languages may represent a limitation of our review.

## Conclusions

In this systematic review, we found a great level of heterogeneity in the approach to studying risk factors for resistant infections/colonisations and mortality due to resistant infections among cancer patients. We argue that it’s difficult or even impossible to use these models to infer which risk factors are of importance. This is due to differences in the patient populations selected, and the different ways of grouping microbes. Furthermore, studies on this subject often have a small sample size and use *p*-value-based variable selection methods, which lead to very diverse models. The consequence of this heterogeneity is not only that the literature is unprepared for either a meta-analysis or a pooled analysis. It also means that it is difficult to use this research to understand the mechanisms by which resistant microbes cause disease in cancer patients, and thus that it is difficult for clinicians to use the research to guide their prevention of such conditions. This represents a serious shortcoming of this body of literature. There is a need to develop and adhere to more standardised protocols when investigating new risk factors. Such protocols should include a clear aim of what risk factors are to be explored and build on existing literature, e.g. by selecting similar patient populations and being careful to include factors previously shown to be of importance regardless of their *p*-value in a bivariable screening.

## Supplementary Information

**Additional file 1: Supplementary material 1.** Search strategy of the first search.

**Additional file 2: Supplementary material 2.** Search strategy of the second search.

**Additional file 3: Table S3.** All articles included in the systematic review with an infection/colonisation outcome.

**Additional file 4: Table S4.** All articles included in the systematic review with a mortality outcome.

**Additional file 5: Table S5.** All articles included in the systematic review with both an infection/colonisation outcome and a mortality outcome.

**Additional file 6: Table S6.** All risk factors investigated in the final, multivariable model of the included studies, and their respective categories.

**Additional file 7: Supplementary material 7.** PRISMA 2020 Main Checklist. PRISMA Abstract. Checklist.

## Data Availability

All data extracted or generated and then analysed are included in the article or the supplementary material.
