# Supplementary material for: Clinical outcomes of antimicrobial resistance in cancer patients: a systematic review of multivariable models
Source: BMC Infect Dis. 2023 Apr 18;23:247. doi: 10.1186/s12879-023-08182-3 (PMC10114324; doi:10.1186/s12879-023-08182-3)
Supplement: Supplementary file 1 — Additional file 1: Supplementary material 1. Search strategy of the first search. [file 12879_2023_8182_MOESM1_ESM.docx]

# Supplementary material 1

First search

**Database: Ovid MEDLINE(R) and Epub Ahead of Print, In-Process, In-Data-Review & Other Non-Indexed Citations, Daily and Versions(R) <1946 to June 21, 2021>**

**Date:** 22.06.21

**Hits:** 6878

| 1 | exp Neoplasms/ | 3485612 |
| --- | --- | --- |
| 2 | (tumor? or tumour? or cancer? or neoplas* or paraneoplastic or precancer* or cyst? or malignanc* or carcinogenes#s or oncogenes#s or tumorigenes#s or cocarcinogenes#s or metastas#s or metastatic or incidentaloma?).tw,kf. | 3441687 |
| 3 | 1 or 2 | 4533774 |
| 4 | exp Drug Resistance, Microbial/ or exp Drug Resistance, Bacterial/ | 170727 |
| 5 | (((antibacterial or bacterial or bacterium or antibiotic or antimicrobial or microbial or multidrug or multiple drug) adj2 (resistance or resistant)) or antibiotic non-susceptibility or antibiotic nonsusceptibility).tw,kf. | 136901 |
| 6 | 4 or 5 | 254631 |
| 7 | 3 and 6 | 22036 |
| 8 | limit 7 to (danish or english or interlingua or multilingual or norwegian or spanish or swedish) | 20721 |
| 9 | limit 8 to yr="2015 -Current" | 6878 |

**Database: Embase <1974 to 2021 June 22>**

**Date:** 23.06.21

**Hits:** 7314

| 1 | exp neoplasm/ | 4730064 |
| --- | --- | --- |
| 2 | (tumor? or tumour? or cancer? or neoplas* or paraneoplastic or precancer* or cyst? or malignanc* or carcinogenes#s or oncogenes#s or tumorigenes#s or cocarcinogenes#s or metastas#s or metastatic or incidentaloma?).tw,kw. | 4525726 |
| 3 | 1 or 2 | 5821589 |
| 4 | exp antibiotic resistance/ | 177653 |
| 5 | (((antibacterial or bacterial or bacterium or antibiotic or antimicrobial or microbial or multidrug or multiple drug) adj2 (resistance or resistant)) or antibiotic non-susceptibility or antibiotic nonsusceptibility).tw,kw. | 167050 |
| 6 | 4 or 5 | 267257 |
| 7 | 3 and 6 | 28672 |
| 8 | limit 7 to (danish or english or norwegian or polyglot or spanish or swedish) | 26746 |
| 9 | limit 8 to yr="2015 -Current" | 9903 |
| 10 | limit 9 to embase | 7314 |

**Database: Web of Science (Science Citation Index Expanded (SCI-EXPANDED) --1987-present, Social Sciences Citation Index (SSCI) --1987-present, Arts & Humanities Citation Index (A&HCI) --1987-present, Emerging Sources Citation Index (ESCI) --2015-present)**

**Date:** 24.06.21

**Hits:** 11,302

| 1 | TS=("tumor$" or "tumour$" or "cancer$" or "neoplas*" or "paraneoplastic" or "precancer*" or "cyst$" or "malignanc*" or "carcinogenes?s" or "oncogenes?s" or "tumorigenes?s" or "cocarcinogenes?s" or "metastas?s" or "metastatic" or "incidentaloma$") | 3,746,108 |
| --- | --- | --- |
| 2 | TS=((("antibacterial" or "bacterial" or "bacterium" or "antibiotic " or "antimicrobial" or "microbial" or "multidrug" or "multiple drug") NEAR/1 ("resistance" or "resistant")) or "antibiotic non-susceptibility" or "antibiotic nonsusceptibility") | 172,855 |
| 3 | (#2 AND #1)  AND LANGUAGE: (English OR Danish OR Multiple Languages OR Norwegian OR Spanish OR Swedish) | 11,302 |
