# Supplementary material for: Clinical outcomes of antimicrobial resistance in cancer patients: a systematic review of multivariable models
Source: BMC Infect Dis. 2023 Apr 18;23:247. doi: 10.1186/s12879-023-08182-3 (PMC10114324; doi:10.1186/s12879-023-08182-3)
Supplement: Supplementary file 2 — Additional file 2: Supplementary material 2. Search strategy of the second search. [file 12879_2023_8182_MOESM2_ESM.docx]

# Supplementary material 2

Second search

**Database: Ovid MEDLINE(R) and Epub Ahead of Print, In-Process, In-Data-Review & Other Non-Indexed Citations, Daily and Versions(R) <1946 to November 18, 2021>**

**Date:** 19.11.21

**Hits:** 12 478

| 1 | exp Neoplasms/ | 3573100 |
| --- | --- | --- |
| 2 | (tumor? or tumour? or cancer* or neoplas* or paraneoplas* or preneoplas* or precancer* or premalign* or malign* or carcinogenes#s or oncogenes#s or tumorigenes#s or cocarcinogenes#s or metastas#s or metastatic or incidentaloma? or leukemia? or leucemia? or lymphoma? or sarcoma? or carcinoma* or melanoma? or glioma ? or blastoma?).tw,kf. | 3958905 |
| 3 | 1 or 2 | 4744972 |
| 4 | exp Drug Resistance, Microbial/ or exp Drug Resistance, Bacterial/ | 174771 |
| 5 | ((antibacterial or bacterial or bacterium or antibiotic or antifungal or antimicrobial or microbial or multidrug or multiple drug or multiple antibiotics or penicillin or meth#cillin? or met#cillin? or cephalosporin* or cephalosporanic acid? or cefepim? or cefuroxime or cefotaxim? or ceftazidime or ceftriaxon? or cefixime or ceftaroline or carbapenem? or aminoglycoside? or gentam#cin? or amikacin? or fluoroquinolone? or linezolid? or vancomycin? or echinocandin? or azole? or colistin? or colimycin? or polymyxin e or intrinsic* or penicillin or macrolide or tobramycin? or fluconazol? or voricanozole or posaconazole or caspofungin or anidulafungin or micafungin or ciproflox* or ciproxin or levofloxacin or clindamycin? or dalacin or clarithromycin or CLAR or erythromycin or azt?reonam or azactam) adj4 (resistance or resistant or nonsusceptib* or insusceptib* or non-susceptib* or susceptibility)).tw,kf. | 235262 |
| 6 | (piperacillin adj3 tazobactam adj4 resistant).tw,kf. | 202 |
| 7 | beta-Lactamases/ or Methicillin-Resistant Staphylococcus aureus/ or Vancomycin-Resistant Enterococci/ or Vancomycin-Resistant Staphylococcus aureus/ | 40508 |
| 8 | (b*-lactamase? or "ec 3.5.2.6" or beta lactam?hydrolase or cefinase or ESBL or carbapenemase? or biofilm or non-fermenting).tw,kf. | 88504 |
| 9 | Pseudomonas aeruginosa/ or (pseudomonas aeruginosa or "P. aeruginosa").tw,kf. | 74856 |
| 10 | Acinetobacter/ or (acinetobacter* or "A. baumanii").tw,kf. | 20486 |
| 11 | Stenotrophomonas Maltophilia/ or ((Stenotrophomonas or Pseudomonas or Xanthomonas) adj malt#philia).tw,kf. | 3477 |
| 12 | Clostridioides difficile/ or ((clostridium or clostridioides) adj difficil*).tw,kf. | 17332 |
| 13 | Enterococcus faecium/ or ((enterococcus or streptococcus) adj faecium).tw,kf. | 7433 |
| 14 | (candida adj1 (non albicans or auris)).tw,kf. | 1779 |
| 15 | Aspergillus fumigatus/ or ((aspergillus or neosartorya) adj fumigat*).tw,kf. | 12302 |
| 16 | or/4-15 | 456787 |
| 17 | 3 and 16 | 35096 |
| 18 | limit 17 to (danish or english or interlingua or multilingual or norwegian or spanish or swedish) | 32942 |
| 19 | limit 18 to yr="2015 -Current" | 12478 |

**Database: Embase <1974 to 2021 November 17>**

**Date:** 19.11.21

**Hits:** 16471

| 1 | exp Neoplasm/ | 4853484 |
| --- | --- | --- |
| 2 | (tumor? or tumour? or cancer* or neoplas* or paraneoplas* or preneoplas* or precancer* or premalign* or malign* or carcinogenes#s or oncogenes#s or tumorigenes#s or cocarcinogenes#s or metastas#s or metastatic or incidentaloma? or leukemia? or leucemia? or lymphoma? or sarcoma? or carcinoma* or melanoma? or glioma ? or blastoma?).tw,kf. | 5177268 |
| 3 | 1 or 2 | 6060075 |
| 4 | exp Antibiotic Resistance/ or Antifungal resistance/ or Antifungal susceptibility/ or Multidrug resistance/ or Extensive Drug Resistance/ or Antifungal susceptibility/ | 229586 |
| 5 | ((antibacterial or bacterial or bacterium or antibiotic or antifungal or antimicrobial or microbial or multidrug or multiple drug or multiple antibiotics or penicillin or meth#cillin? or met#cillin? or cephalosporin* or cephalosporanic acid? or cefepim? or cefuroxime or cefotaxim? or ceftazidime or ceftriaxon? or cefixime or ceftaroline or carbapenem? or aminoglycoside? or gentam#cin? or amikacin? or fluoroquinolone? or linezolid? or vancomycin? or echinocandin? or azole? or colistin? or colimycin? or polymyxin e or intrinsic* or penicillin or macrolide or tobramycin? or fluconazol? or voricanozole or posaconazole or caspofungin or anidulafungin or micafungin or ciproflox* or ciproxin or levofloxacin or clindamycin? or dalacin or clarithromycin or CLAR or erythromycin or azt?reonam or azactam) adj4 (resistance or resistant or nonsusceptib* or insusceptib* or non-susceptib* or susceptibility)).tw,kf. | 287384 |
| 6 | (piperacillin adj3 tazobactam adj4 resistant).tw,kf. | 334 |
| 7 | beta-Lactamase/ or Extended Spectrum Beta Lactamase/ or Carbapenemase/ or Carbapenemase Producing Enterobacteriaceae/ or Carbapenem resistance/ or Methicillin Resistant Staphylococcus aureus/ or Vancomycin-Resistant Enterococcus/ or Vancomycin Resistant Staphylococcus aureus/ | 85966 |
| 8 | (b*-lactamase? or "ec 3.5.2.6" or beta lactam?hydrolase or cefinase or ESBL or carbapenemase? or biofilm or non-fermenting).tw,kf. | 105594 |
| 9 | Pseudomonas aeruginosa/ or (pseudomonas aeruginosa or "P. aeruginosa").tw,kf. | 122215 |
| 10 | Acinetobacter/ or (acinetobacter* or "A. baumanii").tw,kf. | 29911 |
| 11 | Stenotrophomonas Maltophilia/ or ((Stenotrophomonas or Pseudomonas or Xanthomonas) adj malt#philia).tw,kf. | 7160 |
| 12 | Clostridioides difficile/ or ((clostridium or clostridioides) adj difficil*).tw,kf. | 24295 |
| 13 | Enterococcus faecium/ or ((enterococcus or streptococcus) adj faecium).tw,kf. | 12422 |
| 14 | Candida auris/ or (candida adj1 (non albicans or auris)).tw,kf. | 2531 |
| 15 | Aspergillus fumigatus/ or ((aspergillus or neosartorya) adj fumigat*).tw,kf. | 20302 |
| 16 | or/4-15 | 575608 |
| 17 | 3 and 16 | 57855 |
| 18 | limit 17 to (danish or english or norwegian or polyglot or spanish or swedish) | 54246 |
| 19 | limit 18 to yr="2015 -Current" | 23248 |
| 20 | limit 19 to embase | 16471 |

**Database: Web of Science Core Collection: Science Citation Index Expanded (SCI-EXPANDED) --1987-present, Social Sciences Citation Index (SSCI) --1987-present, Arts & Humanities Citation Index (A&HCI) --1987-present, Emerging Sources Citation Index (ESCI) --2015-present**

**Date:** 19.11.21

**Hits:** 19 369

| 14 | (#13) AND LA=(English OR Danish OR Multiple Languages OR Norwegian OR Spanish OR Swedish) | 19,369 |
| --- | --- | --- |
| 13 | (#12) AND PY=(2015-2021) | 19,528 |
| 12 | #1 and #11 | 46,063 |
| 11 | #2 or #3 or #4 or #5 or #6 or #7 or #8 or #9 or #10 | 454,205 |
| 10 | TS=(("aspergillus" or "neosartorya") NEAR/0 "fumigat*") | 13,687 |
| 9 | TS=("candida" NEAR/0 ("non albicans" or "auris")) | 1,906 |
| 8 | TS=(("enterococcus" or "streptococcus") NEAR/0 "faecium") | 8,677 |
| 7 | TS=(("clostridium" or "clostridioides") NEAR/0 "difficil*") | 20,940 |
| 6 | TS=(("Stenotrophomonas" or "Pseudomonas" or "Xanthomonas") NEAR/0 "malt?philia") | 4,062 |
| 5 | TS=("acinetobacter*" or "A. baumanii") | 23,475 |
| 4 | TS=("pseudomonas aeruginosa" or "P. aeruginosa") | 88,969 |
| 3 | TS=("b*-lactamase$" or "ec 3.5.2.6" or "beta lactam hydrolase" or "beta lactamhydrolase" or "cefinase" or "ESBL" or "carbapenemase$" or "biofilm" or "non-fermenting") | 118,459 |
| 2 | TS=(("antibacterial" or "bacterial" or "bacterium" or "antibiotic$" or "antifungal" or "antimicrobial" or "microbial" or "multidrug" or "multiple drug" or "multiple antibiotics" or "penicillin" or "meth?cillin$" or "met?cillin$" or "cephalosporin*" or "cephalosporanic acid$" or "cefepim$" or "cefuroxime" or "cefotaxim$" or "ceftazidime" or "ceftriaxon$" or "cefixime" or "ceftaroline" or "carbapenem$" or "aminoglycoside$" or "gentam?cin$" or "amikacin$" or "fluoroquinolone$" or "linezolid$" or "vancomycin$" or "echinocandin$" or "azole$" or "colistin$" or "colimycin$" or "antibacterial" or "bacterial" or "bacterium" or "antibiotic" or "antifungal" or "antimicrobial" or "microbial" or "multidrug" or "multiple drug" or "multiple antibiotics" or "penicillin" or "meth?cillin$" or "met?cillin$" or "cephalosporin*" or "cephalosporanic acid$" or "cefepim$" or "cefuroxime" or "cefotaxim$" or "ceftazidime" or "ceftriaxon$" or "cefixime" or "ceftaroline" or "carbapenem$" or "aminoglycoside$" or "gentam?cin$" or "amikacin$" or "fluoroquinolone$" or "linezolid$" or "vancomycin$" or "echinocandin$" or "azole$" or "colistin$" or "colimycin$" or "polymyxin e" or "intrinsic*" or "penicillin" or "macrolide" or "tobramycin$" or "fluconazol$" or "voricanozole" or "posaconazole" or "caspofungin" or "anidulafungin" or "micafungin" or "ciproflox*" or "ciproxin" or "levofloxacin" or "clindamycin$" or "dalacin" or "clarithromycin" or "CLAR" or "erythromycin" or "azt$reonam" or "azactam" or "polymyxin e" or "intrinsic*" or "penicillin" or "macrolide" or "tobramycin$" or "fluconazol$" or "voricanozole" or "posaconazole" or "caspofungin" or "anidulafungin" or "micafungin" or "ciproflox*" or "ciproxin" or "levofloxacin" or "clindamycin$" or "dalacin" or "clarithromycin" or "CLAR" or "erythromycin" or "azt$reonam" or "azactam") NEAR/3 ("resistance" or "resistant" or "nonsusceptib*" or "insusceptib*" or "non-susceptib*" or "susceptibility")) | 274,926 |
| 1 | TS=("tumor$" or "tumour$" or "cancer$" or "neoplas*" or "paraneoplas*" or "preneoplas*" or "precancer*" or "premalign*" or "malign*" or "carcinogenes?s" or "oncogenes?s" or "tumorigenes?s" or "cocarcinogenes?s" or "metastas?s" or "metastatic" or "incidentaloma$" or "leukemia$" or "leucemia$" or "lymphoma$" or "sarcoma$" or "carcinoma*" or "melanoma$" or "glioma$" or "blastoma$") | 4,410,329 |
