# Supplementary material for: Clinical outcomes of antimicrobial resistance in cancer patients: a systematic review of multivariable models
Source: BMC Infect Dis. 2023 Apr 18;23:247. doi: 10.1186/s12879-023-08182-3 (PMC10114324; doi:10.1186/s12879-023-08182-3)
Supplement: Supplementary file 3 — Additional file 3: Table S3. All articles included in the systematic review with an infection/colonisation outcome. [file 12879_2023_8182_MOESM3_ESM.docx]

# Supplementary material 3

**Table S3 - All included articles in the systematic review with an infection/colonisation outcome**

| **Year** | **Title** | **Authors** | **Number of patients** | **Number of events in the final (and largest) model** | **Country/setting** | **Study aim statement** | **Patient population** | **Factors included in the final model** | **Microbial etiology and resistance** | **Risk of bias grading (NIH tool)** | **Bivariable screening/stepwise regression** | **Events per variable in the final (and largest) model** | **Number of variables included in the tested/screened/initial model** | **Number of variables included in the final (and largest) model** | **Comments** |
| --- | --- | --- | --- | --- | --- | --- | --- | --- | --- | --- | --- | --- | --- | --- | --- |
| 2019 | Recurrent *Clostridium difficile* infection is associated with treatment failure and prolonged illness in cancer patients | Abu-Sbeih, Choi, Tran et al [129] | 170 | 270 | USA | To describe patient characteristics associated with RCDI,  severe CDI disease, and treatment resistant RCDI. | Adult cancer patients who had both NAAT and EIA results positive for *C. difficile* were identified in our institutional laboratory database from January 2015 to May 2017 | Antibiotic use, Comorbidities, NSAID use, PPI use, Immunosuppressant use,  Chemotherapy, Organ transplantation,  Gastrointestinal GVHD,  Previous hospitalisation  Haematological malignancy, Mean length of CDI treatment, CDI treatment failure, Severe/fulminant CDI disease | *Clostridioides difficile* | Low | + | 38,6 | 13 | 7 | Authors concluded that the risk factors for RCDI they found in cancer patients were the same as one can otherwise find in non-cancer patients (previous antibiotic use, NSAID, comorbidities, and immunosuppressant use), except for the risk use of chemotherapeutic therapy. |
| 2020 | Alterations of the oral microbiome and cumulative carbapenem exposure are associated with *Stenotrophomonas maltophilia* infection in patients with acute myeloid leukemia receiving chemotherapy | Aitken, Sahasrabhojane, Kontoyiannis et al [85] | 90 | 8 | USA | To characterize cumulative antibiotic exposure and the relative abundance of *S*.  *maltophilia* in patients with AML in order to identify patients at increased risk for *S. maltophilia*  infections. | Patients with a new diagnosis of AML who were receiving remission-induction chemotherapy (RIC) between 9/2013 and 8/2015. | Ciprofloxacin, Levofloxacin, Meropenem, *S. maltophilia* oral abundance | *Stenotrophomonas maltophilia* | Low | - | 2,0 | 8 | 4 | Authors found that the abundance of *S. maltophilia* in the oral microbiome, in addition to fluoroquinolone use, is associated with *S. maltophilia* infection. |
| 2016 | Results of Four-Year Rectal Vancomycin-Resistant Enterococci Surveillance in a Pediatric Hematology-Oncology Ward: From Colonization to Infection | Akturk, Sutcu, Somer et al [147] | 72 | 7 | Turkey | To investigate the clinical impact of vancomycin resistant  enterococci (VRE) colonization in patients with hematologic  malignancies and associated risk factors | All patients admitted to the paediatric haematology/oncology ward with a documented Vancomycin-resistant enterococci colonisation 48-72 h after admission. | Severe neutropenia (<100/mm3), Previous bacteremia with another pathogen | Vancomycin-resistant enterococci | Medium | + | 3,5 | 10 | 2 | A brief report investigating the risk factors for progression from vancomycin-resistant enterococci colonisation to infection, which they find is associated with severe neutropenia and previous bacteraemia. |
| 2017 | A multicenter, retrospective, case- cohort study of the epidemiology and risk factors for *Clostridium difficile* infection among cord blood transplant recipients | Alonso, Braun, Patel et al [69] | 226 | 226 | USA | To retrospectively evaluate the local  epidemiology of CDI across three institutions, to describe novel  risk-factors  for development of CDI in UCBT recipients in the first  100 days after UCBT, and to describe the clinical spectrum of CDI in  this population so as to better determine whether infection is linked to  adverse secondary clinical outcomes | Adult Umbilical cord blood transplant (UCBT recipients at the three Boston centres from January 1, 2003 to December 31, 2012 | Age, BMI, Conditioning regimen, Antipseudomonal penicillin, Bacterial infection post- UCBT | *Clostridioides difficile* | Low | + | 45,2 | 21 | 5 | Authors identified having had a bacterial infection post-transplant as a risk factor of CDI, without considering the treatment of the bacterial infection. |
| 2016 | Retrospective analysis of the microbiological spectrum of pneumonia in Turkish patients with lung cancer | Avcı, Hartavi, Kaçan et al [43] | 119 | 22 | Turkey | To investigate the  potential correlates of pneumonia in patients with lung tumours | Patients with lung cancer and pneumonia between 2010 and 2011 | Male sex, Age, Diabetes mellitus, COPD, Histology, Tumour stage, Tumour localisation, Surgical treatment, Pleural effusion, Hospital-acquired pneumonia, Chemotherapy dose modifications, Respiratory failure, Febrile neutropaenia, Development of pneumonia within the first 10 days | *Aspergillus fumigatus* | Medium | - | 1,6 | 14 | 14 | Authors found that febrile neutropenia and pneumonia within the first 10 days of admission may increase the risk of *A. fumigatus* pneumonia. |
| 2020 | Use of carbapenems and glycopeptides increases risk for *Clostridioides difficile* infections in acute myeloid leukemia patients undergoing intensive induction chemotherapy | Ballo, Kreisel, Eladly et al [70] | 415 | 37 | Germany | To analyze the  incidence of CDI at the University Hospital Frankfurt and its  impact on the clinical course of induction chemotherapy and  (b) to identify factors associated with CDI | All patients with AML who underwent intensive induction chemotherapy between 2007 and 2019. | Cumulative glycopeptide exposure | *Clostridioides difficile* | Low | + | 7,4 | 8 | 5 | Authors found an association between cumulative exposure to some antibiotics and *C. difficile* infection |
| 2021 | Bacterial bile duct colonization in perihilar cholangiocarcinoma and its clinical significance | Bednarsch, Czigany, Heij et al [22] | 95 | 52 | Germany | To investigate bacterial bile duct colonization in a large European cohort of pCCA  patients and determine its impact on the postoperative outcome focusing on abdominal infections | All surgically treated patients with localised pCCA with available information on bile duct colonisation from intraoperative sampling | Portal vein embolization, Susceptibility to applied perioperative antibiotics | 47 species identified, top 5 most common: *Enterococcus faecalis*, *Enterococcus faecium, Enterobacter cloacae, Escherichia coli, Klebsiella pneumoniae* | Medium | + | 26,0 | 26 | 2 | Authors found that portal vein embolization and susceptibility to perioperative antibiotics is associated with postoperative abdominal infections after excluding a number of variables |
| 2016 | Epidemiology of *Clostridium difficile* infection in hospitalized oncology patients | Chang, Dembry, Banach et al [130] | 229 | 77 | USA | To identify demographic and  clinical risk factors associated with CDI among oncology inpatients | Adult patients admitted to oncology units, those with *C. difficile* infection were cases. | Age, Prior history of *Clostridioides difficile*, Blood transfusion, β-lactam/β-lactamase inhibitor, Cephalosporin, Metronidazole | *Clostridioides difficile* | Medium | + | 12,8 | 32 | 6 | Authors found a very strong association between a history of *C. difficile infection* and current *C. difficile* infection, in addition to a selection of antibiotics. |
| 2017 | Risk Factors for Hospital-acquired *Clostridium difficile* Infection Among Pediatric Patients With Cancer | Daida, Yoshihara, Inai et al [131] | 145 | 51 | Japan | To provide a descriptive account of  hospital-acquired CDI and its recurrence rate in a general  pediatrics ward, as well as to identify major risk factors for  this infection in the hospital setting | Paediatric patients with cancer admitted to the hospital between July 2003 and September 2012, CDI cases matched to controls | Age, Male (vs. female), Same ward, Duration of neutropenia | *Clostridioides difficile* | Low | + | 10,2 | 9 | 5 | Antibiotics use is omitted from the final model due to collinearity with duration of neutropenia, which is associated with an increased odds of CDI |
| 2015 | Infection with multidrug-resistant gram-negative bacteria in a pediatric oncology intensive care unit: risk factors and outcomes | de Oliveira Costa, Atta, da Silva [120] | 76 | 47 | Brazil | To evaluate the risk factors and outcomes associated with MDR-GNB infections in children with cancer and/or hematologic diseases | Infection episodes related to GNB that occurred between January 1, 2009 and December 31, 2012 in PICU patients, aged between 0 months and 18 years, who were hospitalised for more than 24 h in a tertiary oncology public hospital. | Healthcare-associated infection, Hematologic diseases, Length of neutropenia ≥3 days, Length of ICU stay >3 days, Previous antibiotic therapy | Multidrug-resistant Gram-negative bacteria | Medium | + | 9,4 | 14 | 5 | The authors found that there is a higher odds of a MDR-GNB infection compared to a non-MDR-GNB infection if the infection was healthcare-associated and the patient had a haematological cancer |
| 2018 | Increasing rates of *Acinetobacter baumannii* infection and resistance in an oncology department | Fan, Wang, Wang et al [140] | 6154 | 60 | China | To determine the relationship between *A.*  *baumannii* infections in patients and antibiotic use  as well as other risk factors | Cancer patients with nosocomial infections in a general teaching hospital from January 2010 to December 2015 | Gender, Age, Hematologic malignancy, Radiation, Hospitalisation time, Operation, Prior antibiotic use | *Acinetobacter baumannii* | Medium | - | 8,6 | 7 | 7 | Authors found that there is an association between prior antibiotic use and *A. baumannii* infection |
| 2018 | Clinical Outcomes Associated With Linezolid Resistance in Leukemia Patients With Linezolid-Resistant *Staphylococcus epidermidis* Bacteremia | Folan, Marx, Tverdek et al [108] | 82 | 33 | USA | To test  the hypotheses that LRSE bacteremia empirically treated with  linezolid would have worse clinical outcomes in comparison  with patients infected by linezolid-sensitive strains | All adult leukaemia patients with *S. epidermidis* bacteremia treated with empiric linezolid between 2012 and 2015. | Linezolid-resistant *S. epidermidis*, Age, AML, Prior HSCT, Presence of CVC | *Staphylococcus epidermidis*, linezolid-resistance | Low | + | 8,3 | 12 | 4 | The infection outcome was persistent *S. epidermidis* bacteraemia, which the authors found was more common in patients that were infected by linezolid-resistant bacteria |
| 2019 | Decrease in vancomycin-resistant Enterococcus colonization associated with a reduction in carbapenem use as empiric therapy for febrile neutropenia in patients with acute leukemia | Ford, Coombs, Stofer et al [89] | 342 | 79 | USA | The authors report the results of [changing empirical antibiotics] on the incidence of hospital acquired  VRE colonization. They also investigated the effects on  BSI rates, the gastrointestinal (GI) microbiome, dominant VRE  strains on the unit, and patient outcomes | Patients admitted with newly diagnosed acute myelogenous leukaemia (AML), acute lymphoblastic leukaemia(ALL), biphenotypic leukaemia (BPL), or chronic myelogenous leukaemia in blast phase (CML-BP) between September 2011 and August 2017 | Age, Days of empiric antibiotics, LOS, Cycling vs carbapenem period, Days of carbapenem, Days of cefepime or piperacillin/tazobactam | Vancomycin-resistant enterococci | Low | - | 13,2 | 6 | 6 | The infection outcome is VRE colonisation. The authors found that after the hospital changed policies to use less carbapenems, the VRE colonisation rates declined. |
| 2015 | Frequency, Risk Factors, and Outcomes of Vancomycin-Resistant Enterococcus Colonization and Infection in Patients with Newly Diagnosed Acute Leukemia: Different Patterns in Patients with Acute Myelogenous and Acute Lymphoblastic Leukemia | Ford, Lopansri, Haydoura et al [90] | 214 | 15 | USA | To describe the  frequency of, risk factors for, and outcomes associated with  VRE colonization and BSI in patients admitted with newly  diagnosed acute leukemia | Patients with newly diagnosed acute leukaemia between 2006 and 2012 | Severe neutropenia, Number of stools/day | Vancomycin-resistant enterococci | Low | + | 7,5 | 7 | 2 | Data extracted from the model with bloodstream infection as the infection outcome. Authors find that such infections are associated with prolonged neutropenia and diarrhoea. |
| 2018 | *Clostridioides difficile* colonization and infection in patients with newly diagnosed acute leukemia: Incidence, risk factors, and patient outcomes | Ford, Lopansri, Webb et al [71] | 509 | 31 | USA | The authors describe a  single-center experience with a large consecutive population of  patients admitted with newly diagnosed AL | Patients with AL admitted between 2006 and 2017 | Age, Gender female, Diabetes, Hospitalisation prior 2 mo, Antibiotics prior 3 mo, Karnofsky performance Status, Admit serum albumin, Severe neutropenia, Antibiotics (no.), Antibiotics (d) | *Clostridioides difficile* | Low | - | 3,1 | 10 | 10 | CDI during hospitalisation was found in 31 patients - more often in those who had recently been hospitalised or had recently been put on antibiotics |
| 2016 | Risk factors for *Clostridium difficile* infection in hemato-oncological patients: A case control study in 144 patients | Fuereder, Koni, Gleiss et al [132] | 288 | 144 | Austria | To to investigate risk factors for CDI in a large cohort of hemato-oncological  patients with microbiologically confirmed CDI | Patients that had a histologically or cytologically confirmed hemato-oncological disease diagnosed between 1st January 2004 and 31st December 2014. Cases were patients that had received chemotherapy during the course of their disease and suffered from CDI positive diarrhoea, controls were those with CDI negative diarrhoea. | Intervall chemotherapy to diarrhoea, Antibiotic therapy within 30 days | *Clostridioides difficile* | Medium | - | 72,0 | 2 | 2 | The authors found that chemotherapy is not associated with CDI positive diarrhoea, but that antimicrobial therapy is. |
| 2017 | Consequences of Increases in Antibiotic Resistance Pattern on Outcome of Pancreatic Resection for Cancer | Gianotti, Tamini, Gavazzi et al [23] | 517 | 99 | Italy | To explore the  potential correlation between the occurrence of multidrug-susceptible,  multidrug-resistant, extensively drug-resistant, and  pan-drug-resistant bacterial infections developing during the  postoperative course and the rate of non-infectious complications  occurring after the onset of infection | Adult patients who underwent elective pancreatic resections for cancer from January 1, 2013 to December 31, 2015, with an available sample of biological fluid. | Type of operation (pancreatoduodenectomy vs. others), Operation time, Estimated blood loss, Preoperative biliary stenting (yes vs. no) | Several bacteria, tested for resistance towards several antibiotics | Low | - | 24,8 | 4 | 4 | The authors found that there is an association between preoperative biliary stenting and multidrug or extensively drug resistant infectious complications |
| 2019 | Colonization by fecal extended-spectrum b-lactamase-producing *Enterobacteriaceae* and surgical site infections in patients with cancer undergoing gastrointestinal and gynecologic surgery | Golzarri, Silva-Sanchez, Cornejo-Juarez et al [38] | 171 | 25 | Mexico | To describe the  prevalence of ESBL-PE fecal carriage in cancer patients prior to surgery  (at time of admission) and during their hospital stay, and  whether carriers have a major risk of infectious complications | Adult patients, with gynaecological or gastrointestinal malignancies, who were admitted to the hospital for elective abdominal and pelvic surgical procedures from September 2014 to December 2015 | ESBL-PE. Adjusted for the following confounders: age, sex, albumin, and type of malignancy. | Extended-spectrum beta-lactamase-producing *Enterobacteriaceae* | Low | + (with fixed set of confounders) | 3,6 | 13 | 7 | The risk ratio of several infectious outcomes in carriage ESBL-producing Enterobacteriaceae vs non-carriers are modelled, after adjusting for confounders. The authors found that the risk of surgical site infection and blood stream infection is increased by such carriage, but not pneumonia and urinary tract infection. |
| 2020 | Clinical Predictive Model of Multidrug Resistance in Neutropenic Cancer Patients with Bloodstream Infection Due to *Pseudomonas aeruginosa* | Gudiol, Albasanz-Puig, Laporte-Amargos et al [144] | 1217 | 309 | 34 centres in 12 countries | To assess the rate and evolution of multidrug  resistance among *P. aeruginosa* isolates causing BSI in neutropenic cancer patients over  recent years and to develop a clinical prediction model to estimate the probability of  multidrug resistance acquisition in this population | Adult neutropenic onco haematological patients diagnosed with at least one episode of *P. aeruginosa* BSI from 1 January 2006 to 31 May 2018. | Prior piperacillin-tazobactam therapy (within 1 mo), Prior fluoroquinolone prophylaxis (within 1 mo), Urinary catheter, Prior antipseudomonal carbapenem therapy (within 1 mo), Haematological disease | *Pseudomonas aeruginosa*, multidrug resistant | Low | - | 51,5 | 32 | 6 | The authors constructed a clinical prediction model to predict resistance in P. aeruginosa bloodstream infections in onco haematological patients, achieving an area under the curve of 0.72 (0.63, 0.80) in internal validation. A risk score calculator of this model is available on https://ubidi.shinyapps.io/ironic/. |
| 2017 | Risks factors and outcomes of *Clostridium difficile* infection in patients with cancer: a matched case-control study | Hebbard, Slavin, Reed et al [133] | 200 | 50 | Australia | (i) To describe the  clinical characteristics of cancer patients acquiring CDI, (ii) To  determine risk factors for CDI in cancer patients, and (iii) To  evaluate outcomes of CDI in cancer patients | Patients with CDI at a cancer centre from May 2013 to May 2015 | Administration of chemotherapy, Gastro-intestinal/abdominal surgery, Proton pump inhibitors, Antibiotic days of therapy | *Clostridioides difficile* | Low | + | 12,5 | 27 | 4 | The authors did not find that the administration of any antibiotics is associated with a *C. difficile* infection, but that chemotherapy is. |
| 2016 | Vancomycin- resistant *Enterococcus* colonization and bloodstream infection: prevalence, risk factors, and the impact on early outcomes after allogeneic hematopoietic cell transplantation in patients with acute myeloid leukemia | Hefazi, Damlaj, Alkhateeb et al [92] | 203 | 12 | USA | To investigate the impact of VRE colonization  and BSI on early transplant outcomes in a relatively homogenous  cohort of patients with acute myeloid leukemia (AML)  undergoing allogeneic HCT in a single institution with strict infection  control protocols; To identify the risk factors  for VRE BSI early after HCT, and to determine whether VRE BSI  was an independent prognosticator of outcomes in this high-risk  population | All consecutive patients who underwent their first allogeneic HCT for AML at ta clinic between April 2004 and December 2014 | Age (≥60 years vs <60), HCI-CI (≥3 vs 0- 2), KPS (>80 vs ≤80), Conditioning (RIC vs MAC), VRE colonisation (within 30 days of HCT) | Vancomycin-resistant enterococci | Low | + | 2,4 | 10 | 5 | The authors found that there is an association between VRE colonisation and VRE bloodstream infection |
| 2017 | Vancomycin-resistant enterococci in acute myeloid leukemia and myelodysplastic syndrome patients undergoing induction chemotherapy with idarubicin and cytarabine | Heisel, Sutton, Mascara et al [93] | 229 | 134 | USA | To determine the factors associated with  VRE colonization/infection in a uniform cohort of AML  and MDS patients undergoing intensive induction  chemotherapy in our institution | Adult patients who received intensive induction chemotherapy with the standard 7+3 regimen of cytarabine and idarubicin for newly diagnosed AML or MDS from January 2012 to December 2015 | Gender (male), Vancomycin IV, Cephalosporin | Vancomycin-resistant enterococci | Medium | + | 44,7 | 14 | 3 | The authors found an association between both cephalosporins and intravenous vancomycin, and a VRE infection |
| 2017 | Changes in In Vitro Susceptibility Patterns of Aspergillus to Triazoles and Correlation With Aspergillosis Outcome in a Tertiary Care Cancer Center, 1999–2015 | Heo, Tatara, Jimenez-Ortigosa et al [105] | 107 | 19 | USA | To determine if any changes in susceptibility  were species-, triazole-, and/or host-dependent, and their  clinical implications | A case-control study of patients with haematological malignancy comparing azole-resistant strains is nested within a cohort of all Aspergillus species clinical isolates recovered from respiratory sources in patients treated at a cancer centre between January 1999 and December 2015 | Asian race, Culture specimen–BAL fluid, Previous azole exposure history | Four *Aspergillus* species (fumigatus, flavus, terreus, niger), azole-resistance | Low | + | 6,3 | 58 | 3 | The authors concluded that there are three independent risk factors of invasive pulmonary aspergillosis caused by Aspergilli with a non-wild type azole MIC - asian race, bronchoalveolar lavage fluid culture and a history of azole exposure. There is also a univariate analysis of mortality in the article. |
| 2017 | Daptomycin nonsusceptible vancomycin resistant Enterococcus bloodstream infections in patients with hematological malignancies: risk factors and outcomes | Herc, Kauffman, Marini et al [91] | 60 | 20 | USA | An increase in bloodstream infections  due to daptomycin non susceptible VRE among  patients in the Hematology and HCT Units, prompted  the authors to identify risk  factors, assess treatment strategies and characterize  outcomes in these populations | Adult patients treated on an inpatient haematology service from January 2011 to December 2015 with a haematological malignancy and a blood culture positive for an enterococcus species. | Congestive heart failure, Typhilitis, Gastrointestinal bleeding, Daptomycin, Cefepime received within 90 days, Cumulative Daptomycin days within 90 days, Cumulative Fluoroquinolone days within 90 days, Prior enterococcal infection, Hospital LOS prior to positive culture,, Stage of malignancy, Number induction chemotherapy regimens, Clofarabine within 30 days | *Enterococcus*, vancomycin and daptomycin resistance | Medium | + | 1,4 | 46 | 14 | The authors found that the only risk factor associated with a daptomycin-resistant VRE infection is daptomycin exposure within 90 days |
| 2019 | A RESEARCH STUDY ON BACTERAEMIA PRODUCED THROUGH ESCHERICHIA COLI IN TUMOR PATIENTS AT THE SPECIFIC CENTER IN OUR COUNTRY | Khanzada, Zianab, Samreen [153] | 229 | 99 | Pakistan | To examine anti-microbial vulnerability designs of *E. coli* bacteremia amongst tumor patients, also to measure danger issues also consequences of MDR *E. coli* bacteremia | Cancer patients with *E. coli* bacteremia from October 2014 to September 2015 | Man sex, Age fewer than 19 years, Haematological distortion, Hospitalization inside 1 month before contagion, ICU admission, Carlson score, Dominant intravenous tube use, Previous chemotherapy inside 1 month, Previous surgery inside 1 month, Preceding fallout inside 1 month, ANC less than 110 cells/mm3, Usage of quinolones inside 95 days of catalogue sample, Usage of third group cephalosporins inside 95 days of index sample, Usage of Piperacilin/Tazobactam within 95 days of index sampling, Usage of carbapenems inside 95 days of index sample | *Escherichia coli*, multidrug-resistant | High | - | 7,1 | 14 | 14 | The authors find that multidrug-resistant *E. coli* bacteraemia was associated with young age, central venous catheter, and piperacillin/tazobactam exposure within 95 days of infection |
| 2018 | Clinical predictors of *Stenotrophomonas maltophilia* bacteremia in adult patients with hematologic malignancy | Kim, Cho, Kang et al [86] | 236 | 118 | South Korea | To identify clinical predictors of  *S. maltophilia* bacteremia in adult patients with hematologic  malignancy compared with other GNB | Cases were *S. maltophilia* bacteremia in adult patients with hematologic malignancy. Controls were other Gram-negative (*Escherichia coli,* *Klebsiella* species, *Acinetobacter* species, *Pseudomonas* species, and Enterobacter species) bacteraemias. | Polymicrobial infection, Previous *S. maltophilia* isolation, Breakthrough infection during carbapenem therapy, No. of previous antibiotic use ≥3, Previous TMP/SMX use | *Stenotrophomonas maltophilia* | Medium | + | 23,6 | 18 | 5 | The authors concluded that all independent variables included in the final multivariable model were associated with *S. maltophilia* bacteraemia. |
| 2019 | Risk factors for extended‑spectrum beta‑lactamase‑producing Enterobacteriaceae infection causing septic shock in cancer patients with chemotherapy‑induced febrile neutropenia | Kim, Jung, Kang et al [123] | 179 | 32 | South Korea | To identify risk factors suggestive  of ESBL-PE infection in septic shock patients with  chemotherapy-induced FN to guide appropriate antibiotic  therapy. | Adult (≥ 18 years) patients with chemotherapy-induced febrile neutropenia admitted to an emergency department with septic shock | Profound neutropenia | Enterobacteriaceae, extended-spectrum beta-lactamase | Low | - | 6,4 | 5 | 5 | After adjustment, the authors found that only profound neutropenia was associated with ESBL-PE infection |
| 2021 | Antibiotic Prophylaxis or Granulocyte-Colony Stimulating Factor Support in Multiple Myeloma Patients Undergoing Autologous Stem Cell Transplantation | Klein, Sauer, Klein et al [94] | 298 | 25 | Germany | To compare antibiotic prophylaxis with  ciprofloxacin or cotrimoxazole versus G-CSF support, with respect to blood count recovery,  infectious complications, and emerging MDR bacteria in patients with MM, undergoing  HDT/ASCT | Multiple myeloma inpatients who received high-dose therapy with melphalan followed by autologous stem cell transplantation at a hospital between March 2016 and July 201 | Antibiotic prophylaxis (vs. G-CSF support), No prophylaxis (vs. G-CSF support), Age (per ten years), ASCT at relapse (vs. first-line treatment), ≥VGPR before ASCT (vs. ≤PR), Stem cell amount ≥2.5 * (vs. <2.5) | Enterococcus faecium, vancomycin-resistant | Medium | - | 4,2 | 6 | 6 | The article includes analyses of several microbes, but the assessed model investigates the association between the listed risk factors and VRE detection. Authors concluded that Granulocyte-Colony Stimulating Factor instead of antibiotic prophylaxis is associated with a fewer detected VRE cases. |
| 2015 | Clinical features and risk factors for development of Bacillus bacteremia among adult patients with cancer: a case-control study | Ko, Kang, Lee et al [164] | 258 | 86 | South Korea | To  evaluate the risk factors related to the development of Bacillus  bacteremia and its prognosis in cancer patients | Cases were adult patients with cancer who were diagnosed with Bacillus bacteremia during the period of January 1995 through December 2012. Controls were patients with bacteraemia due to other pathogens. | Hospital stay of longer than 14 days, History of HSCT, Presence of a CVC, Hematologic malignancy, Diarrhoea at presentation, Prior use of extended-spectrum cephalosporin, Prior use of glycopeptides | *Bacillus spp.* | Low | - | 9,6 | 9 | 9 | The authors found that in addition to a central venous catheter, the use of cephalosporins within a month of admission was associated with Bacillus bacteraemia |
| 2016 | Risk factors for the development of *Clostridium difficile* infection in adult allogeneic hematopoietic stem cell transplant recipients: A single- center study in Québec, Canada | Lavallee, Labbe, Talbot et al [72] | 188 | 65 | Canada | To describe the risk factors  associated with CDI during the first year following allo-HSCT,  including  those specifically associated with early and late CDI | Adults allo−HSCT recipients. Cases were CDI positive, controls were CDI negative. | Mucositis, CMV reactivation, Herpesviridae reactivation other than CMV, Antibiotic use within 30 days before transplantation (No antibiotic, Any antibiotic except TMP-SMX, TMP-SMX) | *Clostridioides difficile* | Low | + | 13,0 | 30 | 5 | The authors concluded that reactivation of CMV and other Herpesviridae was associated with CDI. There is also a multivariable model stratified on the time of CDI positivity, which shows that high-risk antibiotics were associated with CDI late in the treatment. |
| 2015 | Risk factors for acquisition of multidrug-resistant bacteria in patients with anastomotic leakage after colorectal cancer surgery | Lee, Ryu, Chung et al [24] | 143 | 46 | South Korea | To identify independent risk factors for the  acquisition of MDR pathogens at the time of diagnosis of  anastomotic leakage after colorectal cancer surgery. We also  evaluated risk factors for MDR bacterial acquisition in the  early phase of leakage to improve early antibiotic therapy after  diagnosis of leakage. We compared the outcomes of patients  with MDR pathogens to patients without MDR pathogens | Patients with colorectal cancer that underwent surgery and had anastomotic leakage and a subsequent positive blood or intra abdominal fluid culture from January 2009 to April 2013 | Diabetes mellitus, The total length of antibiotic administration for more than 5 days before diagnosis of anastomosis site leakage. Adjustment variables indeterminable. | Several bacteria and fungi, tested for resistance towards several antimicrobials | Medium | + | Indeterminable | Indeterminable | Indeterminable | The authors found that compared to non-MDR pathogens, diabetes mellitus and the total length of antibiotic administration for more than 5 days before diagnosis of anastomosis site leakage were associated with acquisition of a MDR pathogen. |
| 2017 | Post-operative MRSA infections in head and neck surgery | Lin, Melki, Lisgaris et al [42] | 113 | 24 | USA | To identify patients with MRSA SSIs  Following any head and neck procedure | Patients who were >18 years of age and underwent major head and/or neck surgery between January 1, 2008 and January 1, 2014, and were found to have a documented surgical site infection during their postoperative hospitalisation | Indeterminable | *Staphylococcus aureus*, methicillin-resistance | Medium | - | Indeterminable | Indeterminable | Indeterminable | The authors describe a multivariable model in the methods chapter, but we were unable to locate a regression table. Nonetheless, the authors conclude that hospital exposure prior to the procedure, a history of chemotherapy, or immunosuppression were risk factors for developing a MRSA SSI. |
| 2015 | Risk for *Clostridium difficile* infection after radical cystectomy for bladder cancer: Analysis of a contemporary series | Liu, Shatagopam, Monn et al [39] | 552 | 49 | USA | To determine the incidence of CDI in a contemporary cohort undergoing RC at a high-volume tertiary referral center and to identify the perioperative risk factors for development CDI in patients undergoing RC. | Patients undergoing radical cystectomy between January 2010 and December 2013 for bladder cancer | Diversion type (Ileal conduit, Indiana pouch, Orthotopic neobladder), Preoperative haemoglobin, Preoperative antacid use, Days of antibiotics received (1 day or less, 2–7 days, Greater than 7 days) | *Clostridioides difficile* | Low | + | 8,2 | 22 | 6 | The authors found that chronic antacid therapy and long duration of antibiotic exposure in the perioperative setting were associated with a *C. difficile* infection in these patients |
| 2018 | Mixed mold pulmonary infections in haematological cancer patients in a tertiary care cancer centre | Magira, Jiang, Economides et al [106] | 54 | 27 | USA | To evaluate  the incidence, clinical and mycological characteristics, risk factors,  and outcome of patients with haematological cancer who  developed MMPIs. | Cases were adult (>18 years old) patients with haematological malignancy who had proven or probable pulmonary infection with >1 mould grown concurrently in sputum or bronchoalveolar lavage (BAL) cultures, controls were patients with haematological cancer (from the same time period) with IPA caused by Aspergillus fumigatus. | Significant use of corticosteroids, culture from sputum specimens | Mixed moulds, *Aspergillus fumigatus* | Medium | + | Indeterminable | 29 | Indeterminable | We were unable to locate a printed regression table, but the authors concluded that there is an association between use of corticosteroids and sputum samples, and mixed moulds instead of *A. fumigatus* infection |
| 2015 | Risk factors for piperacillin/tazobactam-resistant Gram-negative infection in hematology/oncology patients with febrile neutropenia | Marini, Hough, Gregg et al [121] | 171 | 25 | USA | To determine risk factors for PTZresistant (PTZ-R) isolates in hematology/oncology patients  with FN | Adult haematology/oncology patients with a diagnosis of FN and any culture positive for a Gram-negative rod from November 2007 to November 2013 | Demographic model: Unit ICU, Source of positive culture (Respiratory). Resistance model: History of PTZ-R, Antibiotic use previous 90 days (Antibiotic therapy >14 days), Specific antibiotics previous 90 days (PTZ, Vancomycin), LOS >14 days prior to culture, Healthcare exposures previous 90 days (ICU admission), Transfer from outside hospital, Urinary catheter at culture. Haematology/oncology model: Stage of disease (Hematologic malignancy–newly diagnosed, Hematologic malignancy–continuation/consolidation), Oncology treatment previous 90 days (Rituximab, Clofarabine). | Gram-negative bacilli, Piperacillin/tazobactam resistance | Medium | + (collinear variables removed) | Indeterminable | 69 | Indeterminable | There are three models - one with demographic variables, one with resistance variables, and one with haematology/oncology-specific variables. A final model with variables from all these models are described and the area under the curve of this model is reported, but a regression table is not printed. The authors concluded that prolonged antibiotic use and critical illness increase the risk of piperacillin/tazobactam resistance. |
| 2020 | Development and validation of a scoring system for predicting cancer patients at risk of extended-spectrum b-lactamase-producing Enterobacteriaceae infections | Martínez-Valencia, Martínez, Ayala et al [128] | 710 | 265 | Colombia | To develop and validate a  reliable and easy-to-use clinical scoring system to identify  patients with solid or hematologic malignancies with a  high risk of ESBL-PE infections at the National Cancer Institute of Colombia | Cancer patients with documented microbiological isolation during hospitalisation at a national cancer institute | Prolonged hospitalisation (≥7 days), Hospitalisation during previous year, Immunosuppressive therapy, Neutropenia, Beta-lactams during the previous month, Invasive devices at the time of culture, Neoplasia in remission, No chemotherapy | *Enterobacteriaceae*, ESBL | Low | + | 33,1 | 39 | 8 | The authors did develop a clinical prediction model, which is validated in a separate cohort and assessed with an area under the curve. The variable selection in the prediction model is based on the statistical significance of the candidate predictors in a stepwise regression. The authors translated the model into a scoring system, with prolonged hospitalisation and previous hospitalisation the last year as the highest scores. |
| 2019 | Oral Vancomycin Prophylaxis as Secondary Prevention Against *Clostridium difficile* Infection in the Hematopoietic Stem Cell Transplantation and Hematologic Malignancy Population | Morrisette, Van Matre, Miller et al [73] | 50 | 11 | USA | To evaluate the effectiveness and  safety of an oral vancomycin prophylaxis (OVP) protocol for secondary prevention of CDI in a retrospective cohort of  adult autologous/allogeneic HSCT recipients and patients with Hematologic Malignancy who did not undergo HSCT with a first CDI episode treated with concomitant broad-spectrum antibiotics (BSA) | Patients aged >18 years with a history of autologous or allogeneic HSCT and patients with HM who did not undergo HSCT, who have been treated for the initial episode of CDI first with planned oral vancomycin monotherapy and must have been receiving a broad-spectrum antibiotic at the time of CDI diagnosis and/or during the course of CDI treatment/prophylaxis. | Oral vancomycin prophylaxis, Outpatient CDI diagnosis. | *Clostridioides difficile* | Low | + | 5,5 | Indeterminable | 2 | The infection outcome here is recurrent CDI infection, which the authors found was associated with no oral vancomycin prophylaxis and an inpatient CDI diagnosis. |
| 2019 | Antimicrobial de-escalation in adult hematopoietic cell transplantation recipients with febrile neutropenia of unknown origin | Petteys, Kachur, Pillinger et al [74] | 107 | 3 | USA | To evaluate the outcomes  associated with early de-escalation of Broad Spectrum Antibiotics prior to  hematopoietic recovery in autologous and allogeneic  HCT recipients with FN of unknown origin. | Adult HCT recipients with FN at an academic teaching hospital between March 2014 and April 2018. | Early de-escalation vs hematopoietic recovery de-escalation, Gender, Race, Age | *Clostridioides difficile* | Medium | - | 0,8 | 4 | 4 | The authors did not find that the early de-escalation of broad-spectrum antibiotics was associated with a Clostridioides difficile infection |
| 2017 | Moxifloxacin versus levofloxacin or ciprofloxacin prophylaxisin acute myeloid leukemia patients receiving chemotherapy | Przybylski & Reeves [75] | 141 | 141 | USA | To provide  more insight into the use of moxifloxacin as a prophylactic antibiotic in immunocompromised patients with  prolonged neutropenia, specifically compared with  levofloxacin or ciprofloxacin | Adult patients admitted to the hospital with a diagnosis of AML who received induction or reinduction chemotherapy from January 2008 to June 2015 | Prophylactic antibiotherapy received, treatment antibiotic duration, mucositis, proton pump inhibitor, neutropenic days | *Clostridioides difficile* | Medium | - | 28,2 | Indeterminable | 5 | Authors found that treatment antibiotic duration was the only statistically significant association with with *C. difficile* infection |
| 2019 | Risk, Outcomes, and Trends of *Clostridium Difficile* Infection in Multiple Myeloma Patients from a Nationwide Analysis | Ran-Castillo, Oluwole, Abuaisha et al [76] | 114249 | 3549 | USA | To determine the risk of *Clostridium difficile* infection (CDI) in  hospitalization with multiple myeloma (MM), as well as its outcomes and trends, using a  nationally representative database | Patients with a diagnosis of MM between 2010 and September 2015 | Year (2010, 2011, 2012, 2013, 2014, 2015), Age categories (18-49, 50-59, 60-69, 70-79, ≥ 80), Race/Ethnicity (White, Black, Hispanic, Others), Female, Obesity, Peripheral vascular disease, Chronic renal disease, Atrial fibrillation, Stem cell transplantation, Neutropenia,  Chemotherapy, Inflammatory bowel disease, Congestive heart failure, Hypertension, Chronic liver failure, Elixhauser score (0, 1-3, ≥ 4), Hospital bed size (Small, Medium, Large), Hospital teaching status (Rural, Urban non-teaching, Urban teaching), Expected primary payer (Medicare, Medicaid, Private, Others), Median household income in quartile (1st, 2nd, 3rd, 4th). | *Clostridioides difficile* | Medium | - | 177,5 | 20 | 20 | The authors balanced multiple myeloma patients with non-multiple myeloma using propensity scores, then modelled the risk of CDI and of mortality given CDI. The unadjusted OR for mortality is also reported. They found several risk factors for CDI, e.g. neutropenia. |
| 2021 | Risk of Drug Resistance and Repeated Infection with *Klebsiella pneumoniae* and *Escherichia coli* in Intensive Care Unit Cancer Patients | Refay, Ahmed, ELzaher et al [124] | 107 | 38 | Egypt | To identify predictors of repeat infections of *Escherichia coli*  and *Klebsiella pneumoniae* and drug resistance in cancer patients admitted to the intensive care  unit (ICU) in Upper Egypt | Cancer patients who were treated at either the Paediatric Oncology or the Medical Oncology ICU with an initial infection or repeated infection with either *K. pneumoniae* or *E. coli* from February 2017 to May 2018 | Age at diagnosis, Type of Malignancy, Type of Organism, Total prior Hospital Stay, Total Prior Admission in ICU, Prior Mechanical Ventilation, Prior Urinary Catheterization, Peripheral or Central CVP Line, Infection | *Klebsiella pneumoniae* and *Escherichia coli*, tested for resistance towards several antibiotics | Low | - | 4,8 | 9 | 8 | The infection outcome is drug resistance in the previous infection. A ROC curve is printed to show predictive accuracy. The authors conclude that restricting unnecessary antibiotic prescriptions is important to prevent the emergence of multidrug-resistance in these infections |
| 2015 | Risk factors associated with *Clostridium difficile* infection in adult oncology patients | Rodriguez Garzotto, Merida Garcia, Munoz Unceta et al [40] | 225 | 39 | Spain | To better ascertain  the incidence, susceptibility, and risk factors for CDI in cancer  patients receiving chemotherapy at our hospital | Hospitalised cancer patient with diarrhoea between January 2009 through April 2013 | Gastrointestinal cancer, Breast cancer, Lung cancer, Age (>65), Antibiotic exposure, Corticosteroid use, PP inhibitor use | *Clostridioides difficile* | Medium | - | 9,8 | 4 | 4 | The authors stratified three tables based on cancer types and include 4 previously identified risk factors. They concluded that antibiotic exposure was associated with CDI infection |
| 2016 | Bacteremia due to carbapenem-resistant Enterobacteriaceae in neutropenic patients with hematologic malignancies | Satlin, Cohen, Ma et al [64] | 301 | 43 | USA | To determine the prevalence, risk factors, treatments, and outcomes  of bloodstream infections (BSIs) due to carbapenem-resistant *Enterobacteriaceae* (CRE) in  adult neutropenic patients with hematologic malignancies | Cases were adult neutropenic patients with haematological malignancies with carbapenem-resistant Enterobacteriaceae bacteraemias. Primary control group was patients with bacteraemias caused by pathogens other than carbapenem-resistant Gram-negative bacteria, and the secondary control group was patients with carbapenem-susceptible Gram-negative bacteria. | b-lactam/b-lactamase inhibitor within previous 30 days, Carbapenem within previous 30 days, Receiving TMP-SMX at BSI onset, Receiving glucocorticoids at BSI onset, Prior CRE at any site within the previous 90 days, ICU stay within previous 30 days, Receiving cephalosporin at BSI onset, Receiving fluoroquinolone at BSI onset, Renal disease | *Enterobacteriaceae*, carbapenem-resistance | Medium | + | 4,8 | 81 | 9 | The infection outcome here was bacteraemia. The authors find that previous CRE infection and antibiotic exposure was associated with CRE bacteraemia |
| 2017 | Risk factors and impact of *Clostridium difficile* recurrence on haematology patients | Scappaticci, Perissinotti, Nagel et al [77] | 100 | 41 | USA | To identify risk factors for recurrent CDI (rCDI) and to determine the impact of  rCDI on adult patients with a haematological malignancy | Adult (age >18 years) patients with an active haematological malignancy and CDI from June 2010 to December 2014 | Demographic and traditional model: Severe CDI, salvage lymphoma. Longitudinal model: Hospitalised (days), Antibiotic exposure (ceftriaxone, fluoroquinolones, piperacillin/tazobactam, other (daptomycin), other (vancomycin IV), number of antibiotic exposures), Haematological risk factors (neutropenia (days), chemotherapy exposure, decitabine, CHOP, salvage lymphoma) | *Clostridioides difficile* | Medium | + | 20,5 | 40 | 2 | The infection outcome is recurring CDI, and there is one model containing demographical and traditional risk factors, and one containing longitudinal risk factors. Data are extracted from the "traditional" model. The authors found that exposure to a number of antibiotics were associated with recurrent CDI. |
| 2019 | *Clostridium difficile* infection in cancer patients with hospital acquired diarrhea at the teaching hospitals in Iran: Multilocus sequence typing analysis (MLST) and Antimicrobial resistance pattern | Shoaei, Shojaei, Khorvash et al [134] | 67 | 19 | Iran | To investigate the phenotype and genotype characterization of *Clostridium*  *difficile* isolates among cancer patients with hospital-acquired diarrhea in 4 teaching hospitals in Isfahan,  Iran | Adult (>18 years old) cancer patients with presence of hospital-acquired diarrhea at major teaching hospitals between April 2015 and May 2017 | Chronic renal diseases, Gastroenteritis diseases, Previous surgery | *Clostridioides difficile* | Low | + | 6,3 | 14 | 3 | The authors found that patients with gastrointestinal diseases had an association with lower CDI incidence |
| 2015 | Development of a combination antibiogram for *Pseudomonas aeruginosa* bacteremia in an oncology population | Smith, Tajchman, Dee et al [145] | 123 | 36 | USA | To develop a combination  antibiogram to aid clinicians in the empiric management of presumed P. aeruginosa bacteremia in a  cancer population;  To identify risk factors for MDR *P. aeruginosa* bacteremia; To develop a combination antibiogram specifically for ICU and hematologic malignancy patients, and  to determine the impact of empiric antibiotics on survival to hospital discharge | *Pseudomonas aeruginosa* isolates from an oncology population at least 18 years of age who were hospitalised between 1 January 2012 and 31 December 2012 | Prophylactic antibiotic (None, Fluoroquinolone, Cefpodoxime, Amoxicillin-clavulanate), LOS prior to culture (<5 days, >5 days), IV antibiotics in previous 90 day, Hemodialysis in previous 30 day | *Pseudomonas aeruginosa,* multidrug resistant | Low | + | 9,0 | 5 | 4 | The authors did develop an antibiogram for multidrug-resistant P. aeruginosa. In multivariable analysis they found that such bacteraemias were associated with receiving IV antibiotics 90 days prior |
| 2019 | Significance of bile culture surveillance for postoperative management of pancreatoduodenectomy | Sugimachi, Iguchi, Mano et al [36] | 69 | 43 | Japan | To determine the significance of preoperative surveillance bile culture in perioperative management of pancreatoduodenectomy | Patients who underwent pancreatoduodenectomy at a single institute between the years 2014 and 2017 | Smoking history (+ vs -), Pancreatic texture (soft vs hard) | *Enterobacter spp., Staphylococcus aureus, Pseudomonas spp.,* multidrug-resistance | Medium | + | 21,5 | 8 | 2 | The authors do not find that having a multidrug-resistant bacteria in the preoperative bile culture was associated with surgical site infection following a pancreatoduodenectomy, and as such did not include it in the multivariable model |
| 2019 | Risk factors for recurrent percutaneous nephrostomy catheter-related infections | Szvalb, El Haddad, Rolston et al [25] | 81 | 10 | USA | To identify the risk factors leading to recurrent percutaneous nephrostomy related infections (PCNI) in cancer patient | Patients who had undergone an initial percutaneous nephrostomy catheter placement at the hospital between July 2014 and February 2017 | Concurrent antibiotics use for PCN infection, PCN exchange within 4 days of infection | Several bacteria | Medium | + | 5,0 | 10 | 2 | The infection outcome here is recurrent catheter-related infection. The authors did not find any association between intrinsically resistant pathogens and recurrent infections |
| 2019 | Oral Candida colonization in xerostomic postradiotherapy head and neck cancer patients | Tarapan, Matangkasombut, Trachootham et al [44] | 72 | 72 | Thailand | To evaluate oral colonization of Candida species, especially for non‐  albicans Candida species (NACS), in xerostomic postradiotherapy head and neck can  cer patients and risk factors affecting their colonization | Xerostomic post radiotherapy head and neck cancer patients, aged from 30 to 70 years old, who finished radiotherapy for at least 1 month and/or chemotherapy for at least 2 weeks,from 3 Thai institutes | Sex, denture, antifungals, objective dry mouth scores, NACS (?) | *C. albicans, Candida spp.* | Medium | + | 14,4 | 8 | 5 | Authors found that increased signs of dry mouth, female sex and dental prostheses may promote NACS colonization. |
| 2021 | *Clostridioides difficile* Infections in Inpatient Pediatric Oncology Patients: A Cohort Study Evaluating Risk Factors and Associated Outcomes | Willis, Huang, Elward et al [135] | 952 | 109 | USA | To evaluate the prevalence and  risk factors for CDI in inpatient pediatric oncology patients  and to assess their associated outcomes compared to pediatric  oncology patients without CDI at a large academic pediatric  hospital. | Paediatric patient with malignancies admitted at St Louis hospital between July 2009 and February 2018 | Model 1 Risk for CDI: Length of stay before CDI, Leukaemia, Lymphoma, Autologous transplant, Allogeneic transplant, CDI exposure, Antibiotic exposure prior to CDI ; Model 2 outcomes linked to CDI : LOS, Chemotherapy delay OR and mean difference. | *Clostridioides difficile* | Low | + | 15,6 | 16 | 7 | Leukaemia, Lymphoma, autologous transplant, antibiotic exposure, CDI exposure were associated with CDI. The authors also showed that LOS and chemotherapy delay were outcomes linked to CDI. |
| 2019 | Intensity of Therapy for Malignancy and Risk for Recurrent and Complicated *Clostridium difficile* Infection in Children | Willis, Nicholson, Esbenshade et al [136] | 122 | 122 | USA | We sought to determine whether intensity of cancer therapy was associated with risk of  recurrent CDI and other adverse outcomes | Children >12 months and <18 years of age undergoing treatment for cancer and diagnosed with CDI between August 1st, 2008 and May 1st, 2015 | Level 2 of patient’s Intensity of Treatment Rating Scale, level 3 with HCST, level 3 without HCST and the HCST status | *Clostridioides difficile* | Low | - | 30,5 | Indeterminable | 4 | The authors concluded that they did not manage to find which paediatric oncology patients were at increased risk of CDI recurrence. |
| 2015 | Monitoring antibiotic-resistant enterobacteria  faecal levels is helpful in predicting antibiotic  susceptibility of bacteraemia isolates in patients  with haematological malignancies | Woerther, Micol, Angebault et al [65] | 104 | 16 | France | To determine the predictive values of  testing patients for BSBL-RE carriage in forecasting the susceptibility to BSBLs of the bacterial strains responsible for  EB-BSI and the impact of semiquantitative assessment of  BSBL-RE carriage levels on the performances of the test | Patient with neutropenia, haematological malignancies and EB-BSI from a probable intestinal origin | Death during neutropenia, non beta lactam antibiotic previous exposure, Carbapenem previous exposure, empirical treatment of EB-BSI with carbapenems, empirical treatment with antibiotics combination, presence of BSBL-Resistant EnteroBacteria, ESBL-EB, and non E. coli EB in blood cultures | Enterobacteria, BSBL or ESBL | Low | + | 2,0 | 20 | 8 | The authors showed that to have a BSBL-RE carriage increased risk to have BSBL-RE in blood cultures. |
| 2020 | Clinical Characteristics and Risk Factors for Bloodstream Infection Due to Carbapenem-Resistant *Klebsiella pneumoniae* (CRKP) in Patients with Hematologic Malignancies | Zhang, Wang, Hu et al [99] | 734 | 22 | China | To examine the clinical characteristics and risk factors for bloodstream infection (BSI) due to carbapenem-resistant *Klebsiella pneumoniae* (CRKP) in  patients with hematologic malignancies | Patients with haematological malignancies, hospitalised for more than 7 days between January 2017 and January 2018 | CRKP rectal colonisation, Severe neutropenia,IMV. | *Klebsiella pneumonia*, carbapenem-resistance | Medium | + (+ clinically relevant variables) | 7,3 | 42 | 3 | Variables with p<0.2 in the bivariable screening were included in the multivariable model, but also variables that were "clinically significant", which were not disclosed. The authors concluded that rectal colonisation with carbapenem-resistant *K. pneumoniae* increases the risk of such bloodstream infections. |
| 2017 | *Clostridium difficile* colonization in preoperative colorectal cancer patients | Zheng, Luo, Lv et al [41] | 205 | 33 | China | To reveal the rate  of *C. difficile* colonization and its correlation to clinical  characteristics in preoperative CRC patients | Consecutive preoperative CRC patients who were scheduled to accept radical cancer resection in a hospital in China, October, 2014 and August, 2015. | Age (<60 vs >60), Site (Colon vs Rectum), Morphology (Ulcerative vs Exophytic), Differentiation (Poor vs Well), T stage (Non-T4 vs T4), N stage (LNM neg. Vs LN pos.), Albumin (<35 vs >35), FBG (>7 vs <7), BMI, Triglyceride, Total cholesterol, HDL, Haemoglobin, OB | *Clostridioides difficile* | Medium | - | 2,4 | 14 | 14 | The authors concluded that colorectal cancer patients in China with large tumours or metastases were associated with a higher risk of *C. difficile* infections. |
| 2020 | Distribution, diagnosis, and analysis of related risk factors of multidrug-resistant organism in patients with malignant neoplasms | Zhou, Yang, Yang et al [26] | 278 | 128 | China | To investigate the distribution characteristics, early diagnosis, and  related risk factors of multidrug-resistant organism (MDRO) in patients with malignant tumors | Patients aged >18 years old and <75 years old with malignant tumour, positive bacterial culture during hospitalisation at Zhuji Hospital from January 2017 to December 2019 | Length of hospitalisation, Fever days, Invasive operation, Excessive bed rest, Hypoproteinemia, Procalcitonin, C Reactive Protein, Serum Amyloid | MDRO, non MDRO | Medium | + | 16,0 | 13 | 8 | Variables with p<0.001 in the bivariable screening were included in the multivariable model, except for excessive bedstay (p=0.002). The authors found that invasive operation, excessive bedstay, hypoproteinemia, PCT and SA were associated with MDRO infection in cancer patients. Measurements of such variables should help to prevent MDRO infection. |
