# Supplementary material for: Clinical outcomes of antimicrobial resistance in cancer patients: a systematic review of multivariable models
Source: BMC Infect Dis. 2023 Apr 18;23:247. doi: 10.1186/s12879-023-08182-3 (PMC10114324; doi:10.1186/s12879-023-08182-3)
Supplement: Supplementary file 4 — Additional file 4: Table S4. All articles included in the systematic review with a mortality outcome. [file 12879_2023_8182_MOESM4_ESM.docx]

# Supplementary material 4

**Table S4 - All included articles in the systematic review with a mortality outcome**

| **Year** | **Title** | **Authors** | **Number of patients** | **Number of events in the final (and largest) model** | **Country/setting** | **Study aim statement** | **Patient population** | **Factors included in the final model** | **Microbial etiology and resistance** | **Risk of bias grading (NIH tool)** | **Bivariable screening/stepwise regression** | **Events per variable in the final (and largest) model** | **Number of variables included in the tested/screened/initial model** | **Number of variables included in the final (and largest) model** | **Comments** |
| --- | --- | --- | --- | --- | --- | --- | --- | --- | --- | --- | --- | --- | --- | --- | --- |
| 2016 | Risk factors and clinical outcomes for vancomycin-resistant enterococcus bacteraemia in hospitalised cancer patients in Pakistan: A case-control study | Akhtar, Sultan, Nizamuddin et al. [148] | 111 | 57 | Pakistan | To identify the risk factors and outcomes for VRE  bacteraemia among cancer patients | Cancer patients whose blood culture grew either vancomycin-sensitive or resistant enterococcus (VSE or VRE) from January 2012 to December 2014 | Paediatric (Compared to adults), sex, type of malignancies, location in hospital at onset of bacteraemia, inpatient, others(OPD/ ER), APACHE-II score at onset of bacteraemia, length of hospital stay in days before onset of bacteraemia, shock at onset of bacteraemia), disseminated Intravascular Coagulation (DIC) score, received vancomycin within 4 weeks prior to bacteraemia | *Enterococcus spp*., vancomycin-resistance | Medium | No | 5.2 | 11 | 11 | The only risk factor that seems to be of importance for the mortality of patients with VRE vs VSE bacteraemia is shock at onset of bacteraemia. |
| 2019 | *Clostridium difficile* Infections in patients with AML or MDSundergoing allogeneic hematopoietic stem cell transplantationidentify high risk for adverse outcome | Amberge, Kramer, Schröttner et al [78] | 727 | Indeterminable | Germany | To study the effect of  CDI and CD colonization on the outcome in patients with  AML/MDS undergoing alloHCT | All patients with acute myeloid leukaemia or myelodysplastic syndrome who underwent allo hæmotological cell transplant at the institution between January 1st 2004 and March 31st 2015. | *Clostridium difficile* status (no infection/asymptomatic/symptomatic), donor type (related/unrelated), human Leukocyte Antigen compatibility (match/mismatch), disease risk (favourable/intermediate/high), age, meropenem exposition (yes/no) | *Clostridioides difficile* | Low | No | #VALUE! | 6 | 6 | The study indicated symptomatic *C. difficile* infection increases the risk of death |
| 2015 | Mortality burden related to infection with carbapenem-resistant Gram-negative bacteria among haematological cancer patients: a retrospective cohort study | Andria, Henig, Kotler et al [58] | 330 | 95 | Israel | To estimate the mortality burden of CRGNB among  haematological cancer patients receiving chemotherapy | Adult patients with Gram-negative aerobic bacteraemia hospitalised in the haemato-oncological/bone marrow transplantation departments actively treated for their malignancy between 2008-2014 | Dependent functional capacity at baseline, pulmonary disease, liver disease, hæmotologiv stem cell transplant comorbidities index, renal failure, admission-days before bacteraemia, type of the last chemotherapy treatment, functional capacity at bacteraemia onset, urinary catheter, nasogastric tube, type of bacteria, polymicrobial bacteraemia, appropriate empirical antibiotic treatment, CRGNB, systolic blood pressure , diastolic blood pressure, septic shock | Carbapenem-resistant Gram-negative bacteria | Medium | Yes | 5.6 | 28 | 9 | The authors found that a bacteraemia with a Carbapenem-resistant Gram-negative bacteria increases the risk of death, but the propensity score for resistance did not. |
| 2019 | Current etiology, clinical features and outcomes of bacteremia in olderpatients with solid tumors | Antonio, Gudiol, Royo-Cebrecos et al [27] | 217 | 77 | Spain | To assess the etiology,  clinical features, and outcomes among older patients with solid  tumors who developed bacteremia in the era of widespread antimicrobial  resistance. | All consecutive patients with solid tumours hospitalised with one or more episodes of bacteremia from January 2006 to November 2015. | Age years, lung cancer, prostate cancer, advance neoplasm, comorbidities, current corticosteroid therapy, bacteremia origin (healthcare/nosocomial), clinical manifestations (Low grade fever (<38 °C/Shock at presentation), source of bacteraemia (Urinary tract), inadequate empirical antibiotic therapy | Multidrug-resistant organisms | Low | Yes | 6.4 | 23 | 8 | There is both a model for early case-fatality (at 7 days) and 30-day mortality. The risk factors in this table is from the final 30-day mortality model, in which the author did not include resistant pathogens due to automatic variable selection. |
| 2019 | Colonization with multidrug resistant organisms determines the clinical course of patients with acute myeloid leukemia undergoing intensive induction chemotherapy | Ballo, Tarazzit, Stratmann et al [45] | 220 | 104 | Germany | The authors  hypothesized that colonization with a MDRO affects the clinical course of AML patients  undergoing intensive induction chemotherapy | All patients with acute myeloid leukaemia who underwent intensive induction chemotherapy between 2007 and 2015. | Age >60, adverse genetic group acute myeloid leukaemia, day 15 bone marrow blast clearance, stem cell transplantation as consolidation therapy, colonisation with carbapenem resistant enterococci | Multidrug-resistant organisms | Medium | Yes | 20.8 | 9 | 6 | Finds an increased risk of dying for AML patients colonised with carbapenem-resistant *enterobacteriaceae* |
| 2015 | Emergence of multidrug resistant isolates and mortality predictors in patients with solid tumors or hematological malignancies | Bastug, Kayaaslan, Kazancioglu et al [110] | 205 | 48 | Turkey | To identify the recent epidemiology of infections and mortality risk factors in infected neutropenic and non-neutropenic patients with hemato-oncological malignancies | Patients admitted to hemato-oncology wards of the 1,140-bed tertiary care hospital between 2008 and 2013. | Age > 65 years, prior hæmatologic stem cell transplant, prolonged neutropenia prior to infection, inadequate initial antibiotic treatment, gram-negative bacterial infection, infection with coagulase negative Staphylococci, infection with *Acinetobacter baumannii,* infection with *Klebsiella spp.*, infection with extended spectrum beta lactamase-producing bacteria, infection with Gram-negative multi drug resistant bacteria | Multidrug-resistant organisms | Medium | Yes | 4.8 | 10 | 10 | Finds an increased risk of dying among patients with predominantly haematological malignancies when infected with either *A. baumannii* or *Klebsiella spp*. |
| 2018 | The relationship between mortality and microbiological parameters in febrile neutropenic patients with hematological malignancies | Çalık, Arı Bilgir et al [57] | 106 | 58 | Turkey | To determine effective risk factors  on mortality in febrile neutropenic cases with  hematologic malignancy | Patients diagnosed with hematologic malignancies | Absolute neutrophil count, duration of neutropenia (days), catheterization focus of infection, central catheterization, isolation of Gram + bacteria in culture, isolation of Gram - bacteria in culture,extended spectrum beta lactamase *Enterobacteriaceae*, carbapenem resistance, imipenem empirical antibiotic regimen, bacterial growth during antibiotic treatment, administration of appropriate antibiotic treatment for minimum 72 hours, septic shock | Several bacterias, tested for sensitivity to all major classes of antibiotics. | Medium | Yes | 4.8 | 18 | 12 | Does not find an association between the resistant bacteria and mortality. |
| 2016 | Emerging resistant bacteria strains in bloodstream infections of acute leukaemia patients: results of a prospective study by the Rete Ematologica Lombarda (Rel) | Cattaneo, Zappasodi, Mancini et al [46] | 433 | 37 | Italy | To Describe the specific epidemiological scenario of  bacterial infections and antibiotic resistance in AL patients during  treatment, often affected by severe neutropaenia | All febrile/infectious episodes were recorded in patients with acute leukaemia from December 2012 to December 2014 | Complete remission, relapsed/refractory disease, central venous catheter-related bloodstream infection (BSI), multi-resistant Gram-negative BSI, pneumonia | Several bacteria and fungi, tested for various antibiotics | Medium | Yes | 7.4 | 20 | 4 | Authors found that multi-resistant Gram-negative bloodstream infections are associated with higher mortality |
| 2021 | A practical update on the epidemiology and risk factors for the emergence and mortality of bloodstream infections from real-world data of 3014 hematological malignancy patients receiving chemotherapy | Chen, Lin, Li et al [47] | 725 | 171 | China | To  characterize HM BSI and identify risk factors for BSI emergence and mortality | Patients with haematological malignancies, between 2013 and 2016 | Age>45, Hospital length of stay, Duration of neutropenia before blood culture, Hemograms of bloodstream infections (white blood cell, Haemoglobin, platelet), Disease status, Use of antibiotics (≥3 agents), Co-infections (respiratory), Numbers of co-infected locations, Pathogens for bloodstream infections (among them 11 bacterias, fungi, polymicrobial and other), gram negative organisms, gram positive organisms, polyorganisms, and fungi | Several bacteria and fungi, some intrinsically resistant | Medium | Yes | 6.3 | 27 | 27 | The authors found that the intrinsically resistant bacteria *S. maltophilia* and *A. baumannii* is associated with an increased hazard for mortality |
| 2018 | Association Between Positive Cultures During Admission and 1-Year Mortality in Patients With Cancer Receiving Perioperative Intensive Care | Chiang, Wu, Hsu et al [111] | 638 | 134 | Taiwan | To  address the long-term impact of having positive cultures during  admission on 1-year mortality among patients with cancer who  received perioperative intensive care by using both data in  cancer registry and electronic medical records | All adult patients who were registered in the hospital cancer registry and had at least one intensive care unit admission to an from 2011 through 2016 following their cancer diagnosis | Age, male gender, type II diabetes mellitus, cerebrovascular disease, congestive heart failure, metastatic cancer, emergent surgery, APACHE II, mechanical ventilation, positive culture of multi drug resistant organism, positive culture in blood/respiratory tract/urinary tract/skin and soft tissue | Several bacteria and fungi are studied, including MDROs (methicillin-resistant *Staphylococcus aureus*, vancomycin-resistant Enterococci, and carbapenem-resistant Gram-negative bacilli) | Medium | No | 12.2 | 11 | 11 | After adjusting for other factors, the authors did not find that MDROs were associated with a higher hazard of death. |
| 2015 | *Stenotrophomonas maltophilia* bloodstream infection in patients with hematologic malignancies: a retrospective study and in vitro activities of antimicrobial combinations | Cho, Lee, Choi et al [87] | 31 | 20 | South Korea | To investigate the clinical  characteristics and outcomes related to *S. maltophilia*  BSIs in patients with hematologic malignancies | All consecutive episodes of *S. maltophilia* bloodstream infections in adult patients with hematologic malignancies from June 2009 to May 2014. | Neutropenia at the onset of bloodstream infection, severe neutropenia at the onset of bloodstream infection (<100/mm3), duration of neutropenia >21 days, hospital stay >30 days, SAPS II score >40, Source of infection (Pneumonia), polymicrobial bloodtream infection, shock | *Stenotrophomonas maltophilia* | Medium | Yes | 6.7 | 9 | 3 | The authors found that shock, neutropenia and combined *S. maltophilia* pneumonia are associated with mortality in these patients. |
| 2020 | High mortality in an outbreak of multidrug resistant *Acinetobacter baumannii i*nfection introduced to an oncological hospital by a patient transferred from a general hospital | Cornejo-Juarez, Cevallos, Castro-Jaimes et al [141] | 106 | 53 | Mexico | To identify the epidemiology of the  outbreak and to describe the clinical evolution and risk factors for adverse outcomes of  patients during the outbreak of MDR *A. baumannii* | Patients with multi drug resistant *A. baumannii* in a tertiary care oncology hospital from January 2011 to December 2015 | Age <60, colonised/Infected, solid tumour/Hematologic malignancy, recent diagnosis (vs relapse), no recent chemotherapy (vs recent), appropriate treatment/Non-appropriate treatment, SOFA score <10/>10 | Multidrug-resistant *Acinetobacter baumannii* | Low | Yes | 7.6 | 7 | 7 | In this outbreak analysis, the authors found that haematological cancer patients had a higher risk of death than solid tumour patients |
| 2019 | Fungaemia in haematological malignancies: SEIFEM‐2015 survey | Criscuolo, Marchesi, Candoni et al [63] | 215 | 82 | Italy | We focused on epidemiology of fungal BSI in patients affected  by either myeloid or lymphoid HM admitted over the  past 5 years to 34 Italian Hematology centres participating to  the SEIFEM consortium | Hospitalised patients with haematological malignancies in which a documented fungaemia was diagnosed between January 2011 and December 2015 | Performance status >2, chronic kidney disease, steroid administration, gastrointestinal symptoms, respiratory symptoms, septic shock, multiorgan failure, concurrent bacterial sepsis, central venous catheter removal, neutrophil recovery, albicans vs non albicans | Several fungi, including intrinsically resistant | Low | Yes | 9.1 | 9 | 7 | In a univariate analysis, authors founnd that non-albicans candidaemias had a higher odds of death, but the factor is not retained in the final model |
| 2020 | Biofilm Production by Carbapenem-Resistant *Klebsiella pneumoniae* Significantly Increases the Risk of Death in Oncological Patients | Di Domenico, Cavallo, Sivori et al [149] | 53 | 19 | Italy | To analyze the impact of different CRKP virulence  determinants to assess their predictivity in supporting clinical  decision-making in high-risk oncological patients | Oncological patients colonised or infected with carbapenem resistant *K. pneumoniae* | Biofilm (strong vs weak), colistin resistance, fungal infection, phenotype, site (respiratory vs other) | Carbapenem-resistant *Klebsiella pneumoniae* | Medium | No | 3.8 | 5 | 5 | The authors did find that the production of biofilm in carbapenem-resistant *K. pneumoniae* is associated with a higher hazard of death |
| 2021 | The Impact of Bacterial Biofilms on End-Organ Disease and Mortality in Patients with Hematologic Malignancies Developing a Bloodstream Infection | Di Domenico, Marchesi, Cavallo et al [48] | 96 | 8 | Italy | To explore putative risk factors for the development of BSIs and mortality  caused by MDR and biofilm-growing bacteria in a cohort of patients with HMs | All consecutive adult patients (aged >18 years) affected by haematological malignancies and experiencing a bloodstream infection as detected from April 2016 through April 2019 | Antimicrobial prophylaxis, multi drug resistant (MDR) vs non-MDR bloodstream infection (BSI), initial antimicrobial failure, P. aeruginosa vs other BSI, strong vs weak biofilm-producer, septic shock, end organ disease | Several bacteria, including intrinsically resistant tested for resistance towards several antibiotics | Medium | Yes | 1.1 | 16 | 6 | The authors found that a multidrug-resistant bloodstream infection is associated with a higher hazard of death in these patients |
| 2021 | Clinical characteristics and outcome of 125 polymicrobial bloodstream infections in hematological patients: an 11‑year epidemiologic survey | Facchin, Candoni, Lazzarotto et al [49] | 125 | 19 | Italy | To investigate the clinical microbiological  characteristics and outcome of pBSI that  were consecutively observed in hematological patients  admitted to our Hematology Department to receive intensive  chemotherapy or stem cell transplantation (SCT) | Patients affected by haematological neoplasms with a documented polymicrobial bloodstream infection | Age, infection related mortality, septic shock rate, multi drug resistant-polymicrobial bloodstream infections mortality | Several bacteria tested for resistance towards several antibiotics | Medium | Yes | 4.8 | 13 | 4 | The authors found that in these patients, having a multidrug-resistant bacteria as part of a polymicrobial bloodstream infection is associated with a higher odds of death |
| 2015 | Early initiation of appropriate treatment is associated with increased survival in cancer patients with *Candida glabrata* fungaemia: a potential benefit from infectious disease consultation | Farmakiotis, Kyvernitakis, Tarr et al [157] | 146 | 58 | USA | To  investigate the correlation of clinical outcomes with early  initiation of appropriate treatment and ID consultation, in a  contemporary cohort of cancer patients with *C. glabrata*  fungaemia | Patients with at least one blood culture(s) positive for C. Glabrata at a cancer centre between March 2005 and September 2013 | Intensive care unit stay, monocytopenia, APACHE II score, mechanical ventilation, septic shock, acute kidney infection or acute renal failure, fever, corticosteroids, catheter-related candidemia, mixed bloodstream infection, caspofungin resistance, echinocandin pre-exposure, central venous catheter removal within 48 h after blood culture collection, appropriate treatment within 48 h after blood culture collection | *Candida glabrata* | Medium | Yes | 4.1 | 18 | 7 | The authors concluded that patients with a blood culture positive for *C. glabrata* should consult an ID specialist within 48 hours |
| 2021 | Fluoroquinolone treatment as a protective factor for 10‑day mortality in *Streptococcus pneumoniae* bacteremia in cancer patients | Fontana, Ibrahim, Bonazzi et al [165] | 161 | 34 | Brazil | To evaluate prognostic  factors in cancer patients in episodes of pneumococcal bacteremia and to describe the epidemiological, clinical  and microbiological data, the phenotypic characteristics of the collected specimens (serotypes and antimicrobial  resistance) and to correlate the S. pneumoniae serotypes with available vaccine coverage | Patients attended between January 2009 and July 2015 at a cancer institute with haematological malignancies and / or solid tumours, over 18 years of age, and who had at least one episode of *S. pneumoniae* bacteremia during the referred period | Ethnicity, diabetes Mellitus, neutropenia past month, current neutropenia, severe neutropenia past month, current severe neutropenia, febrile neutropenia, SOFA score, polymicrobial bacteremia, vaccine serotype VPV23, 3rd and 4th generation cephalosporins, fluoroquinolones. | *Streptococcus pneumoniae*, tested for resistance against several antibiotics | Low | Yes | 2.8 | 53 | 21 | While the authors test for association between antimicrobial susceptibility and mortality, they conclude that there is no association and do not include it in the final model |
| 2016 | Bloodstream infection caused by extensively drug-resistant *Acinetobacter baumannii* in cancer patients: high mortality associated with delayed treatment rather than with the degree of neutropenia | Freire, de Oliveira Garcia, Garcia et al. [142] | 92 | 77 | Brazil | To describe severe  infections with XDR Acinetobacter baumannii–calcoaceticus  complex (XDR-ABC), as well as to investigate risk factors for  mortality, in cancer patients | All patients diagnosed with extensively drug resistant *A. baumannii* bacteraemia, acquired during hospitalisation in the intensive care unit at a cancer institute | Serum albumin, septic shock at diagnosis of XDR-ABC bacteraemia (extended drug resistant), SOFA score at diagnosis, received appropriate therapy for XDR-ABC bacteraemia, combination therapy, days from 1rst positive culture to initial treatment, removal of invasive devices within the first 48 h | *Acinetobacter baumannii*, extensively drug resistance | Low | Yes | 11.0 | 20 | 6 | The authors found that 68% of all healthcare-associated bacteraemias are caused by extensively drug resistant *A. baumannii*, which is associated with a 30-day mortality of more than 80 %. The authors concluded that the only risk factor of importance was appropriate antibiotic treatment. |
| 2018 | Role of Lock Therapy for Long-Term Catheter-Related Infections by Multidrug-Resistant Bacteria | Freire, Pierotti, Zerati et al [112] | 212 | 212 | Brazil | To analyze LTCVC-associated infections in terms of risk factors for unfavorable outcomes  and the impact of ALT, including infection by MDR bacteria | All LTCVC-associated infections diagnosed at the a cancer institute between January 2009 and December 2016 | Haematological malignancies, Palliative care, SOFA score, MDR bacteria, Adherence to protocol, Antibiotic lock therapy | Several bacteria and fungi, tested for resistance towards several antibiotics | Low | + | 15,1 | 28 | 14 | The authors found that there is an increased hazard of death when the patient is infected by multidrug-resistant bacteria, but that antibiotic lock therapy is associated with a decrease in death. |
| 2015 | Infection with *Klebsiella pneumoniae* carbapenemase (KPC)-producing *Klebsiella pneumoniae* in cancer patients | Freire, Pierrotti, Filho et al [150] | 83 | 49 | Brazil | To describe HAIs due to KPC-Kp  in patients with solid tumors and hematologic malignancies.  In addition, we attempted to identify the risk factors associated  with mortality in such cases | All patients diagnosed with an healthcare associated infection due to Klebsiella producing carbapenemase-*Klebsiella pneumoniae* acquired during hospitalisation at a cancer institute between January 2009 and July 2013 | Serum albumin, concomitant infection, bloodstream infection, urinary tract infection, SOFA score at healthcare associated infection (HAI) diagnosis, removal of invasive device or control deep infection, received effective therapy for Klebsiella producing carbapenemase-Kp infection, intensive care unit admission for healthcare associated infection (HAI), mechanical ventilation, acute kidney injury within 30 days after HAI diagnosis | *Klebsiella pneumoniae*, carbapenemase-producing | Low | Yes | 4.5 | 27 | 11 | The authors concluded that there is high mortality in patients with KPC-Kp infection, and that mortality is associated with the severity of the infection rather than the underlying disease |
| 2018 | Risk factors for mortality in patients with acute leukemia and bloodstream infections in the era of multiresistance | Garcia-Vidal, Cardozo-Espinola, Puerta-Alcalde et al [50] | 589 | 87 | Spain | To describe the current epidemiology and its changes during the different cycles  of chemotherapy of BSI in a large current cohort of patients with AL. The authors also assessed the risk  factors for BSI mortality and the risk factors for BSI caused by MDR-Pseudomonas aeruginosa  (MDR-PA) | All consecutive episodes of bloodstream infection occurring in patients with acute leukaemia from July 2004 to February 2016. | Older age (>65 years old), diabetes mellitus, chronic lung disease, chronic liver disease, prolonged neutropenia, mucositis, fatal prognosis according to McCabe index, community site of acquisition, catheter as a source of bacteremia, pulmonary source of infection, intra abdominal source of infection, cONs, *E.coli, P.aeruginosa*, shock at onset, inappropriate antibiotic therapy for multi drug resistant strains, inappropriate antibiotic therapy for multi drug resistant *P. aeruginosa* | Several bacteria and fungi, tested for resistance towards several antibiotics. A special focus on multidrug-resistant *Pseudomonas aeruginosa*. | Low | Yes | 5.1 | 34 | 17 | The authors found an association between multidrug-resistant *P. aeruginosa* and mortality, after adjusting for some other confounders and risk factors. |
| 2017 | Trends in the Incidence and Outcomes of Hospitalized Cancer Patients With Clostridium difficile Infection: A Nationwide Analysis | Gupta, Tariq, Frank et al [137] | 20,100,000 | Indeterminable | USA | To evaluate the incidence of CDI  in patients with both solid organ and hematologic  cancers, incidence trends over time, and outcomes  in these patients compared with those with cancer  without CDI | Cancer hospitalizations in a national cancer database from 2001 to 2010. | *C. difficile* infection, age, sex, admission status, and comorbidities. | *Clostridioides difficile* | Medium | No | Indeterminable | Indeterminable | Indeterminable | The authors found that CDI is associated with a higher mortality among cancer patients. It was not clear how many factors variables were included in this model but we assumed 12 confounders and 2 factors (CDI and dismissal to care facility) |
| 2021 | Colonization with multidrug‑resistant organisms impairs survival in patients with hepatocellular carcinoma | Himmelsbach, Knabe, Ferstl et al [28] | 954 | 476 | Germany | To determine the  incidence of MDRO-colonization in HCC patients and to  evaluate its impact on the clinical course | Patients with confirmed hepatocellular carcinoma presenting between January 2008 and December 2017 | Age, sex, underlying cirhhosis, Child–Pugh class C vs. A or B, BCLC stage C or D vs. A or B, ALBI grade 3 vs. grade 1 or 2, Alpha-fetoprotein > 400 ng/ml, sorafenib treatment, resection as hepatocellular carcinoma treatment, multi drug resistant organism-colonization | Several bacteria, tested for resistance towards several antibiotics | Medium | Yes | 95.2 | 10 | 5 | In this study, the authors concluded that there is an association between colonisation with a multidrug-resistant organism and mortality in patients with hepatocellular carcinoma. |
| 2018 | Bloodstream infections in cancer patients. Risk factors associated with mortality | Islas-Muñoz, Volkow-Fernández, Ibanes-Gutiérrez et al [113] | 496 | 109 | Mexico | To evaluate the clinical epidemiological characteristics and risk factors associated with  mortality in cancer patients with BSI, with special emphasis on  MDR bacteria | From August 2016 to July 2017, all positive blood cultures detected at the Microbiology Laboratory at a cancer centre were recorded, and all episodes confirmed as bloodstream infection were included in the study. | Age <60 years vs. Age >60 years, Solid tumour vs Haematological, Recent diagnosis or remission vs Progression/relapse, Gram-positive vs Gram-negative, Monomicrobial vs Polymicrobial, Appropriate treatment vs Inappropriate treatment, Non-multidrug resistant (MDR) vs MDR and/or extended spectrum beta lactamase, Neutrophils >500 vs Neutrophils 500 | Several bacteria, tested for resistance towards several antibiotics | Low | No | 13.6 | 8 | 8 | Authors included every variable of the univariate in the multivariate despite it being written that inclusion depended on p<0.5. The authors concluded that there was an association in the 30-day mortality among the cancer patients and bloodstream infection with multidrug-resistant Gram-negative bacteria, particularly if it was inappropriately treated. |
| 2020 | Nosocomial Infections in Gastrointestinal Cancer Patients: Bacterial Profile, Antibiotic Resistance Pattern, and Prognostic Factors | Jiang, Liu, Said et al [29] | 428 | Indeterminable | China | To explore the bacterial profile, antibiotic resistance pattern, and prognostic  factors of nosocomial infections in hospitalized GI cancer patients | Hospitalised gastrointestinal cancer patients with nosocomial infections at a cancer treatment centre from August 2013 to June 2019 | Gram negative bacteria, *E.coli, K. pneumoniae, Enterobacter* spp, Fungi, *C. albicans. Candida* spp, enterococcus | Several bacteria and fungi, tested for resistance towards several antimicrobials | Medium | Yes | Indeterminable | 24 | 8 | There are three full multivariable models in this article that all estimate the OR for mortality associated with different factors; a demographic/clinical model, an infection-related model, and a causative pathogens model. Only the causative pathogens model is assessed as the other models don't explicitly include antimicrobial resistance. One is about resistant pathogens. The authors concluded that Gram-negative bacteria were most common. |
| 2020 | Nosocomial infections due to multidrug-resistant bacteria in cancer patients: a six-year retrospective study of an oncology Center in Western China | Jiang, Shi, Liu et al [118] | 257 | 28 | China | To  evaluate the characteristics, antibiotic resistance patterns, and prognosis of nosocomial infections due to multidrugresistant (MDR) bacteria in cancer patients | Cancer patients with nosocomial infections due to multi drug resistant bacteria who received medical care during hospitalisation from August 2013 to May 2019 | Clinical model: Demographic data (age), Smoking history (Never smoker/Former smoker/Current smoker), ECOG performance status (0,1/2,3,4), Existence of distant metastasis, Comorbidities (Liver disease), Charlson comorbidity index CCI (1–2), CCI≥3, Surgery, Chemotherapy, Intrapleural/abdominal infusion (within 30 days), Presence of indwelling urinary catheters, presence of Drains postoperative; Infection-related model: Sample type (Urine/Blood culture), Primary sites of infection (Respiratory tract/Urinary tract/BSI), Length of hospitalisation (≥21.0), Septic shock, Laboratory examination results (Haemoglobin g/L <110.0, Platelet count ×109/L <100.0, Lymphocytes count ×109/L <1.0, PCT ng/mL ≥1.0, Albumin g/L <30.0) | Methicillin resistant *S. aureus* (MRSA), Extended spectrum beta lactamase producing *Enterobacteriaceae* (ESBL-PE), multi drug resistant (MDR) *Pseudomonas aeruginosa, Acinetobacter baumannii, Stenotrophomonas maltophilia*, Carbapenem-resistant *Enterobacteriaceae* | Low | Yes | 2.5 | 40 | 11 | There are two full multivariable models in this article that both are assessed, as both have antimicrobial resistance as factors. Numbers are extracted from the nested model with the less variables tested, hence the highest number of events by variable in theory. Authors explained they included variables form univariate in MVA when p<0.1 but they included other factors as well. The most common bacteria were ESBL-PE, and the authors conclude that former smokers, intrapleural/abdominal infusion history within 30 days, presence of indwelling urinary catheters, and anaemia were independent risk factors for in-hospital mortality of nosocomial infections caused by MDR bacteria. |
| 2020 | Is current initial empirical antibiotherapy appropriate to treat bloodstream infections in short-duration chemo-induced febrile neutropenia? | Joncour, Puyade, Michaud et al [114] | 27 | 13 | France | To assess the appropriateness of antibiotherapy and the outcome of bloodstream infections  (BSI) in patients with expected neutropenia of short duration | Febrile neutropenic patients at a hospital undergoing chemotherapy for solid tumours or malignant haemopathy with expected duration of neutropenia ≤7 days from 1 January 2015 to 31 December 2016. | Signs of severity, documented clinical source, qSOFA score ≥2 | Several bacteria, tested for resistance towards several antibiotics | Medium | Yes | 4.3 | 18 | 3 | The authors found that the only risk factor associated with mortality was the qSOFA score, and found no association between mortality and a history of infection/colonisation with MDR *P. aeruginosa*. Numbers are extracted from the model table in Supplementary material |
| 2015 | Uncommon Candida Species Fungemia among Cancer Patients, Houston, Texas, USA | Jung, Farmakiotis, Jiang et al [158] | 66 | 40 | USA | The authors aimed to investigate the epidemiology, antifungal use, susceptibility  patterns, and factors associated with all-cause death  among cancer patients in whom uncommon *Candida spp.* were diagnosed at a cancer treatment center | Patients ≥18 years of age hospitalised in the cancer centre during January 1998–September 2013. | Underlying leukaemia, steroid exposure, intensive care unit admission, intubation, persistent neutropenia, APACHE IIscore≥19, hypoalbuminemia, breakthrough fungemia | *Candida* spp. | Medium | Yes | 5.0 | 9 | 8 | The authors showed that ICU, neutropenia and high APACHE score, were the strongest risk factors for mortality. |
| 2019 | Impact of empiric treatment for vancomycin-resistant Enterococcus (VRE) in colonized patients early after allogeneic hematopoietic stem cell transplantation | Kamboj, Cohen, Huang et al [95] | 95 | 5 | USA | To compare all-cause mortality at 30 days in patients who received  empiric versus (vs) directed VRE therapy | Adults 18 years and older who underwent allo-hæmotologic stem cell transplantation and developed vancomycin resistant enterococcus bacteremia between January 1, 2005 and December 31, 2014, at the cancer centre | T cell depletion, prior vancomycin resistant enterococcus bacteremia (VREB), hypotension (<90/50), persistent VREB >48 hours | *Enterococcus* spp., vancomycin-resistance | Medium | Yes | 1.3 | 13 | 4 | The authors found there is no association with higher mortality at day 30 and any of tested variables in VREB cancer patients |
| 2019 | Contribution of specific pathogens to bloodstream infection mortality in neutropenic patients with hematologic malignancies: Results from a multicentric surveillance cohort study | Kern, Roth, Bertz et al [54] | 1424 | 36 | Germany, Austria, Switzerland | The authors analyzed  early and late mortality rates after BSI as well as its predictors | Patients with bloodstream infection during neutropenia subsequent to high‐dose chemotherapy for acute leukaemia (HDC) or autologous or allogeneic hematopoietic stem cell transplantation (auto‐HSCT or allo‐HSCT) from the beginning of 2002 through April 2015 | Mortality 30 days model : Auto hematologic stem cell transplant treatment, CoNS (coagulase negative staphylococci). Mortality 7 days model: coNS | Several bacteria, tested for resistance towards several antibiotics | Low | Yes | 18.0 | 5 | 2 | There are 2 mortality models in Annex, and one model about ICU admission which we did not assess. Numbers are extracted from the mortality 30 days model. In this relatively large cohort, the authors found that there is an association between the infecting pathogen and 7 day mortality after BSI - particularly *Klebsiella, Enterobacter, Serratia*, and *Pseudomonas*. |
| 2019 | Pathogenic significance of hemorrhagic pneumonia in hematologic malignancy patients with *Stenotrophomonas maltophilia* bacteremia: clinical and microbiological analysis | Kim, Cha, Kang et al [88] | 118 | 72 | South Korea | To evaluate the risk factors for mortality in hematologic malignancy patients with SMB and to analyze clinical and  microbiological characteristics of HP associated with SMB and CRBSI | Patients ≥18 years of age with hematologic malignancy who were diagnosed with *S. maltophilia* bacteremia | Age, hospital stay, polymicrobial infection, previous isolation of *S. maltophilia*, Focus of infection catheter related infection (CRI), focus of infection hemorrhagic, CRI non hemorrhagic, leukaemia, lymphoma, refractory or recurrent disease, Charlson comorbidity index I>3, neutropenia, intensive care unit stay, mechanical ventilation, renal Replacement Therapy, initial SOFA score, platelet count, previous antibiotics <3, early empirical TMP/SMX antibiotic use within 72 h | *Stenotrophomonas maltophilia* | Low | Yes | 3.8 | 27 | 19 | The authors also included clinically relevant variables in the MVA. The authors concluded that the mortality of haematological malignancy patients with *S. maltophilia* bacteraemia is high, and that hemorrhagic pneumonia is associated with an increased mortality |
| 2020 | Vancomycin-resistant enterococci infection and predisposing factors for infection and mortality in patients with acute leukaemia and febrile neutropenia | Kirkizlar, Akalin, Kirkizlar et al [96] | 179 | 17 | Turkey | To analyse the incidence of VRE  infections and mortality rates in VRE colonized adult acute leukemia  patients with febrile neutropenia (FN) | Adult acute leukaemia patients with vancomycin resistant enterococcus colonised who had febrile neutropenia | Age, sex, primary disease acute myeloid leukemia, remission, previous hospitalisation, previous colonization, exposure to empirical antibiotics, mucositis, central venous catheter, invasive procedures, < 0,5x109/L neutrophil count while vancomycine resistant enterococcus (VRE) +, coinfection, acute kidney injury, >15 days VRE | *Enterococcus* spp. , vancomycin-resistance | Low | No | 1.2 | 14 | 14 | Apart from age and gender, the authors found an association between in-hospital mortality in VRE-colonised patients and low neutrophil count and coinfection |
| 2017 | Initial Treatment of Cancer Patients with Fluconazole-Susceptible Dose-Dependent Candida glabrata Fungemia: Better Outcome with an Echinocandin or Polyene Compared to an Azole? | Le, Farmakiotis, Tarrand et al [159] | 68 | 26 | USA | The authors performed a retrospective review of clinical and laboratory data for patients with  *C. glabrata* fungemia caused by strains with dose-dependent in vitro susceptibility to  fluconazole | Patients with *C. glabrata* fungemia caused by strains with dose-dependent in vitro susceptibility to fluconazole at a cancer centre between March 2005 and September 2013. | APACHE II score, absolute monocyte count<100, intensive care unit, azole monotherapy | *Candida glabrata* | Medium | Yes | 2.9 | 17 | 9 | The authors found that azole monotherapy was associated with higher mortality than compared to the addition of an echinocandin or polyene in these patients |
| 2021 | Epidemiology, Risk Factors, and Clinical Outcomes of Bloodstream Infection due to Extended-Spectrum Beta-Lactamase-Producing *Escherichia coli* and *Klebsiella pneumoniae* in Hematologic Malignancy: A Retrospective Study from Central South China | Liang, Xu, Cheng et al [66] | 449 | Indeterminable | China | To determine the epidemiology, risk factors, and prognosis of extended-spectrum beta-lactamase  (ESBL)-producing *Escherichia coli* and *Klebsiella pneumoniae* bloodstream infections (BSIs) among hematology malignancy (HM) patients in China | Patients with haematological malignancies who were diagnosed with extended spectrum beta lactamase (ESBL)-producing and non-ESBL-producing *E. coli* and *K. pneumoniae* bacteremia between January 2010 and July 2018 | Myelodysplastic syndrome, MASCC score <21, Charlson Comorbidity index>3 | *Escherichia coli* and *Klebsiella pneumoniae*, ESBL | Medium | Yes | Indeterminable | Indeterminable | Indeterminable | The authors did not find a difference in overall mortality between the ESBL and non-ESBL groups, but they do find that the Charlson Comorbidity Index and the MASCC score were associated with an increased mortality in the ESBL-group. Number of deaths extracted from supplementary material. |
| 2015 | Factors influencing mortality in neutropenic patients with haematologic malignancies or solid tumours with bloodstream infection | Marin, Gudiol, Ardanuy et al [115] | 510 | 61 | Spain | To identify factors influencing mortality in neutropenic patients with haematologic malignancies or solid  tumours with bloodstream infection (BSI) | Hospitalised adult neutropenic cancer patients with at least one episode of bloodstream infection | Haematological malignancies: age, sex, advanced neoplasm, MASCC score <21, corticosteroid therapy, coagulase-negative staphylococci, multi drug resistant gram negative bacteria, Empirical antibiotic combination therapy, intensive care unit admission. Solid tumours: age, sex, advanced neoplasm, corticosteroid therapy, septic shock. | Several bacteria, tested for resistance towards several antibiotics | Low | Yes | 6.8 | 40 | 9 | The authors used stepwise regression but also included some clinically relevant variables. The authors stratified the population and made one model for haematological malignancies and one model for solid tumours. The numbers were extracted from the hematologic model, with a higher number of events. They only found an association between antimicrobial resistance and mortality among haematological cancer patients |
| 2019 | Bloodstream infection in patients with head and neck cancer: a major challenge in the cetuximab era | Marin, Gudiol, Castet et al [30] | 51 | 7 | Spain | To assess the impact of bloodstream infection (BSI) in patients with head and neck cancer (HNC) | Consecutive episodes of bloodstream infection occurring in patients with head and neck cancer and bloodstream infection from January 2006 to April 2017 | Early case fatality: age, sex, Charlson scoring system, median albumin level, corticosteroid therapy. Overall case fatality: age, sex, Charlson scoring system, comorbidities, median Albumin level, corticosteroid therapy. | Several bacteria and fungi, tested for resistance towards several antimicrobials | Low | Yes | 1.2 | 39 | 6 | The authors used stepwise regression but also included some clinically relevant variables. Both models investigating fatality include antimicrobial resistance as a factor, but it is removed after the bivariable screening. Numbers were extracted from the early fatality model. There is also a matched case-control study investigating the risk factors of all BSIs but it does not include any resistance. |
| 2020 | Inappropriate Empirical Antibiotic Treatment in High-risk Neutropenic Patients With Bacteremia in the Era of Multidrug Resistance | Martinez-Nadal, Puerta-Alcalde, Gudiol et al [116] | 251 | 87 | Spain | To describe the current rates of inappropriate empirical antibiotic treatment (IEAT) in oncohematological  patients with febrile neutropenia (FN) and its impact on mortality | All consecutive episodes of bacteremia in patients with high-risk febrile neutropenia from January 2006 to January 2017 | Inappropriate empirical antibiotic therapy, septic shock at onset, pneumonia | Several bacteria tested for resistance towards several antibiotics. The model is on *Pseudomonas aeruginosa*. | Low | Yes | 29.0 | Indeterminable | 3 | The authors found that inappropriate empirical antibiotic therapy, septic shock and pneumonia is associated with mortality among patients with *P. aeruginosa* bloodstream infection. The area under the curve of this model is printed. |
| 2018 | Postoperative empyema following lung cancer surgery | Matsutani, Yoshiya, Chida et al [31] | 43 | 5 | Japan | The authors investigated the risk factors for developing postoperative empyema following lung cancer surgery and its prognosis | Patients who underwent thoracic surgery to treat primary lung cancer and had empyema between January, 2008 and December, 2012, from 9 institutions | Exacerbation of interstitial pneumonia, bronchial stump fistula, administration of steroid, interstitial pneumonia, histology with or without squamous cells, microbiology with or without non-fermenting Gram(-) bacilli | Several bacteria, tested for resistance towards several antibiotics | Medium | Yes | 0.8 | 35 | 6 | The authors used stepwise regression but it is unclear whether they used p-value<0.05 to include factors of the univariate in the final model. they exclude one significant factor with no observation in one group. No association with poor prognosis in empyema patients after lung cancer surgery were found through the multivariable model. |
| 2017 | Fecal Carriage of Extended-Spectrum b-Lactamase-Producing Enterobacteriaceae Strains Is Associated with Worse Outcome in Patients Hospitalized in the Pediatric Oncology Unit of Beni-Messous Hospital in Algiers, Algeria | Medboua-Benbalagh, Touati, Kermas et al [125] | 171 | 28 | Algeria | To investigate extended-spectrum b-lactamase-producing *Enterobacteriaceae* (ESBL-E) fecal carriage in children with different cancers admitted in the pediatric oncology  unit of Beni-Messous Hospital (Algiers, Algeria) | Paediatric oncology patients from February 2012 to May 2013 | Age, gender, previous hospital admission, haematological malignancies, antibiotic treatment for the last 3 months before admission, haematological malignancies, Nephroblastoma, neuroblastoma, sarcomas, extended spectrum beta lactamase enterobacteria (ESBL-E) | *Enterobacteriaceae*, ESBL | Medium | Yes | Indeterminable | Indeterminable | Indeterminable | No regression table is printed, but the variables that the authors report to be associated with the two outcomes are reported here. During screening, the authors find that more than half of the patients are carriers of ESBL-producing *Enterobacteriaceae*. |
| 2021 | Secondary Infections After Diagnosis of SevereRadiation Pneumonitis (SRP) Among Patients With Non-Small Cell Lung Cancer: Pathogen Distributions, Choice of Empirical Antibiotics, and the Value of Empirical Antifungal Treatment | Mei, Yang, Yu et al [32] | 777 | 174 | China | To assess pathogen distributions and antimicrobial sensitivity characteristics in patients with nonsmall cell lung cancer (NSCLC) with severe radiation pneumonitis (SRP) and secondary infections | Patients with severe radiation pneumonitis treated at a hospital between January 2009 and December 2020 with non-small cell lung cancer, who were infected after at least 48 hours of hospitalisation and received at least 1 antibiotic before susceptibility testing. | Performance status score, chronic obstructive pulmonary disease, diabetes, hypertension, radiotherapy dose in Gy, multiple bacterial infections, bacteria/fungal coinfections, appropriate antibiotic, empirical antifungal | Several bacteria and fungi, tested for resistance towards several antimicrobials | Medium | Yes | 19.3 | 21 | 9 | The authors used stepwise regression but it is unclear if the authors used a p-value<0.05 to include the factors tested in the univariate in the final model. The authors did not find that there is any association between intrinsically resistant bacteria and infection-related mortality |
| 2021 | Predictive factors associated with induction‑related death in acute myeloid leukemia in a resource‑constrained setting | Mendes, da Silva, da Costa Bandeira de Melo et al [55] | 206 | 53 | Brazil | To comprehensively explore which baseline clinical and laboratory  features are associated with induction mortality in a LMIC  center and to verify whether different prophylactic strategies  employed throughout the years have impacted on this outcome | Patients aged >=16 years with newly diagnosed acute myeloid leukaemia who started any regimen of intensive treatment | ECOG score binary, sex, age, intensive care unit entrance, Charlson comorbidities index (CCI), colonisation by *Klebsiella* producing carbapenemase, colonisation by *Acinetobacter*, colonisation by *Pseudomonas*, DM, tumor lysis, pre-chemo infection, Albumin to Globulin Ratio, monocytic acute myeloid leukaemia, monocytes, C reactive protein, prothrombine activity, D-Dimer, creatinin, albumin, alkaline phosphatase, total bilirubine, aciclovir, antibiotiv prophylaxis, anthracycline | Several bacteria and fungi, tested for resistance towards several antimicrobials | Low | Yes | 2.1 | 56 | 25 | The authors used stepwise regression but excluded ECOG in the stepwise approach due to the high missing rate found during the chart reviewing. The final multivariable model showed that age>60 years, Gram-negative colonisation, monocytic AML, CRP>15 mg/dL, and an adverse risk in the genetic stratification were independently associated with early mortality in AML patients |
| 2021 | Reduced mortality from KPC-*K.pneumoniae* bloodstream infection in high-risk patients with hematological malignancies colonized by KPC-*K.pneumoniae* | Micozzi, Gentile, Santilli et al [100] | 34 | 10 | Italy | The authors evaluated the impact on KPC-KpBSI mortality of the preemptive use of antibiotics active against KPC-*K.pneumoniae*, as opposed to inactive or standard empiric  antibiotics, for the empiric treatment of febrile neutropenia episodes in patients with hematological malignancy  identifed as KPC-*K.pneumoniae* intestinal carriers | Patients with haematological malignancies identified as Klebsiella producing carbapenemase -*K. pneumoniae* carriers attending a haematology department between March 2012-December 2013 (Period 1) and January 2017-October 2018 (Period 2). | Model 1: *Klebsiella* producing carbapenemase-*K. pneumoniae* (KPC-KP) bloodstream infection (BSI) developing during inactive antibiotic treatment, acute myeloid leukaemia, shock, intensive chemotherapy. Model 2: Initial active treatment, KPC-KP BSI developing during inactive antibiotic treatment, acute myeloid leukaemia, shock, intensive chemotherapy. | *Klebsiella pneumoniae*, carbapenemase-producing | Medium | Yes | 2.5 | 22 | 4 | The numbers were extracted from the Model 1. It is unclear if authors used stepwise with p-value<0.05 to include factors of the univariate in the final model, and it is also unclear which factors are included or kept in the model. The authors found that 3 of 10 patients that died were put on initially active treatment |
| 2015 | Outcomes of critically ill cancer patients with *Acinetobacter baumannii* infection | Ñamendys-Silva, Correa-García, García-Guillén et al [143] | 23 | 6 | Mexico | To describe the ICU outcomes of  critically ill cancer patients with AB infection | Cancer patients who acquired *Acinetobacter baumannii* infections during their stay at the intensive care unit of a cancer institute | Length of hospital stay, duration of vasopressors, blood lactate level (mmol/L), number of organ dysfunction, creatinine, | *Acinetobacter baumannii* | Medium | Yes | 1.2 | 14 | 5 | The authors found that the blood lactate level was associated with mortality in these patients |
| 2020 | Characteristics and Clinical Outcomes of Extended-Spectrum beta-lactamase-producing *Klebsiella pneumoniae* Bacteremia in Cancer Patients | Nham, Huh, Cho et al [151] | 267 | 46 | South Korea | To evaluate possible risk factors and outcomes of extended-spectrum beta-lactamase producing *Klebsiella pneumoniae* (ESBL-KP) bacteremia in cancer patients | Patients above the age of eighteen with cancer who had an episode of *K. pneumoniae* bacteremia between 2010 and 2012 | 30-day mortality model: Neutropenia, foley catheter, nasogastric tube, mechanical ventilation, hemodialysis, catheter related infection, 14-day mortality model: solid tumour, extended spectrum beta lactamase, chronic liver disease, mechanical ventilation, hemodialysis, catheter related infection, PITT bacteremia score, carbapenem empirical therapy, vancomycin empirical therapy | *Klebsiella pneumoniae*, ESBL | Medium | Yes | 7.7 | 51 | 6 | There are two models, one on 14 days mortality and one about 30 days mortality. Numbers were extracted from the 30 day mortality model. The authors found that in these patients and with this statistical approach, there was an association between ESBL-production and 14-day mortality, but not with 30-day mortality |
| 2015 | Impact of vancomycin-resistant enterococcal bacteremia on outcome during acute myeloid leukemia induction therapy | Ornstein, Mukherjee, Keng et al [97] | 350 | 84 | USA | To  determine the rates of VRE bacteremia in patients with AML  receiving IC, and whether VRE bacteremia is an independent  predictor of inferior overall outcome in these patients | Patients with de novo and secondary acute myeloid leukaemia who received cytarabine-based induction chemotherapy at Cleveland Clinic between 1 January 2000 and 1 April 2008 | Age (>60 vs. <60), cytology Poor vs. Favourable, cytology Poor vs. Intermediate, cytology Poor vs. Unknown, VRE bacteremia, other bloodstream infections, aetiology (secondary vs. de novo), white blood cell at diagnosis, year of diagnosis | *Enterococcus* spp., vancomycin-resistance | Medium | Yes | 9.3 | 14 | 9 | The authors included factors which were significant in the univariate analysis plus blood cell count, year of diagnosis and non VRE BSI. conclude that the survival is poorer in AML patients who had a VRE bacteraemia. |
| 2020 | *Candida* spp bloodstream infections in a Latin American Pediatric Oncology Reference Center: Epidemiology and associated factors | Paixao de Sousa da Silva, de Moraes-Pinto, Teofilo Pignati et al [160] | 90 | 90 | Brazil | To characterize *Candida spp* bloodstream infections (BSI) in a reference  centre for paediatric oncology and to describe the most prevalent risk factors associated with candida infections | Paediatric patients admitted to a paediatric oncology institute who presented with *Candida* spp. BSI from January 2004 to December 2016. | Age, Hospitalisation, ICU, Corticosteroid, Prior antifungal use, Skin lesion, Dissemination, *Candida* spp Species (*C parapsilosis, C tropicalis,* Others). | *Candida* spp. | Low | + | 9,0 | 28 | 10 | The authors find that a *Candida parapsilosis* infection was associated with a lower mortality, while there was no association between *Candida tropicalis* and mortality. |
| 2019 | Bloodstream Infection Due to Vancomycin-resistant *Enterococcus* Is Associated With Increased Mortality After Hematopoietic Cell Transplantation for Acute Leukemia and Myelodysplastic Syndrome: A Multicenter, Retrospective Cohort Study | Papanicolaou, Ustun, Young et al [98] | 7128 | 2774 | 450 transplant centres worldwide | To investigate the impact of  VRE BSI within the first 100 days post HCT on overall survival (OS), nonrelapse mortality (NRM), and relapse at 1 year  following HCT | Patients who received their first allogeneic hematologic cell transplantation for acute myelogenous leukaemia (AML), acute lymphoblastic leukaemia (ALL), or myelodysplastic syndrome (MDS) between January 2008 and December 2012 | Vancomycin resistant enterococcus bloodstream infection, age (21-40, 41-50, 51-60, >60), AML/ALL intermediate, AML/ALL/MDS advanced, MDS advanced, Cord blood allograft, Human leukocyte antigen 7/8, Karnofsky performance scale <90, cytomegalovirus donor or receptor positive, hematologic cell transplant during 2010-2012 | *Enterococcus* spp. , vancomycin-resistance | Low | Indeterminable | 277.4 | Indeterminable | 10 | The overall survival model was examined here. We were unable to determine the method used for variable selection, other than that several variables were "examined" in a multivariable model. The authors conclude that VRE BSI was associated with mortality. |
| 2019 | Infections Due to Multidrug-Resistant Bacteria in Oncological Patients: Insights from a Five-Year Epidemiological and Clinical Analysis | Perdikouri, Arvaniti, Lathyris et al [37] | 73 | 22 | Greece | To describe the current epidemiology of MDR infections and identify  outcomes and risk factors associated with mortality in a cohort of patients with solid tumors | All cancer patients aged 18 years and older who presented an infection due to multi drug resistant bacteria from 1 January 2013 to 31 December 2017. | Model 1: neutropenia, C reactive protein (CRP). Model 2: creatinine, CRP. Model 3: guided antibiotics, duration of guided antibiotics. Model 4: empirical antibiotics, guided antibiotics. Model 5: CRP, guided antibiotics. Model 6: duration of empirical antibiotics, days of hospitalisation. Model 7: guided antibiotics, effective empirical antibiotics (there are 34 models overall, more models are in the Supplementary table) | *E. coli, Pseudomonas aeruginosa, Klebsiella pneumoniae, Acinetobacter baumannii, Enterococcus faecium, Staphylococcus aureus*, multidrug-, vancomycin- or oxacillin-resistant | Low | Yes | 11.0 | 37 | 2 | 34 models with each time 2 different variables included were tested. The authors selected some variables thanks to those significant in the univariate but also based on previous literature. In univariate analyses they found several associations with death, but none of these held up in multivariable analysis. |
| 2021 | Clinical Characteristics and Outcome of Bloodstream Infections in HIV-Infected Patients with Cancer and Febrile Neutropenia: A Case–Control Study | Puerta-Alcalde, Ambrosioni, Chumbita et al [119] | 180 | 43 | Spain | To compare the clinical characteristics and outcomes of bloodstream  infections (BSI) in cancer patients presenting  febrile neutropenia with and without HIV  infection, and analyze the prognostic factors for  mortality | All episodes of febrile neutropenia following chemotherapy occurring in patients with cancer and HIV from January 1997 to March 2018. | Hodgkin’s lymphoma, Myelodysplastic syndrome, Solid neoplasia, Pulmonary source, Abdominal source, Shock, Candidemia, HIV-infection. Adjusted for: chronic liver disease, diabetes mellitus, chronic renal failure, HIV, corticosteroid use, catheter-related source, inappropriate empirical antibiotic treatment, coagulase-negative staphylococci bacteremia, *S. pneumoniae* bacteremia, intensive care unit requirement. | Several bacteria and fungi, tested for resistance towards several antimicrobials | Medium | Yes | 5.4 | Indeterminable | 8 | The authors did not find any association between resistant microbes and mortality, other than candidemia, but include CoNS and S. pneumoniae as adjustment factors. |
| 2017 | Ultrasonography-­driven combination antibiotic therapy with tigecycline significantly increases survival among patients with neutropenic enterocolitis following cytarabine-­containing chemotherapy for the remission induction of acute myeloid leukemia | Pugliese, Salvatore, Iula et al [56] | 100 | 23 | Italy | To identify the predictors  of outcome within 30 days of NEC onset | Adult patients with newly diagnosed with acute myeloid leukaemia hospitalised at a haematological department from 1 January 2002 to 31 December 2012 in order to receive cytotoxic agent induction courses for haematological remission | High-dose cytarabine-containing chemotherapy, standard dose cytarabine chemotherapy, ultrasonography-driven necrotising enterocolitis NEC therapy with antibiotic regimens including tigecycline. | *Escherichia coli, Klebsiella pneumoniae, Enterococcus* spp., *Pseudomonas aeruginosa, Candida albicans* | Medium | Yes | 7.7 | 24 | 3 | It was unclear which variables were included in the final model. The authors did not find that mortality was associated with the different microbes |
| 2015 | Epidemiology and outcome of candidaemia in patients with oncological and haematological malignancies: results from a population-based surveillance in Spain | Puig-Asensio, Ruiz-Camps, Fernandez-Ruiz et al [161] | 193 | 22 | Spain | To describe the distribution and susceptibility pattern of Candida species, and to evaluate risk factors for mortality in patients  with oncological (solid tumours) and haematological malignancies | *Candida* bloodstream infection episodes in adult patients (>16 years) with underlying solid organ tumours or haematological malignancies | Charlson index, primary source of infection, catheter related source of infection, septic shock, *C. tropicalis*, central venous catheter removal within 48 hours, adequate combined treatment within 48 hours | *Candida* spp. | Low | Yes | 3.1 | 27 | 7 | The authors found that more than 1/4 of all *Candida* spp. isolates were non-susceptible to fluconazole, and that removal of the catheter and adequate antifungal treatment were associated with lower mortality. |
| 2016 | Clinical features and outcomes of Candidaemia in cancer patients: Results from Pakistan | Raza, Zafar, Mahboob et al [162] | 311 | 166 | Pakistan | To evaluate clinical risk factors and outcomes among cancer patients with candidaemia at a large cancer  treatment centre | Cancer patients with a positive blood culture for *Candida* species between January 1995 and December 2013 | Time period (1996-2005, 2006-2009, 2010-2013), Age group (18-59 year, Younger than 18 years, 60 years or older), sex, haematological malignancy (Compared to solid tumours), *Candida albicans* (Compared to non*-C.albicans* or mixed), bacteraemia, shock, receiving chemotherapy, receiving total parenteral nutrition, receiving steroids, absolute neutrophile count 500 or more, duration of stay in hospital, receiving Amphotericin B (Compared to those not receiving), empirically, after positive culture | *Candida* spp. | Low | No | 10.4 | 16 | 16 | The authors wrote that the model was developed through forward selection, but do not report a stop criterion. We were unable to find the number of deaths. The authors did not find an association between death and different *Candida* species. |
| 2017 | A fresh look at polymicrobial bloodstream infection in cancer patients | Royo-Cebrecos, Gudiol, Ardanuy et al [117] | 194 | 62 | Spain | To assess the current incidence, clinical features, risk factors, aetiology, antimicrobial resistance and outcomes of polymicrobial bloodstream infection (PBSI) in patients with cancer | Consecutive episodes of primary bloodstream infections occurring in patients with cancer, including haematopoietic stem cell transplant recipients, from January 2006 to December 2015 | Sex, age, solid tumor, MASCC risk score< 21, corticosteroid therapy, persistent bacteremia, respiratory source, septic Shock, malignancy-related complications | Several bacteria and fungi, tested for resistance towards several antimicrobials | Low | Yes | 6.9 | 11 | 9 | The authors used stepwise regression but also included clinically relevant variables in the final model regardless if they were significant or not in the univariate model. There is another model in this article with more patients analysing risk factors for polymicrobial bloodstream infections, which we did not consider to be intrinsically resistant. In the mortality model, the authors did not find that multidrug-resistant bacteria factor met the criterion in the univariable analysis to be included in the multivariable model. |
| 2017 | Characteristics, aetiology, antimicrobial resistance and outcomes of bacteraemic cholangitis in patients with solid tumours: A prospective cohort study | Royo-Cebrecos, Gudiol, Garcia et al [33] | 170 | 45 | Spain | To asses the clinical features, aetiology, antimicrobial resistance and  outcomes of bacteraemic cholangitis in patients with solid tumours (ST) | All consecutive episodes of bacteraemic cholangitis occurring in patients with solid tumours from January 2006 to September 2015 | Sex, age, pancreatic tumour, biliary tumour, corticosteroid therapy, fever >38C, malignancy-related complications | Several bacteria, tested for resistance towards several antibiotics | Low | Yes | 6.4 | 10 | 7 | The authors used stepwise regression but also included clinically relevant variables in the final model regardless if they were significant or not in the univariate model. The authors did not find an association between multidrug-resistance and mortality, and do not include it in the final, multivariable model |
| 2017 | Clinical Impact of Colonization with Multidrug-Resistant Organisms on Outcome after Autologous Stem Cell Transplantation: A Retrospective Single-Center Study | Scheich, Reinheimer, Brandt et al [51] | 184 | 45 | Germany | To assess patient outcomes after auto-HSCT in  relation to colonization with MDRGN and/or VRE and/or  MRSA | Patients admitted to a haematological and oncological department for auto-hæmatopoietic stem cell transplantation between January 2012 and October 2015, screened for multi drug resistant organisms | multi drug resistant organism colonisation (MDRO), age >55 yr, prior therapies >2, ECOG | MDRO | Medium | No | 11.3 | Indeterminable | 4.0 | The authors find an association between higher mortality and colonisation by multidrug-resistant organisms |
| 2018 | Bloodstream infections with gram-negative organisms and the impact of multidrug resistance in patients with hematological malignancies | Scheich, Weber, Reinheimer et al [62] | 109 | 25 | Germany | To analyze the impact of BSI with different gram-negative multidrug-resistant bacteria (MDRGN) compared to BSI with antibiotic susceptible gram-negative bacteria | Patients with haematological malignancies and bloodstream infection with Gram-negative rods between January 2008 and December 2016 | Sex, age > 65 years, multidrug resistant gram negative bloodstream infections (BSI), nonfermenter BSI, ANC < 500/μl duration until BSI (days), antibiotic prophylaxis, ICU admission, Charlson index > 4 points | Gram-negative bacteria, multidrug-resistance | Low | Yes | 3.1 | 18 | 8 | The authors used stepwise regression but they also included sex factor in the final model. The authors found that both being infected by a multidrug-resistant Gram-negative bacterium or a non-fermenter is associated with higher mortality |
| 2018 | Prognosis of Acute Respiratory Distress Syndrome in Patients With Hematological Malignancies | Seong, Lee, Hong et al [52] | 185 | 106 | South Korea | To investigate the clinical characteristics and outcomes of ARDS in patients with hematological malignancies  admitted to the ICU | All adult patients admitted to the Intensive care unit with haematological malignancy and acute respiratory distress syndrome between January 1, 2008, and December 31, 2015 | Initial low tidal volume ventilation, severe acute respiratory distress syndrome, carbapenem resistant gram negative bacteria (CR - GNB), age, underlying malignancies, status of disease, neutrophil count, platelet count, SOFA score, prone positioning treatment, vasopressors treatment, renal replacement therapy, gram negative bacteria, blood transfusion, pulmonary aspergillosis | Several bacteria and fungi, tested for resistance towards several antimicrobials | Medium | Yes | 8.8 | 33 | 12 | The authors used stepwise regression but also included clinically relevant variables in the final model regardless if they were significant or not in the univariate. The authors found that the mortality of patients with haematological malignancies that are moved to the ICU with ARDS was associated with carbapenem-resistant Gram-negative bacteria |
| 2019 | Risk factors for mortality due to *Acinetobacter baumannii* bacteremia in patients with hematological malignancies – a retrospective study | Shargian-Alon, Gafter-Gvili, Ben-Zvi et al [82] | 46 | 33 | Israel | To explore risk factors for short-term  mortality in patients with hematological malignancies  and carbapenem resistant *Acinetobacter baumannii*  (CRAB) bacteremia in our hospital | Hospitalised adult patients with either active haematological malignancy or past treatment for haematological malignancy in the preceding five years and Acinetobacter baumannii bacteremia between January 2010 and August 2018 | Appropriate empirical antibiotics within 48 h, SOFA score, ventilated at infection onset, urinary catheter at infection onset, duration of hospitalisation prior to bacteremia, absolute neutrophil count, creatinine level | *Acinetobacter baumannii* | Low | Yes | 4.7 | 25 | 7 | The authors found that there was a much lower mortality associated with being put on appropriate empirical antibiotic therapy within 48 hours |
| 2020 | Colonization with multi-drug-resistant organisms negatively impacts survival in patients with non-small cell lung cancer | Stratmann, Lacko, Ballo et al [34] | 295 | 31 | Germany | To determine the impact of MDRO colonization in patients  who have been diagnosed with Non-small cell lung cancer (NSCLC) who are at known high risk for invasive infections | Patients diagnosed with stage 1-4 non-small cell lung cancer between 2012 and 2016 and screened for multi drug resistant organisms | Multi drug resistant organism colonisation (MDRO), male gender, age at diagnosis, extensive diagnosis, histology: squamous cell carcinoma, histology: others, ECOG 1, ECOG 2, ECOG 3, ECOG 4, diabetes mellitus | MDRO | Low | No | 2.8 | Indeterminable | 11 | The authors concluded that the patients who were colonised by MDROs had a poorer survival than patients without such an infection |
| 2020 | Inappropriate initial antimicrobial therapy for hematological malignancies patients with Gram‑negative bloodstream infections | Tang, Wu, Cheng et al [59] | 361 | 45 | China | To determine whether  IIAT afects the prognosis in this patient population and recommend the appropriate antibiotic regimen to minimize IIAT | Patients admitted to three university-affiliated tertiary care hospitals from January 2010 to April 2015 | Age > 60 years, acute respiratory failure, disease relapsed or uncontrolled, platelet < 10 × 103 mm−3, Pitt score ≥ 4, 72-h IIAT | Gram-negative bacteria, tested for resistance towards several antibiotics | Medium | Indeterminable | 7.5 | Indeterminable | 6 | The authors did not disclose how they selected the variables to be included in the multivariable model of mortality. The authors did not find an association between intrinsically resistant pathogenic species and mortality, but found that inappropriate initial antimicrobial therapy was associated with higher mortality. |
| 2021 | Gram-Negative Bacteria Bloodstream Infections in Patients with Hematological Malignancies – The Impact of Pathogen Type and Patterns of Antibiotic Resistance: A Retrospective Cohort Study | Tang, Xu, Xiao et al [60] | 835 | 313 | China | The authors retrospectively analyzed multi-center  clinical data of patients with HMs complicated with GNBBSI over a 9 year timeframe, with the purpose of exploring the influence of different pathogen type and antibiotic  resistance patterns on prognosis of patients | Patients aged >16 years old with haematological malignancies and Gram negative bacteria-bloodstream infection, in 3 hospitals of Hunan province, from January 2010 to May 2018. | Age >60, sex, relapsed or uncontrolled malignancy, MASCC score<21, urine tube, use of vasopressors, acute respiratory failure, renal insufficiency, prior antimicrobial exposure, carbapenem resistant gram negative bacteria , non fermentative bacteria, inadequate antibiotic treatment, hemoglobin <70g/Dl, platelet <10×103mm−3, albumin <30g/L, AST >120U/L, total bilirubin >34.2μmol/L, prothrombin time >14s | Gram-negative bacteria, carbapenem resistance | Medium | Yes | 17.4 | 18 | 18 | The authors used stepwise regression but also included clinically relevant variables in the final model regardless if they were significant or not in the univariate. Authors highlighted that BSI caused by non-fermenting GNB, disease state, presence of acute respiratory failure, use of vasopressors, and inadequate antibiotic treatment were risk factors for a poor prognosis at 7 days. |
| 2016 | Bloodstream infections caused by *Klebsiella pneumoniae* in onco-hematological patients: clinical impact of carbapenem resistance in a multicentre prospective survey | Trecarichi, Pagano, Martino et al [101] | 278 | 101 | Italy | To identify risk factors for mortality in patients suffering from hematological  malignancies (HMs) with bloodstream infections (BSIs) caused by *Klebsiella pneumoniae* (KP) | All episodes of bloodstream infections caused by *K. pneumoniae* that occurred in hospitalised haematological malignancy patients | Age>55 years old, ANC<100/mmc, ANC<500/mmc, acute myeloid leukaemia, non Hodgkin's lymphoma, Hodgkin's lymphoma, haematological disease newly diagnosed / relapsed after one remission, hematologic stem cell transplant HSCT, autologous HSCT, septic shock, altered state of consciousness, acute renal failure, acute respiratory failure, acute hepatic failure, inadequate initial antimicrobial therapy, carbapenem resistant by Klebsiella pneumoniae | *Klebsiella pneumonia*, carbapenem-resistance | Medium | Yes | 6.3 | 31 | 16 | Contains one model for carbapenem-susceptible and one model for carbapenem-resistant *K. pneumoniae* infections. Finds higher mortality among patients with a resistant infection than a susceptible infection. |
| 2021 | Clinical features and outcome of *Aeromonas sobria* bacteremia in pediatric and adult patients with hematologic malignancies: A single-center retrospective study in Peru | Valcarcel, De-la-Cruz-Ku, Malpica et al [109] | 37 | 10 | Peru | To describe the clinical features and outcome  of patients with malignant hematologic diseases diagnosed with *A. sobria* bacteremia and to  identify the factors associated with survival in this population | Adult and paediatric patients with hematologic diseases and *A. sobria* bacteremia hospitalised in the National institute of Neoplastic diseases from January 2000 to December 2017 | Age group, comorbidities, qSOFA score, type of bacteremia | *A. sobria* | Low | No | 2.5 | 4 | 4 | The authors showed that organ failure and community acquired bacteremia increased the risk of mortality |
| 2018 | *Stenotrophomonas maltophilia* bacteremia and pneumonia at a tertiary-care oncology center: a review of 16 years | Velázquez-Acosta, Zarco-Márquez, Jiménez-Andrade et al [146] | 76 | 38 | Mexico | To describe the clinical characteristics and antimicrobial patterns of *Stenotrophomonas*  *maltophilia* bloodstream infections (BSI) and pneumonia episodes in patients with cancer. | Patients aged >18 years old with cancer hospitalised in Instituto Nacional de Cancerologia with *S. maltophilia* bloodstream infection or pneumonia, from January 2000 to 31 December 2016 | Model 1 in BSI: central venous catheter removal; Model 2 in pneumonia: age <65 years, received antibiotherapy with Sulfamethoxazole / trimethoprim, received appropriate treatment for *S. maltophilia* | *S. maltophilia* | Medium | Yes | 12.7 | 5 | 3 | This study contains 2 mortality models: one for BSI 30 days mortality and one for pneumonia 30 days mortality. Numbers were extracted from the pneumonia mortality model. The authors showed that for patients with pneumonia by S. maltophilia, age>65 and not having received an appropriate treatment were risk factors for higher mortality within 30 days. |
| 2015 | The ever-evolving landscape of candidaemia in patients with acute leukaemia: non-susceptibility to caspofungin and multidrug resistance are associated with increased mortality | Wang, Farmakiotis, Yang et al [107] | 67 | 28 | USA | To describe *Candida spp*. and in vitro  susceptibility patterns in a contemporary series of patients with  acute leukaemia and candidaemia, in view of the recently  updated susceptibility breakpoints. To investigate the association between in vitro resistance of *Candida spp*. to the most  commonly used antifungals and all-cause mortality rates | Patients ≥18 years of age with Acute myeloid leukaemia, Acute lymphoid leukaemia or myelodysplastic syndrome, with Candidemia from 1 January 2008 to 31 October 2012 | 14 days mortality models 1 and 2 : age >65 years old, intensive care unit stay, APACHE II score >20, mechanical ventilation, vasopressors, caspofungin non-susceptibility, multidrug resistance ; 30 days mortality models 1 and 2 : sex, age >65 years old, ICU stay, APACHE II score >20, mechanical ventilation, vasopressors, caspofungin non-susceptibility, multidrug resistance | *Candida* spp. | Medium | Yes | 4.0 | 7 | 7 | There are four models displayed in the study. Numbers were extracted from the 14 days mortality model 1: The authors showed, after multivariable analysis, association between 14 or 30 days mortality and *Candida* spp. BSI that were non-susceptible to caspofungin. However, no statistically significant association was found between mortality and removal of CVC. |
| 2020 | Infectious Complications in Patients With Multiple Myeloma After High-Dose Chemotherapy Followed by Autologous Stem CellTransplant: Nationwide Study of the Infectious Complications StudyGroup of the Polish Adult Leukemia Group | Waszczuk-Gajda, Drozd-Sokołowska, Basak et al [53] | 336 | 16 | Poland | To evaluate the epidemiology, etiology, and outcome of infections in patients with MM  undergoing ASCT in 13 Polish transplant centers | Patients with multiple myeloma having received autologous hematopoietic stem cell transplant during the 3 years period from 2012 to 2014, within one of 13 Polish transplant centres. | Bacterial multi drug resistant infection, coexistence of fungal infection, clinically documented infection | Several fungi and bacterial species, multidrug resistance | Medium | Yes | 5.3 | 9 | 3 | The authors highlighted 3 fatal outcome risk factors: MDR infection, fungal coinfection, Clinically documented infection, to adapt infection control in transplants recipients, with small sample size (16 infection related deaths). |
| 2017 | Bacteraemia due to AmpC β-lactamase-producing *Escherichia coli* in hospitalized cancer patients: risk factors, antibiotic therapy, and outcomes | Zhang, Zhang, Li et al [154] | 248 | 62 | China | To assess risk factors, antibiotic therapy, and outcomes of AmpC-EC  bacteraemia in hospitalized cancer patients | Patients aged >18 years old with solid or haematological tumour, with *E. coli* bacteremia between September 2012 and December 2015 | Abdominal infection, Urinary tract infection, unknown origin of infection, intensive care unit stay, invasive procedures, previous blood transfusion, cancer with metastasis, septic shock, organ failure, length of stay, timing of appropriate antibiotherapy | *E. coli*, AMPc Beta-Lactamase positive or negative | Low | Yes | 5.6 | 29 | 11 | The death model includes all bacteremias that were AMPc positive and negative but the study design excludes ESBL *E. coli* bacteremia. |
