# Supplementary material for: Clinical outcomes of antimicrobial resistance in cancer patients: a systematic review of multivariable models
Source: BMC Infect Dis. 2023 Apr 18;23:247. doi: 10.1186/s12879-023-08182-3 (PMC10114324; doi:10.1186/s12879-023-08182-3)
Supplement: Supplementary file 5 — Additional file 5: Table S5. All articles included in the systematic review with both an infection/colonisation outcome and a mortality outcome. [file 12879_2023_8182_MOESM5_ESM.docx]

# Supplementary material 5

**Table S5 - All included articles in the systematic review with both an infection/colonisation outcome and a mortality outcome**

| **Year** | **Title** | **Authors** | **Number of patients** | **Number of events in the final (and largest) model** | **Country/setting** | **Study aim statement** | **Patient population** | **Factors included in the final model** | **Microbial aetiology and resistance** | **Risk of bias grading (NIH tool)** | **Bivariable screening/stepwise regression** | **Events per variable in the final (and largest) model** | **Number of variables included in the tested/screened/initial model** | **Number of variables included in the final (and largest) model** | **Comments** |
| --- | --- | --- | --- | --- | --- | --- | --- | --- | --- | --- | --- | --- | --- | --- | --- |
| 2021 | The Impact of Modifying Empirical Antibiotic Therapy Based on Intestinal Colonization Status on Clinical Outcomes of Febrile Neutropenic Patients | Alrstom, Alsuliman, Daher et al [67] | 201 | 68 | Syria | To assess the intestinal colonization rate of  ESBL-E and CRE | Adults older than 18 years of age with haematological malignancies who received chemotherapy and developed febrile neutropenia | Infection model: age, gender, prior quinolone use, prior hospitalization during previous month ; Mortality model: age, sex, extended spectrum beta lactamase carriage, carbapenem resistant *Enterobacteriaceae* carriage | Extended-spectrum β-lactamase-producing *Eschericia coli* and Carbapenem-Resistant *Enterobacteriaceae* | Medium | Yes | 17.0 | 17 | 4 | The stepwise regression also includes the age and sex variables. The authors did not find that carriage of the two resistant microbes increases the risk of dying. Numbers are taken from the infection model. |
| 2018 | Bloodstream infection caused by *S.aureus* in patients with cancer:a 10-year longitudinal single-center study | Bello-Chavolla, Bahena-Lopez, Garciadiego-Fosass et al [152] | 450 | 92 | Mexico | To evaluate incidence, risk factors, clinical course, and 30-day mortality related  to *S. aureus* bloodstream infection (SABIs) in cancer patients | All positive, non-duplicate, blood cultures for *S.aureus* | Infection model: hematologic neoplasm, solid neoplasia, healthcare associated bloodstream infections (BSI), hospital acquired BSI, foci as healthcare associated pneumonia, central venous catheter (CVC), neutropenia, gram negative bacteria coinfection, gram positive bacteria coinfection, inpatient management, specific anti SA treatment, CVC removal, days from infection to CVC removal, intensive care admission, septic shock, relapse after *S. aureus* bacteremia. Mortality model: abdominal source, hematologic malignancy, Methicillin-resistant *Staphylococcus aureus* (MRSA), glucose > 140 mg/dL, catheter removal, anti-staph treatment < 48 h, infectious endocarditis | Methicillin-resistant *Staphylococcus aureus* | Medium | Yes | 5.8 | 28 | 16 | Authors created a risk stratification based on the regression coefficients from the mortality model. The final model table can be found in supplementary material. The final model is also adjusted for CCI and glycemia >140 mg/dL. Numbers extracted from the infection model of MRSA infection in hematologic malignancies. |
| 2019 | Carbapenem versus Cefepime or Piperacillin-Tazobactam for Empiric Treatment of Bacteremia Due to Extended-Spectrum-β-Lactamase-Producing *Escherichia coli* in Patients with Hematologic Malignancy | Benanti, Brown, Shigle et al [103] | 103 | 8 | USA | To compare mortality in patients with hematologic  malignancy and ESBL-producing *E. coli* bacteremia treated empirically with carbapenems or the potential carbapenem-sparing alternative cefepime or piperacillintazobactam. To compare other clinically relevant outcomes  achieved with these agents, including the persistence of bacteremia and fever | Patients with hematologic malignancy and a first episode of mono-microbial extended spectrum beta lactamase-producing *E. coli* bacteremia | Infection model: cefepime treatment, piperacillin-tazobactam treatment (all vs Carbapenem treatment), age. Mortality model: cefepime treatment, Pitt bacteremia score, intensive care unit residence | Extended-Spectrum-β-Lactamase-Producing *Escherichia coli* | Medium | Yes | 4.0 | 11 | 2 | The stepwise regression included the cefepime treatment variable. The infection outcome is persistent bacteraemia. Authors found that only Pitt bacteraemia score is associated with an increased mortality. Numbers were extracted from 14 days mortality model |
| 2019 | Associated factors and clinical outcomes of bloodstream infection due to extended-spectrum β-lactamase-producing *Escherichia coli* and *Klebsiella pneumoniae* during febrile neutropenia | Ben-Chetrit, Eldaim, Bar-Meir et al [126] | 80 | 80 | Israel | To evaluate factors associated with  ESBL-positive bacteremia among neutropenic patients, as well as  its impact on clinical outcome | All patients with hematologic or oncologic (solid tumours) diseases who were diagnosed with extended spectrum beta lactamase (ESBL) -positive and ESBL-negative *Escherichia coli* or *Klebsiella pneumoniae* bacteremia during febrile neutropenia between January 2010 and October 2017 | Infection model: pathogene type, fungal infection within 30 days, presence of central venous catheter long term during index culture, admission days prior index culture, index culture >48h, previous antimicrobial therapy (6 months). Mortality model: extended spectrum beta lactamase-positive bacteremia, appropriate empirical antimicrobial therapy, charlson comorbidity index (CCI) >4, Pitt bacteremia score ≥4, underlying disease. | Extended-spectrum β-lactamase-producing *Escherichia coli* and *Klebsiella pneumoniae* | Low | Yes | 16.0 | 25 | 5 | Authors found that a central venous catheter and previous antibiotic use is associated with ESBL-positive Gram-negative bacteraemia, but that the ESBL-positivity is not associated with an increase in mortality. Both models are the same size hence we chose the infection model to extract the data : extended-spectrumβ-lactamase(ESBL)-positive Gram-negative bacteremia. |
| 2018 | Risk, Outcomes, and Predictors of *Clostridium difficile* Infection in Lymphoma: A Nationwide Study | Bhandari, Pandey, Dahal et al [79] | 236312 | 19358 | USA | To determine the  risk of CDI in hospitalizations with lymphoma along with its trend, outcomes, and predictors | All adult patients aged 18 years or older with the primary diagnosis of lymphoma between 2007 and 2011 | Age, sex, race, Hodgkin lymphoma, hematologic stem cell transplant, chemotherapy, radiotherapy, gastro intestinal surgery, infection, GVHD, IBD, CCI, year of admission. Mortality: *C. difficile* infection. | *Clostridioides difficile* | High | No | 19358.0 | Indeterminable | 1 | Authors found that HSCT, chemotherapy and surgery in the gut is associated with CDI, which increases the risk of death. Numbers were extracted from the mortality model. Most of the dataset is simulated using weights to create national estimates. |
| 2018 | Risk factors for bloodstream infections due to extended-spectrum β-lactamase producing *Enterobacteriaceae* in cancer patients | Ceken, Iskender, Gedik et al [127] | 122 | 31 | Turkey | To identify the risk factors and outcomes related to BSIs caused by extended-spectrum beta-lactamase  (ESBL)-producing *Enterobacteriaceae* in cancer patients | Haematology/oncology patients with culture positive bloodstream infections with *E. coli* or *Klebsiella* spp from January 2013 to December 2014 | Infection model: Quinolone prophylaxis, piperacillin-tazobactam previous 90 days, carbapenem previous 90 days, GNB infection previous 3 months and total parenteral nutrition. Mortality model : neutropenia, duration of neutropenia before infection, inappropriate treatment, delay for appropriate treatment, complications (hypoxia, septic shock etc.). | Extended-spectrum β-lactamase producing *Eschericia coli* and *Klebsiella* spp. | Medium | Yes | 10.3 | 4 | 3 | It is not clear whether the authors used p<0.05 to include the variables in the multivariable models. Numbers were extracted from the 30 days mortality model. For the infection model, authors found out that quinolone prophylaxis, total parenteral nutrition, piperacillin or carbapenem for the previous 90 days, or previous infection with GNB previous 90 days were risk factors to develop ESBL-P BSIs. Authors found that two factors were associated with an increased mortality in BSI cancer patients - prolonged neutropenia and complications. |
| 2017 | Poorer outcomes among cancer patients diagnosed with *Clostridium difficile* infections in United States community hospitals | Delgado, Reveles, Cabello et al [138] | 30244426 | Indeterminable | USA | To describe CDI incidence and health outcomes nationally among cancer patients in  the United States | Patients ≥18 years old with a principal or secondary ICD-9-CM code (discharge diagnosis of cancer) obtained from the U.S. National Hospital Discharge Surveys from 2001 to 2010 | Indeterminable | *Clostridioides difficile* | High | Indeterminable | Indeterminable | Indeterminable | Indeterminable | Authors found that CDI significantly increased the risk for mortality and prolonged hospital stays among cancer patients. But overall the authors do not give any details about the model which was computed here. |
| 2018 | Role of Lock Therapy for Long-Term Catheter-Related Infections by Multidrug-Resistant Bacteria | Freire, Pierotti, Zerati et al [112] | 212 | 75 | Brazil | To analyze outcomes in cancer patients with LTCVC associated infection, identify risks for unfavorable outcomes, and determine the  impact of MDR bacteria and antibiotic lock therapy (ALT) in managing such infections | All long-term central venous catheter (LTCVC)-associated infections diagnosed at the a cancer institute between January 2009 and December 2016 | Haematological malignancies, type of CT last 3 months, palliative care, ICU admission, hemodynamic instability, SOFA score, CVC duration, *A.baumanii*, polymicrobial infection, Nadir WBC count, adherence to protocol, antibiotic lock therapy | Several bacteria and fungi, tested for resistance towards several antibiotics | Low | Yes | 6.3 | 28 | 12 | Model of relapse and mortality model. Numbers were extracted from the treatment failure or relapse model. The authors found that ALT is a protective factor for relapse risk and that there is an increased hazard of death when the patient is infected by multidrug-resistant bacteria, but that antibiotic lock therapy is associated with a decrease in death. |
| 2018 | Gut Colonization with Carbapenem-resistant *Enterobacteriaceae* Adversely Impacts  the Outcome in Patients with Hematological Malignancies: Results of A Prospective  Surveillance Study | Jaiswal, Gupta, Kumar et al [68] | 225 | 48 | India | To evaluate the prevalence of  colonisation with CRE in patients with  haematological malignancies and its impact on the  outcome of the patients undergoing treatment for  these disorders | Patients with newly diagnosed haematological malignancy admitted from October 2013 to January 2016 | Age, gender, acute leukaemia, acute myeloid leukaemia, Charlson comorbidity index, performance status | *Enterobacteriaceae*, carbapenem-resistance | Low | No | 8.0 | 6 | 6 | The authors concluded that there is an association between having acute leukaemia and being at risk of both contracting and dying to a CRE infection. Numbers were extracted from the model for risk to be colonised to CRE before hospital admission. |
| 2015 | Outcomes of *Clostridium difficile* Infection in Hospitalized Leukemia Patients: A Nationwide Analysis | Luo, Greenberg, Stone [80] | 1243107 | 95355 | USA | To determine the  trend of incidence of CDI in hospitalized leukemia patients, to  evaluate the impact of CDI on mortality and expense, and to  identify the risk factors for developing CDI in this population | Adults, aged ≥18 years, with a primary diagnosis code for leukaemia | Infection model: age, gender, race (Caucasian, Black, Hispanic, Asian/Pacific Islander, Native American), length of stay (days: 2, 3–5, 6–10, 11–15, ≥16), hospital teaching status, leukaemia type, complications (several), Charlson index (0, 1, 2, 3, 4, >5). Mortality model: age <65, gender, race, Charlson index score, teaching status sof hospital, the presence of complications or comorbidities (eg, hypertension, diabetes,pneumonia, etc.), and the performance of bone marrow vs stem cell transplant during hospitalisation. | *Clostridioides difficile* | High | No | 13622.1 | 7 | 7 | The authors found a number of demographic or clinical associations with CDI, and that CDI was also associated with mortality. It is not reported how much of the dataset is individual data or projections based on weights. Numbers were extracted from the mortality model because no details are given concerning the other infection model. |
| 2017 | Carbapenem-resistant *Klebsiella pneumoniae* in high-risk haematological patients: factors favouring spread, risk factors and outcome of carbapenem-resistant *Klebsiella pneumoniae* bacteremias | Micozzi, Gentile, Minotti et al [102] | 19 | 10 | Italy | To identify conditions favouring  CRKP spread in a haematological unit, assess risk factors for  bacteremia in haematological patients colonized with CRKP,  and analyse risk factors for poor outcome among haematological patients with CRKP bacteremia. | All patients with haematological malignancies infected or colonised with carbapenem resistant *K. pneumoniae* (CRKP), hospitalised between 24 February 2012 and 31 May 2013 | Infection model: underlying disease, age, gender, intensive chemotherapy, >= 7 days colonised by carbapenem resistant *K. pneumoniae*. Mortality model 1: *Klebsiella* producing carbapenemase-*K.pneumoniae* bloodstream infection developing during inactive antibiotic treatment. Mortality model 2 and 3: initial adequate therapy, breakthrough bacteremia occurrence within 48 h of ongoing antibiotics, intensive Chemotherapy. | *Klebsiella pneumoniae*, carbapenem-resistance | Low | Yes | 3.3 | 19 | 3 | The infection outcome is bacteraemia. The authors found an association between AML and CRKP bacteraemia, and further that there was an association between survival and initial adequate therapy. Data was extracted from the final mortality model. |
| 2015 | Bacteraemia caused by *Escherichia coli* in cancer patients at a specialist center in Pakistan | Parveen, Sultan, Raza et al [155] | 227 | 80 | Pakistan | To analyse the antimicrobial susceptibility patterns of *Escherichia coli* bacteraemia among cancer  patients, and to assess the risk factors and outcomes of multidrug-resistant *Escherichia coli* bacteraemia | Cancer patients with *E.coli* bacteraemia during the 12 months from December 2012 to November 2013. | Infection model: sex, age less than 18 years, haematological malignancy, hospitalization within 30 days prior to infection, intensive care unit admission, Charlson score, central venous catheter use, previous chemotherapy within 30 days, previous surgery within 30 days, previous radiation within 30 days, absolute neutrophil count (ANC)less than 100 cells/mm3, use of quinolones within 90 days of index sampling, use of third generation cephalosporins within 90 days of index sampling, use of Piperacilin/Tazobactam within 90 days of index sampling, use of carbapenems within 90 days of index sampling. Mortality model: sex, age less than 18 years, haematological malignancy, hospitalisation within 30 days prior to infection, admission to intensive care unit, previous chemotherapy within 30 days, previous surgery within 30 days, previous radiation within 30 days, ANC less than 100 cells/mm³, Charlson score, multi drug resistant *E.coli*, inappropriate empiric antibiotic therapy. | *Escherichia coli*, multidrug-resistant | Low | No | 6.7 | 12 | 12 | The infection outcome is MDR in *E. coli* bacteraemia. The authors concluded that MDR *E. coli* is not associated with an increase in mortality. Numbers were extracted from the mortality model. |
| 2020 | *Candida* spp bloodstream infections in a Latin American Pediatric Oncology Reference Center: Epidemiology and associated factors | Paixao de Sousa da Silva, de Moraes-Pinto, Teofilo Pignati et al [160] | 90 | 90 | Brazil | To characterize *Candida spp* bloodstream infections (BSI) in a reference  centre for paediatric oncology and to describe the most prevalent risk factors associated with candida infections | Paediatric patients admitted to a paediatric oncology institute who presented with *Candida* spp. BSI from January 2004 to December 2016. | Model of treatment failure or infection model: age, hospitalisation, corticosteroid, prior antifungal use, skin lesion, dissemination, *Candida* spp Species, *C. tropicalis*, treatment. 30 days Mortality model: Age, Hospitalisation, intensive care unit, corticosteroid, prior antifungal use, skin lesion, dissemination, *Candida* spp Species, *C. parapsilosis* | *Candida* spp. | Low | Yes | 10.0 | 31 | 9 | There are 2 models in this study, one for antifungal treatment failure (then an infection model), the other one for 30 days mortality. Numbers were extracted from the infection model. The authors found that older age and thrombocytopenia were good independent predictors of therapeutic failure and that *Candida parapsilosis* infection was associated with a lower mortality, while there was no association between *Candida tropicalis* and mortality. |
| 2017 | Epidemiology, risk factors, and outcomes of infections in patients undergoing liver transplantation for hilar cholangiocarcinoma | Ramanan, Cummins, Wilhelm et al [35] | 124 | 255 | USA | To describe the incidence, types, and risk factors of post-transplant  infections in LT patients with CCA. | Adult patients who underwent liver transplantation for biopsy-proven hilar cholangiocarcinoma at a clinic between January 2004 and March 2013 | Infection model: vancomycin resistant enterococcus (VRE) colonisation pre-transplant, living donor transplant, cold ischemia time, cytomegalovirus donor seropositivity, hepatic artery thrombosis post- transplant, biliary stricture post- transplant, intra-abdominal fluid collection post-transplant, number of re- operations in 1st month post- transplant. Mortality model: abdominal infection, recurrent Hilar cholangiocarcinoma , Post-transplant *C. difficile* infection, Bloodstream infection | Several bacteria and fungi, tested for resistance towards several antimicrobials | Medium | No | 15.9 | 16 | 16 | There are two more models with an infection outcome in the article, of abdominal infection and bloodstream infection, but the model of any post-transplant infection is included here. The authors modelled subsets of 10 variables for infections and 2 variables for death and selected the best model based on score. They found that infections are associated with increased mortality, and that VRE colonisation pre-transplant is associated with infection post-transplant. Numbers were extracted from the infection model. |
| 2016 | *Clostridium difficile* Infections amongst Patients with Haematological Malignancies: A Data Linkage Study | Selvey, Slimings, Joske et al [81] | 2085 | 65 | Australia | To identify risk factors for *Clostridium difficile* infection (CDI) and assess CDI outcomes among  Australian patients with a haematological malignancy | Patients admitted at least once to any hospital in the region for treatment or management of a haematological malignancy in the period 1 July 2011 to 30 June 2012 | Infection model: acute lymphocytic leukaemia not in remission, neutropenia at any time, any bacterial pneumonia, other bacterial infection, any bacterial pneumonia and neutropenia at any time. Mortality model: acute myeloid leukaemia not in remission, *C. difficile* infection, age 65 and over, cerebrovascular disease, dementia, neutropenia at any time. | *Clostridioides difficile* | Low | Yes | 4.1 | 30 | 16 | There are two infection models, one with all patients and one with patients from October 2011. There are also two mortality models, one with death within 60 days and one with death within 90 days. The authors found that a *C. difficile* infection is associated with an increased mortality. Univariate analysis included both table 1 and 2. It is unclear where the dementia variable was taken from, but it is included in the mortality model. Numbers were extracted from the CDI model in all patients |
| 2020 | Risk factors for and clinical outcomes of carbapenem non-susceptible gram negative bacilli bacteremia in patients with acute myelogenous leukemia | Shin, Shin, Kang et al [61] | 489 | 63 | South Korea | To explore the risk factors for carba-NS  GNB bacteremia and its clinical outcomes in patients  that underwent induction or consolidation chemotherapy  for AML | Adult patients (aged ≥18 years) with acute myeloid leukaemia that contracted gram negative bacteremia during induction or consolidation chemotherapy, from January 2000 to December 2015 | Infection model: age, chemotherapy, isolation of VRE in the prior 1 year, isolation of ESBL in the prior 1 year, isolation of carbapenem resistant *A. baumanii* (CRAB) in the prior 1 year, presence of preceding bacteremia during the hospitalisation, central line associated infection, *E.coli*, *Klebsiella* spp.,  *Pseudomonas* spp, *S. maltophilia*, *Acinetobacter* spp, antibiotics at the onset of gram negative bacteria (GNB) bacteremia, inappropriate empiric antibiotics, days from chemotherapy to GNB bacteremia, hospital days to GNB bacteremia. Mortality model: age, mean (± SD), induction or re-induction chemotherapy, isolation of vancomycin resistant enterobacteriaceae (VRE)in the prior 1 year, isolation of extended spectrum beta lactamase (ESBL) in the prior 1 year, isolation of CRAB in the prior 1 year, history of GNB bacteremia in the prior 1 year, presence of preceding bacteremia during the hospitalisation, pneumonia, Pitt score, septic shock, carba-non sensitive, inappropriate empiric antibiotics, days from chemotherapy to GNB bacteremia, hospital days to GNB bacteremia. | Gram-negative bacilli, carbapenem-resistant | Medium | + | 4.5 | 22 | 14 | The authors found that carbapenem treatment at the onset of Gram-negative bacteraemia and infection with carbapenem-resistant *Acinetobacter baumannii* was associated with a carbapenem-resistant Gram-negative bacteraemia, and also that infection with VRE the previous year and carbapenem-resistance was associated with an increased mortality in patients with Gram-negative bacteraemia. Numbers were extracted from the mortality model. |
| 2019 | Trends in Incidence and Outcomes of *Clostridium difficile* Colitis in Hospitalized Patients of Febrile Neutropenia A Nationwide Analysis | Siddiqui, Khan, Khan et al [139] | 394987 | Indeterminable | USA | To assess the risk factors for development  and mortality of CDI in patients with FN | All patients with primary and secondary diagnosis of febrile neutropenia, with and without *C. difficile* infection from years 2008 to 2014, according to the Nationwide Inpatient Sample (NIS) | Age, race, gender, Charleston Comorbidity Index, malignancy type, admission days, hospital region, hospital location, sepsis | *Clostridioides difficile* | High | No | Indeterminable | 23 | 23 | Authors showed that patients older than 65 years old,Hispanic race, and presence of sepsis were associated with increased risk of mortality in cancer patients with FN. In addition, Hispanic race, haematological malignancies, and sepsis were significant risk factors for development of CDI in these patients. Both models are the same size. |
| 2019 | Increase in *Candida Parapsilosis* Candidemia in Cancer Patients | Sun, Chen, Xiao et al [163] | 323 | 47 | China | To identify the risk factors of candidemia and asses possible clinically  significant differences between *Candida parapsilosis* and other Candida species in a Chinese  tertiary cancer center over six years | All patients with positive blood culture for *Candida* species at a cancer hospital from 1 March 2012 to 28 February 2018 | Infection by *C. parapsilosis* model: age, hematologic malignancy, in intensive care unit (ICU) at diagnosis, parenteral nutrition, neutropenia, previous surgery last 3 months, abdominal surgery, chemotherapy, previous antifungal use, duration of antifungal; Infection by *C. albican*s model: age, hematologic malignancy, ICU at diagnosis, neutropenia, abdominal surgery, central venous catheter (CVC) related candidemia, chemotherapy, previous antifungal use, duration of antifungal; Mortality model: age, ICU at diagnosis, mechanical ventilation, CVC related candidemia, removal of CVC. | *Candida* spp. | Medium | Yes | 5.2 | 23 | 9 | The authors concluded that there are several risk factors associated with a *Candida* non-albicans infection, but did not model the role of these infections in the mortality of patients. To compute the number of events per variable we chose the infection model by *C. parapsilosis*. |
| 2017 | *Pseudomonas aeruginosa* bacteraemia in patients with hematologic malignancies: risk factors, treatment and outcome | Tofas, Samarkos, Piperaki et al [83] | 64 | 23 | Greece | To identify factors associated with *Pseudomonas aeruginosa* (PA) bloodstream infection (BSI) in patients with hematological malignancies and evaluate the outcome of the affected patients | Cases: Consecutive patients with hematologic malignancy who developed *P. aeruginosa* (PA) bloodstream infection (BSI) between January 2012 and December 2014. Controls: Patients with hematologic malignancies without PA infection. | Infection with *P. aeruginosa* model: severity of underlying disease (Ultimately fatal, Rapidly fatal), hospitalisation in preceding 3 months, surgery in preceding 3 months, neutropenia, use of steroids, Prior use of any antimicrobial, prior use of anti-pseudomonal lactams including carbapenems, prior use of Aminoglycoside, prior use of Fluoroquinolones. Infection with carbapenem-resistant PA model: hospitalization in preceding 3 months, anti-pseudomonal lactams, carbapenems, aminoglycoside, fluoroquinolones. Mortality model : severity of sepsis (Sepsis, Severe sepsis or septic shock), empirical treatment (No active drug, At least one active drug), definitive treatment (One active drug, More than one active drugs). | *Pseudomonas aeruginosa*, carbapenem resistant | Low | Yes | 7.7 | 19 | 3 | There are three models here, one for PA BSI, one for carbapenem-resistant PA BSI and one for mortality in patients with PA BSI. All numbers are extracted from the 28 days mortality model. The authors found that prior use of antibiotics were associated with PA BSI, and that prior hospitalisation was associated with carbapenem-resistance. |
| 2021 | Oral fluoroquinolones for definitive treatment of gram-negative bacteremia in cancer patients | Tossey, El Boghdadly, Reed et al [122] | 211 | 39 | USA | To compare the outcomes of cancer patients  transitioned from IV to oral (PO) therapy compared to continuation of IV treatment | Patients at a cancer centre with positive blood cultures for gram-negative organisms during the study period from November 1, 2011, to September 30, 2017 | Intravenous group, hematologic malignancy, neutropenia, *P. aeruginosa* infection, history of hematologic stem cell transplantation, Pitt bacteremia score, intensive care unit admission | Gram-negative bacteria | Medium | Indeterminable | 5.6 | Indeterminable | 7 | The authors modelled both infection recurrence and mortality as a composite outcome "treatment failure". No method for variable selection is disclosed. They found that having a *P. aeruginosa* infection is associated with this composite outcome. |
| 2019 | Bloodstream infections caused by Escherichia coli in onco-haematological patients: Risk factors and mortality in an Italian prospective survey | Trecarichi, Giuliano, Cattaneo et al [104] | 342 | 24 | Italy | To identify risk factors for 3GC resistance by EC isolates and prognostic factors, including the impact of 3GC resistance, in HM  patients with BSIs caused by EC | All episodes of bloodstream infection caused by E. coli (EC BSI) occurring in hospitalised haematologic malignancies adult patients from January 2016 to December 2017 in 15 haematology wards | Infection model: recent endoscopic procedures, parenteral nutrition, polymorphonuclear leukocytes < 500/mmc for at least 10 days, previous antibiotic therapy, multiple myeloma, allogeneic matched unrelated transplant, multi drug resistant bacteria culture-positive surveillance rectal swabs, antibiotic prophylaxis with fluoroquinolones. Mortality model: sex, complete remission, autologous transplant, septic shock, altered consciousness, acute renal failure, avute respiratory failure, acute hepatic failure, 3rd generation cephalosporins resistance by *E. coli* isolate. | *Escherichia coli*, 3rd generation cephalosporin-resistant | Medium | Yes | 2.7 | 34 | 9 | The infection outcome here is resistance towards 3rd generation cephalosporins in *E. coli* bacteraemia. The authors found that such infections are associated with an increased mortality in these patients. All the numbers were extracted from the mortality model |
| 2019 | Clinical outcome of Escherichia coli bloodstream  infection in cancer patients with/without biofilm  formation: a single-center retrospective study | Zhang, Gao, Li et al [156] | 324 | 71 | China | To investigate the impact  of BF-positive, EC-caused BSI on the clinical outcome of  hospitalized cancer patients | Adult patients (aged ≥18 years) with cancer and first episode of E. coli bloodstream infection hospitalised at Tianjin Cancer Institute from January 2013 to September 2017 | Infection models 1 (extended spectrum beta lactamase yes no) and 2 (biofilm formation yes no) : sex, age, hypertension, chronic heart disease, diabetes mellitus, solid tumor, hematological cancer, abdominal infection, urinary tract infection, biliary infection, pulmonary infection, catheter-related infection, unknown origin infection, others, C3G (or cephalosporin (model1) exposure previous month, betalactam with or without inhibitor of betalactamase exposure previous month, carbapanems exposure previous month, combined exposure previous month, aminoglycoside exposure previous month, invasive procedure, surgery previous month, chemotherapy, ESBL, LOS (model 1). Mortality model: sex, age, hypertension, chronic heart disease, diabetes mellitus, solid tumor, hematological cancer, LOS, ICU, invasive procedure, mechanical ventilation, metastasis, chemotherapy, surgery, previous blood transfusion, ESBL, BF, organ failure and sepsis shock | ESBL-producing *Escherichia coli* or biofilm-producer *Escherichia coli* | Low | Yes | 8.9 | 18 | 8 | The authors concluded biofilm is a risk factor for mortality , and also to be ESBL or not (and vice versa). No clear association between infection with biofilm-producer and antibiotics used. Numbers were extracted from the mortality model. |
| 2020 | Risk Factors and Outcomes of Antibiotic-resistant  Pseudomonas aeruginosa Bloodstream Infection in Adult  Patients With Acute Leukemia | Zhao, Lin, Liu et al [84] | 293 | 293 | China | To evaluate clinical factors associated with the outcomes  of PA BSI and identify factors associated with the development  of PA BSI caused by carbapenem-resistant (CR) or multidrugresistant (MDR) strains | Adult patients hospitalised with Acute Leukaemia and P. aeruginosa bloodstream infection from January 2014 to December 2019 | Infection model 1 : Previous use of fluoroquinolones, previous use of piperacillin- tazobactam, previous use of carbapenems, HSCT. Infection model 2 : Previous use of fluoroquinolones, previous use of piperacillin- tazobactam. Death model : Age>55, Perianal infection, Pulmonary infection, MDR-PA | *P. aeruginosa*, carbapenem resistant or MDR | Medium | + | 48,8 | 14 | 6 | Outcomes for infection are 1) Carbapenem-resistant *P. aeruginosa* infection or 2) MDR *P. aeruginosa* infection. Variables with p<0.1 in the univariate analysis were included in the multivariable model. The authors found that MDR is associated with bad prognosis in BSI *P. aeruginosa* patients, and they have tried to show as well that exposure to certain classes of antibiotics could lead to developing MDR *P. aeruginosa* BSI in these patients. They aimed to enhance the role of an appropriate antibioprophylaxis in acute leukaemia patients. Numbers were taken from infection model MDR PA BSI. |
