# Supplementary material for: Clinical outcomes of antimicrobial resistance in cancer patients: a systematic review of multivariable models
Source: BMC Infect Dis. 2023 Apr 18;23:247. doi: 10.1186/s12879-023-08182-3 (PMC10114324; doi:10.1186/s12879-023-08182-3)
Supplement: Supplementary file 6 — Additional file 6: Table S6. All risk factors investigated in the final, multivariable model of the included studies, and their respective categories. [file 12879_2023_8182_MOESM6_ESM.docx]

# Supplementary material 6

**Table S6 - All risk factors investigated in the final, multivariable model of the included studies, and their respective categories.**

| **Infection/colonisation** |  |
| --- | --- |
| **Category** | **Included risk factors** |
| Antibiotic use | Antibiotic use, Mean length of CDI treatment, CDI treatment failure, Ciprofloxacin, Levofloxacin, Meropenem, Antipseudomonal penicillin, Cumulative glycopeptide exposure, Susceptibility to applied perioperative antibiotics, β-lactam/β-lactamase inhibitor, Cephalosporin, Metronidazole, Previous antibiotic therapy, Prior antibiotic use, Days of empiric antibiotics, Cycling vs carbapenem period, Days of carbapenem, Days of cefepime or piperacillin/tazobactam, Antibiotics prior 3 mo, Antibiotics (no.), Antibiotics (d), Antibiotic therapy within 30 days, Prior piperacillin-tazobactam therapy (within 1 mo), Prior fluoroquinolone prophylaxis (within 1 mo), Prior antipseudomonal carbapenem therapy (within 1 mo), Antibiotic days of therapy, Vancomycin IV, Cephalosporin, Previous azole exposure history, Daptomycin, Cefepime received within 90 days, Cumulative Daptomycin days within 90 days, Cumulative Fluoroquinolone days within 90 days, Usage of quinolones inside 95 days of catalogue sample, Usage of third group cephalosporins inside 95 days of index sample, Usage of Piperacilin/Tazobactam within 95 days of index sampling, Usage of carbapenems inside 95 days of index sample, No. of previous antibiotic use ≥3, Previous TMP/SMX use, Antibiotic prophylaxis (vs. G-CSF support), Prior use of extended-spectrum cephalosporin, Prior use of glycopeptides, Antibiotic use within 30 days before transplantation (No antibiotic, Any antibiotic except TMP-SMX, TMP-SMX), The total length of antibiotic administration for more than 5 days before diagnosis of anastomosis site leakage. Ajustment variables indeterminable., Days of antibiotics received, Antibiotic use previous 90 days (Antibiotic therapy >14 days), Specific antibiotics previous 90 days (PTZ, Vancomycin), Beta-lactams during the previous month, Oral vancomycin prophylaxis, prophylactic antibiotherapy received, treatment antibiotic duration, Antibiotic exposure, b-lactam/b-lactamase inhibitor within previous 30 days, Carbapenem within previous 30 days, Receiving TMP-SMX at BSI onset, Receiving cephalosporin at BSI onset, Receiving fluoroquinolone at BSI onset, Antibiotic exposure (ceftriaxone, fluoroquinolones, piperacillin/tazobactam, other (daptomycin), other (vancomycin IV), number of antibiotic exposures), Prophylactic antibiotic, IV antibiotics in previous 90 day, Concurrent antibiotics use for PCN infection, antifungals, Antibiotic exposure prior to CDI, non betalactam antibiotic previous exposure, Carbapenem previous exposure, empirical treatment of EB-BSI with carbapenems, empirical treatment with antibiotics combination, prior quinolone use, specific anti SA treatment, cefepime treatment, piperacillin-tazobactam treatment (all vs Carbapenem treatment), previous antimicrobial therapy (6 months)., Quinolone prophylaxis, piperacilline-tazobactam previous 90 days, carbapenem previous 90 days, use of quinolones within 90 days of index sampling, use of third generation cephalosporins within 90 days of index sampling, use of Piperacilin/Tazobactam within 90 days of index sampling, use of carbapenems within 90 days of index sampling., prior antifungal use, vancomycin resistant enterococcus (VRE) colonization pre-transplant, isolation of carbapenem resisant A. baumanii (CRAB) in the prior 1 year, antibiotics at the onset of gram negative bacteria (GNB) bacteremia, inappropriate empiric antibiotics, previous antifungal use, duration of antifungal, previous antifungal use, duration of antifungal, Prior use of any antimicrobial, prior use of anti-pseudomonal lactams including carbapenems, prior use of Aminoglycoside, prior use of Fluoroquinolones. Infection with carbapenem-resistant PA model: hospitalization in preceding 3 months, anti-pseudomonal lactams, carbapenems, aminoglycoside, fluoroquinolones, previous antibiotic therapy, antibiotic prophylaxis with fluoroquinolones, C3G (or cephalosporin (model1) exposure previous month, betalactam with or without inhibitor of betalactamase exposure previous month, carbapanems exposure previous month, combined exposure previous month, aminoglycoside exposure previous month, previous use of fluoroquinolones, previous use of piperacilline- tazobactam, previous use of carbapenems, Previous use of fluoroquinolones, previous use of cephalosporines, previous use of piperacilline- tazobactam, previous use of carbapenems |
| Basic characteristics | Age, BMI, Male sex, Age, Age, Age, Male (vs. female), Gender, Age, Age, Age, Age, Gender female, ESBL-PE. Adjusted for the following confounders: age, sex, Age (≥60 years vs <60), Gender (male), Asian race, Man sex, Age fewer than 19 years, Age (per ten years), Hospitalization during previous year, Gender, Race, Age, Year, Age categories, Race/Ethnicity, Female, Obesity, Median household income in quartile., Age at diagnosis, Age (>65), Smoking history (+ vs -), Sex, Age (<60 vs >60), BMI, age, gender, age., Age, sex, race, year of admission., Age, gender, age, gender, race , age, gender, sex, age less than 18 years, age, age, Age, race, gender, age, age, sex, age, sex |
| Cancer-related | Hematological malignancy, Tumour stage, Tumour localisation, Hematologic malignancy, AML, Karnofsky performance Status, and type of malignancy., HCI-CI (≥3 vs 0- 2), KPS (>80 vs ≤80), Stage of malignancy, Hematologic malignancy, Hematology/oncology model: Stage of disease (Hematologic malignancy–newly diagnosed, Hematologic malignancy–continuation/consolidation), Neoplasia in remission, Type of Malignancy, Gastrointestinal cancer, Breast cancer, Lung cancer, Prior CRE at any site within the previous 90 days, salvage lymphoma, salvage lymphoma), Leukemia, Lymphomia, Site (Colon vs Rectum), Morphology (Ulcerative vs Exophytic), Differentiation (Poor vs Well), T stage (Non-T4 vs T4), N stage (LNM neg. Vs LN pos.), hematological neoplasm, solid neoplasia, Hodgkin lymphoma, acute leukemia, acute myeloid leukemia, leukemia type, haematological malignancy, acute lymphocytic leukaemia not in remission, malignancy type, hematologic malignancy, hematologic malignancy, severity of underlying disease (Ultimately fatal, Rapidly fatal), hematologic malignancy, multiple myeloma, solid tumor, hematological cancer |
| Chemotherapy/immunosuppressants | Immunosuppressant use, Conditioning regimen, Chemotherapy dose modifications, Intervall chemotherapy to diarrhea, Administration of chemotherapy, Conditioning (RIC vs MAC), Number induction chemotherapy regimens, Clofarabine within 30 days, Previous chemotherapy inside 1 month, No prophylaxis (vs. G-CSF support), ≥VGPR before ASCT (vs. ≤PR), Oncology treatment previous 90 days (Rituximab, Clofarabine)., Immunosuppressive therapy, No chemotherapy, Early de-escalation vs hematopoietic recovery de-escalation, chemotherapy exposure, decitabine, CHOP, Chemotherapy delay OR and mean difference., chemotherapy, intensive chemotherapy, previous chemotherapy within 30 days, chemotherapy, chemotherapy, chemotherapy, chemotherapy |
| Comorbidities/clinical condition | Comorbidities, Diabetes mellitus, Pleural effusion, Hematologic diseases, Diabetes, Hematological disease, Congestive heart failure, Haematological distortion, Carlson score, Diabetes mellitus, Peripheral vascular disease, Chronic renal disease, Atrial fibrillation, Congestive heart failure, Hypertension, Chronic liver failure, Elixhauser score, Renal disease, Chronic renal diseases, Gastroenteritis diseases, CCI, Charlson comorbidity index, Charlson index, underlying disease, Charlson score, cold ischemia time, Charleston Co-Morbidity Index, severity of underlying disease (Ultimately fatal, hypertension, chronic heart disease, diabetes mellitus |
| Gastrointestinal | Gastrointestinal GVHD, Number of stools/day, Preoperative biliary stenting (yes vs. no), Gastrointestinal bleeding, Diarrhea at presentation, Inflammatory bowel disease, IBD, biliary stricture post- transplant, biliary infection |
| Hospital-related | Previous hospitalization, Same ward, Length of ICU stay >3 days, Hospitalization time, LOS, Hospitalization prior 2 mo, Hospital LOS prior to positive culture, Hospitalization inside 1 month before contagion, ICU admission, Hospital stay of longer than 14 days, Demographic model: Unit ICU, LOS >14 days prior to culture, Healthcare exposures previous 90 days (ICU admission), Transfer from outside hospital, Prolonged hospitalization (≥7 days), Hospital bed size, Hospital teaching status, Expected primary payer, Total prior Hospital Stay, Total Prior Admission in ICU, ICU stay within previous 30 days, Longitudinal model: Hospitalized (days), LOS prior to culture, Model 1 Risk for CDI: Lengh of stay before CDI, Model 2 outcomes linked to CDI : LOS, Lengt of hospitalization, Excessive bed rest, prior hospitalization during previous month, inpatient management, intensive care admission, admission days prior index culture, lenght of stay, hospital teaching status, hospitalization within 30 days prior to infection, intensive care unit admission, hospitalization, presence of preceding bacteremia during the hospitalization, admission days, hospital region, hospital location, in intensive care unit (ICU) at diagnosis, ICU at diagnosis, hospitalization in preceding 3 months, intensive care unit admission, LOS (model 1), previous hospital admission within 60 days, duration of hospital stay |
| Infection-related | Severe/fulminant CDI disease, S. maltophilia oral abundance, Previous bacteremia with another pathogen, Bacterial infection post- UCBT, Hospital-acquired pneumonia, Development of pneumonia within the first 10 days, Prior history of Clostridioides difficile, Healthcare-associated infection, Linezolid-resistant S. epidermidis, VRE colonization (within 30 days of HCT), Culture specimen–BAL fluid, Prior enterococcal infection, Polymicrobial infection, Previous S. maltophilia isolation, Breakthrough infection during carbapenem therapy, CMV reactivation, Herpesviridae reactivation other than CMV, culture from sputum specimens, Source of positive culture (Respiratory), Resistance model: History of PTZ-R, Outpatient CDI diagnosis., Type of Organism, Infection, Demographical and traditional model: Severe CDI, PCN exchange within 4 days of infection, CDI exposure, presence of BSBL-Resistant EnteroBacteria, ESBL-EB, and non E. coli EB in blood cultures, CRKP rectal colonization, Fever days, healthcare associated bloodstream infections (BSI), hospital acquired BSI, foci as healthcare associated pneumonia, gram negative bacteria coinfection, gram positive bacteria coinfection, septic shock, relapse after S. aureus bacteriemia., pathogene type, fungal infection within 30 days, index culture >48h, infection, GNB infection previous 3 months and total parenteral nutrition., >= 7 days colonized by carbapenem resistant K. pneumoniae, Candida spp Species, C. tropicalis, any bacterial pneumonia, other bacterial infection, solation of VRE in the prior 1 year, isolation of ESBL in the prior 1 year, central line associated infection, E.coli, Klebsiella spp., Pseudomonas spp, S. maltophilia, Acinetobacter spp, days from chemotherapy to GNB bacteremia, hospital days to GNB bacteremia, P. aeruginosa infection, Pitt bacteremia score, multi drug resistant bacteria culture-positive surveillance rectal swabs, abdominal infection, pulmonary infection, catheter-related infection, unknown origin infection, ESBL |
| Laboratory findings (non-microbiological) | Histology, Admit serum albumin, albumin, Preoperative hemoglobin, Albumin (<35 vs >35), FBG (>7 vs <7), Trigylceride, Total cholesterol, HDL, Hemoglobin, OB, Hypoproteinemia, Procalcitonin, C Reactive Protein, Serum Amyloid, polymorphonuclear leukocytes < 500/mmc for at least 10 days |
| Neutropenia | Severe neutropenia (<100/mm3), Febrile neutropaenia, Duration of neutropenia, Length of neutropenia ≥3 days, Severe neutropenia, Severe neutropenia, Typhilitis, ANC less than 110 cells/mm3, Profound neutropenia, Neutropenia, neutropenic days, Neutropenia, Haematological risk factors (neutropenia (days), Death during neutropenia, Severe neutropenia, neutropenia, absolute neutrophil count (ANC)less than 100 cells/mm3, neutropenia at any time, any bacterial pneumonia and neutropenia at any time., neutropenia, neutropenia, neutropenia, neutropenia |
| Other | Preceding fallout inside 1 month, Stem cell amount ≥2.5 * (vs. <2.5), Mucositis, [Indeterminable], mucositis,  Pancreatic texture (soft vs hard), denture, objective dry mouth scores, NACS (?), Level 2 of patient’s I tensity of Treatment Rating Scale, IMV., Indeterminable, performance status, complications (several), skin lesion, dissemination, treatment. 30 days, sepsis, recent endoscopic procedures, others" |
| Other treatments/medications | NSAID use, PPI use, Radiation, Proton pump inhibitors, Preoperative antacid use, Significant use of corticosteroids, proton pump inhibitor, Corticosteroid use, PP inhibitor use, Receiving glucocorticoids at BSI onset, Hemodialysis in previous 30 day, radiotherapy, previous radiation within 30 days, corticosteroid, use of steroids |
| Respiratory | COPD, Respiratory failure, Prior Mechanical Ventilation |
| Surgery-related | Surgical treatment, Portal vein embolization, Operation, Type of operation (pancreatoduodenectomy vs. others), Operation time, Estimated blood loss, Gastro-intestinal/abdominal surgery, Previous surgery inside 1 month, Invasive devices at the time of culture, Previous surgery, Invasive operation, gastro intestinal surgery, previous surgery within 30 days, previous surgery last 3 months, abdominal surgery, abdominal surgery, surgery in preceding 3 months, invasive procedure, surgery previous month |
| Transplantation-related | Organ transplantation, Prior HSCT, ASCT at relapse (vs. first-line treatment), History of HSCT, Stem cell transplantation, Autologous transplant, Allogeneic transplant, level 3 with HCST, level 3 without HCST and the HCST status, hematologic stem cell transplant, GVHD, living donor transplant, cytomegalovirus donor seropositivity, hepatic artery thrombosis post- transplant, intra-abdominal fluid collection post-transplant, number of re- operations in 1st month post- transplant., history of hemotologic stem cell transplantation, allogeneic matched unrelated transplant, hematologic stem cell transplantation |
| Urinary | Urinary catheter, Diversion type, Urinary catheter at culture, Prior Urinary Catheterization, urinary tract infection |
| Intravascular access | Blood transfusion, Presence of CVC, Dominant intravenous tube use, Presence of a CVC, Peripheral or Central CVP Line, central venous catheter (CVC), CVC removal, days from infection to CVC removal, presence of central venous catheter long term during index culture, GNB infection previous 3 months and total parenteral nutrition., central venous catheter use, parenteral nutrition, central venous catheter (CVC) related candidemia, Intravenous group, parenteral nutrition |
|  |  |
| **Mortality** |  |
| **Category** | **Included risk factors** |
| Antibiotic use | received vancomycin within 4 weeks prior to bacteraemia, meropenem exposition (yes/no), appropriate empirical antibiotic treatment, inadequate empirical antibiotic therapy, inadequate initial antibiotic treatment, imipenem empirical antibiotic regimen, bacterial growth during antibiotic treatment, administration of appropriate antibiotic treatment for minimum 72 hours, Use of antibiotics (≥3 agents), appropriate treatment/Non-appropriate treatment, Antimicrobial prophylaxis, initial antimicrobial failure, echinocandin pre-exposure, appropriate treatment within 48 h after blood culture collection, 3rd and 4th generation cephalosporines, fluoroquinolones., received appropriate therapy for XDR-ABC bacteraemia, inappropriate antibiotic therapy for multi drug resistant strains, inappropriate antibiotic therapy for multi drug resistant P. aeruginosa, Appropriate treatment vs Innappropiate treatment, previous antibiotics <3, early empirical TMP/SMX antibiotic use within 72 h, exposure to empirical antibiotics, azole monotherapy, Empirical antibiotic combination therapy, Inappropriate empirical antibiotic therapy, antibiotic treatmentfor the last 3 months before admission, appropriate antibiotic, empirical antifungal, antibiotiv prophylaxis, carbapenem empirical therapy, vancomycin empirical therapy, Model 3: guided antibiotics, duration of guided antibiotics, Model 4: empirical antibiotics, guided antibiotics, guided antibiotics, Model 6: duration of empirical antibiotics, Model 7: guided antibiotics, effective empirical antibiotics, inappropriate empirical antibiotic treatment, ultrasonography-driven necrotising enterocolitis NEC therapy with antibiotic regimens including tigecycline., adequate combined treatment within 48 hours, empirically, antibiotic prophylaxis, Appropriate empirical antibiotics within 48 h, 72-h IIAT, prior antimicrobial exposure, inadequate antibiotic treatment, inadequate initial antimicrobial therapy, received antibiotherapy with Sulfamethoxazole / trimethoprim, received appropriate treatment for S. maltophilia, timing of appropriate antibiotherapy, anti-staph treatment < 48 h, cefepime treatment, appropriate empirical antimicrobial therapy, inappropriate treatment, delay for appropriate treatment, antibiotic lock therapy, initial adequate therapy, inappropriate empiric antibiotic therapy., prior antifungal use, inappropriate empiric antibiotics, empirical treatment (No active drug, inappropriate empirical therapy, appropriate definitive therapy |
| Basic characteristics | Paediatric (Compared to adults), sex, age, Age years, Age >60, Age > 65 years, Age>45, Age, male gender, Age <60, Age, Ethnicity, Older age (>65 years old), age, sex, Age, sex, Age <60 years vs. Age >60 years, Clinical model: Demographic data (age), Smoking history (Never smoker/Former smoker/Current smoker), Age, Age, sex, Haematological malignancies: age, sex, Solid tumours: age, sex, Early case fatality: age, sex, Overall case fatality: age, sex, Age, gender, sex, age, Age (>60 vs. <60), year of diagnosis, age, Age group, sex, Sex, age, Sex, age, age >55 yr, Sex, age > 65 years, age, male gender, age at diagnosis, Age > 60 years, Age >60, sex, Age>55 years old, Age group, Model 2: age <65 years, 14 days mortality models 1 and 2 : age >65 years old, 30 days mortality models 1 and 2 : sex, age >65 years old, age, sex, Age, gender, age <65, gender, race, sex, age less than 18 years, Age, age 65 and over, age, Age, race, gender, age, sex, sex, age, age>55, sex |
| Cancer-related | type of malignancies, lung cancer, prostate cancer, advance neoplasm, adverse genetic group acute myeloid leukemia, Complete remission, relapsed/refractory disease, metastatic cancer, solid tumor/Hematologic malignancy, recent diagnosis (vs relapse), Performance status >2, site (respiratory vs other), fatal prognosis according to McCabe index, community site of acquisition, BCLC stage C or D vs. A or B, ALBI grade 3 vs. grade 1 or 2, Solid tumor vs Hematological, Recent diagnosis or remission vs Progression/re-lapse, Existence of distant metastasis, Primary sites of infection (Respiratory tract/Urinary tract/BSI), Underlying leukemia, leukemia, lymphoma, refractory or recurrent disease, primary disease acute myeloid leukemia, remission, Myelodysplastic syndrom, advanced neoplasm, advanced neoplasm, hematological malignancies, hematological malignancies, Nephroblastoma, neuroblastoma, sarcomas, Performance status score, tumor lysis, acute myeloid leukemia, acute myeloid leukemia, 14-day mortality model: solid tumor, cytology Poor vs. Favorable, cytology Poor vs. Intermediate, cytology Poor vs. Unknown, AML/ALL intermediate, AML/ALL/MDS advanced, MDS advanced, Karnofsky performance scale <90, Hodgkin’s lymphoma, Myelodysplastic syndrome, Solid neoplasia, haematological malignancy (Compared to solid tumours), solid tumor, malignancy-related complications, pancreatic tumour, biliary tumour, malignancy-related complications, underlying malignancies, disease relapsed or uncontrolled, relapsed or uncontrolled malignancy, acute myeloid leukemia, non Hodgkin´s lymphoma, Hodgkin´s lymphoma, cancer with metastasis, hematologic malignancy, Hematological malignancies, acute leukemia, acute myeloid leukemia, performance status, haematological malignancy, recurrent Hilar cholangiocarcinoma , acute myeloid leukaemia not in remission, malignancy type, hematologic malignancy, complete remission, solid tumor, hematological cancer, metastasis, complete remission of acute leukemia |
| Chemotherapy/immunosuppressants | type of the last chemotherapy treatment, day 15 bone marrow blast clearance, no recent chemotherapy (vs recent), sorafenib treatment, Chemotherapy, intensive chemotherapy. Model 2: Initial active treatment, intensive chemotherapy., High-dose cytarabine-containing chemotherapy, standard dose cytarabine chemotherapy, receiving chemotherapy, intensive Chemotherapy., previous chemotherapy within 30 days, induction or re-induction chemotherapy, chemotherapy |
| Comorbidities/clinical condition | APACHE-II score at onset of bacteraemia, Dependent functional capacity at baseline, pulmonary disease, liver disease, hæmotologiv stem cell transplant comorbidities index, renal failure, functional capacity at bacteraemia onset, comorbidities, type II diabetes mellitus, cerebrovascular disease, congestive heart failure, APACHE II, SAPS II score >40, SOFA score <10/>10, chronic kidney disease, multiorgan failure, end organ disease, APACHE II score, acute kidney infection or acute renal failure, diabetes Mellitus, SOFA score, SOFA score at diagnosis, SOFA score at healthcare associated infection (HAI) diagnosis, acute kidney injury within 30 days after HAI diagnosis, diabetes mellitus, chronic liver disease, and comorbidities., underlying cirhhosis, Child–Pugh class C vs. A or B, ECOG performance status, Comorbidities (Liver disease), Charlson comorbidity index CCI (1–2), CCI≥3, Intrapleural/abdominal infusion (within 30 days), Signs of severity, qSOFA score ≥2, APACHE IIscore≥19, hypotension (<90/50), CRI non hemorragic, Charlson comorbiditiy index I>3, renal Replacement Therapy, initial SOFA score, acute kidney injury, APACHE II score, Charlson Comorbidity index>3, Charlson scoring system, Charlson scoring system, comorbidities, chronic obstructive pulmonary disease, diabetes, hypertension, ECOG score binary, Charlson comorbidities index (CCI), number of organ dysfonction, chronic liver disease, HIV-infection. Adjusted for: chronic liver disease, diabetes mellitus, chronic renal failure, Charlson index, ECOG, Charlson index > 4 points, SOFA score, renal replacement therapy, SOFA score, ECOG 1, ECOG 2, ECOG 3, ECOG 4, diabetes mellitus, renal insufficiency, hematological disease newly diagnosed / relapsed after one remission, altered state of consciousness, acute renal failure, acute hepatic failure, comorbidities, qSOFA score, APACHE II score >20, APACHE II score >20, organ failure, charlson comorbidity index (CCI) >4, underlying disease., complications (hypoxia, SOFA score, Charlson comorbidity index, Charlson index score, the presence of complications or comorbidities (eg, hypertension, diabetes, Charlson score, cerebrovascular disease, dementia, Charleston Co-Morbidity Index, altered consciousness, acute renal failure, acute hepatic failure, hypertension, chronic heart disease, diabetes mellitus, organ failure and sepsis shock |
| Gastrointestinal | gastrointestinal symptoms |
| Hospital-related | location in hospital at onset of bacteraemia, inpatient, length of hospital stay in days before onset of bacteraemia, admission-days before bacteraemia, Hospital lenght of stay, Numbers of co-infected locations, hospital stay >30 days, Ontensive care unit stay, intensive care unit admission for healthcare associated infection (HAI), admission status, Length of hospitalization (≥21.0), intensive care unit admission, hospital stay, intensive care unit stay, previous hospitalisation, intensive care unit, intensive care unit admission, previous hospital admission, intensive care unit entrance, Length of hospital stay, days of hospitalization, intensive care unit requirement., duration of stay in hospital, ICU admission, duration of hospitalization prior to bacteremia, intensive care unit stay, ICU stay, intensive care unit stay, lenght of stay, intensive care unit residence, ICU admission, teaching status sof hospital, and the performance of bone marrow vs stem cell transplant during hospitalization., hospitalisation within 30 days prior to infection, admission to intensive care unit, Hospitalization, intensive care unit, presence of preceding bacteremia during the hospitalization, admission days, hospital region, hospital location, ICU at diagnosis, intensive care unit admission, LOS, ICU, duration of hospital stay. |
| Infection-related | shock at onset of bacteraemia), Clostridium difficile status (no infection/asymptomatic/symptomatic), type of bacteria, polymicrobial bacteraemia, CRGNB, septic shock, bacteremia origin (healthcare/nosocomial), clinical manifestations (Low grade fever (<38 °C/Shock at presentation), colonization with carbapenem resistant enterococci, gram-negative bacterial infection, infection with coagulase negative Staphylococci, infection with Acinetobacter baumannii, infection with Klebsiella spp., infection with extended spectrum beta lactamase-producing bacteria, infection with Gram-negative multi drug resistant bacteria, catheterization focus of infection, isolation of Gram + bacteria in culture, isolation of Gram - bacteria in culture, extended spectrum beta lactamase Enterobacteriaceae, carbapenem resistance, septic shock, multi-resistant Gram-negative BSI, pneumonia, Hemograms of bloodstream infections (white blood cell, Pathogens for bloodstream infections (among them 11 bacterias, fungi, polymicrobial and other), gram negative organisms, gram positive organisms, polyorganisms, and fungi, positive culture of multi drug resistant organism, Source of infection (Pneumonia), polymicrobial bloodtream infection, shock, colonized/Infected, septic shock, concurrent bacterial sepsis, albicans vs non albicans, Biofilm (strong vs weak), colistine resistance, fungal infection, phenotype, multi drug resistant (MDR) vs non-MDR bloodstream infection (BSI), P. aeruginosa vs other BSI, strong vs weak biofilm-producer, septic shock, infection related mortality, septic shock rate, multi drug resistant-polymicrobial bloodstream infections mortality, septic shock, fever, catheter-related candidaemia, mixed bloodstream infection, caspofungin resistance, polymicrobial bacteriemia, vaccine serotype VPV23, septic shock at diagnosis of XDR-ABC bacteraemia (extended drug resistant), days from 1rst positive culture to initial treatment, concomitant infection, bloodstream infection, received effective therapy for Klebsiella producing carbapenemase-Kp infection, catheter as a source of bacteriemia, pulmonary source of infection, intra abdominal source of infection, cONs, E.coli, P.aeruginosa, shock at onset, C. difficile infection, multi drug resistant organism-colonization, Gram-positive vs Gram-negative, Monomycrobial vs Polymicrobial, Non-multidrug resistant (MDR) vs MDR and/or extended spectrum beta lactamase, Gram negative bacteria, E.coli, K. pneumoniae, Enterobacter spp, Fungi, C. albicans. Candida spp, enterococcus, Septic shock, documented clinical source, breakthrough fungemia, prior vancomycin resistant enterococcus bacteremia (VREB), persistent VREB >48 hours, CoNS (coagulase negative staphylococci). Mortality 7 days model : coNS, polymicrobial infection, previous isolation of S. maltophilia, Focus of infection catheter related infection (CRI), focus of infection hemorragic, previous colonization, coinfection, >15 days VRE, MASCC score <21, MASCC score <21, coagulase-negative staphylococci, multi drug resistant gram negative bacteria, septic shock., septic shock at onset, pneumonia, Exacerbation of interstitial pneumonia, interstitial pneumonia, microbiology with or without non-fermenting Gram(-) bacilli, extended spectrum beta lactamase enterobacteria (ESBL-E), multiple bacterial infections, bacteria/fungal coinfections, colonisation by Klebsiella producing carbapenemase, colonisation by Acinetobacter, colonisation by Pseudomonas, pre-chemo infection, shock, shock, catheter realted infection, extended spectrum beta lactamase, catheter related infection, PITT bacteriemia score, VRE bactermia, other bloodstream infections, Vancomycin resistant enterococcus bloodstream infection, Pulmonary source, Abdominal source, Shock, Candidemia, HIV, catheter-related source, coagulase-negative staphylococci bacteremia, S. pneumoniae bacteremia, primary source of infection, catheter related source of infection, septic shock, C. tropicalis, Candida albicans (Compared to non-C.albicans or mixed), bacteraemia, shock, receiving Amphotericin B (Compared to those not receiving), after positive culture, MASCC risk score< 21, persistent bacteremia, septic Shock, fever >38C, multi drug resistant organism colonization (MDRO), multidrug resistant gram negative bloodstream infections (BSI), nonfermenter BSI, carbapenem resistant gram negative bacteria (CR - GNB), gram negative bacteria, pulmonary aspergillosis, ventilated at infection onset, Multi drug resistant organism colonisation (MDRO), Pitt score ≥ 4, MASCC score<21, carbapenem resistant gram negative bacteria , non fermentative bacteria, septic shock, carbapenem resistant by Klebsiella pneumoniae, type of bacteremia, caspofungin non-susceptibility, multidrug resistance, caspofungin non-susceptibility, multidrug resistance, Bacterial multi drug resistant infection, coexistence of fungal infection, clinically documented infection, Abdominal infection, unknown origin of infection, septick chock, extended spectrum beta lactamase carriage, carbapenem resistant enterobacteriaceae carriage, abdominal source, Meticillin-resistant Staphylococcus aureus (MRSA), infectious endocarditis, Pitt bacteremia score, extended spectrum beta lactamase-positive bacteremia, Pitt bacteremia score ≥4, C. difficile infection., septic shock etc.)., A.baumanii, polymicrobial infection, pneumonia, breakthrough bacteremia occurrence within 48 h of ongoing antibiotics, multi drug resistant E.coli, Candida spp Species, C. parapsilosis, abdominal infection, Bloodstream infection, C. difficile infection, isolation of vancomycin resistant enterobacteriaceae (VRE)in the prior 1 year, isolation of extended spectrum beta lactamase (ESBL) in the prior 1 year, isolation of CRAB in the prior 1 year, history of GNB bacteremia in the prior 1 year, pneumonia, Pitt score, septic shock, carba-non sensitive, days from chemotherapy to GNB bacteremia, hospital days to GNB bacteremia., sepsis, severity of sepsis (Sepsis, Severe sepsis or septic shock), P. aeruginosa infection, Pitt bacteremia score, septic shock, 3rd generation cephalosporins resistance by E. coli isolate., ESBL, perianal infection, pulmonary infection, CR-PA, MDR-PA |
| Laboratory findings (non-microbiological) | disseminated Intravascular Coagulation (DIC) score, systolic blood pressure , diastolic blood pressure, Hemoglobin, platelet), Serum albumin, Serum albumin, Alpha-fetoprotein > 400 ng/ml, Laboratory examination results (Hemoglobin g/L <110.0, Platelet count ×109/L <100.0, Lymphocytes count ×109/L <1.0, PCT ng/mL ≥1.0, Albumin g/L <30.0), hypoalbuminemia, T cell depletion, platelet count, median albumin level, median Albumin level, histology with or without squamous cells, Albumin to Globulin Ratio, C reactive protein, prothrombine activity, D-Dimer, creatinin, albumin, alkaline phosphatase, total bilirubine, blood lactate level (mmol/L), creatinine, Human leucocyte antigen 7/8, C reactive protein (CRP), Model 2: creatinine, CRP, Model 5: CRP, platelet count, creatinine level, histology: squamos cell carcinoma, histology: others, platelet < 10 × 103 mm−3, hemoglobin <70g/Dl, platelet <10×103mm−3, albumin <30g/L, AST >120U/L, total bilirubin >34.2μmol/L, prothrombin time >14s, glucose > 140 mg/dL, Nadir WBC count |
| Neutropenia | prolonged neutropenia prior to infection, Absolute neutrophil count, duration of neutropenia (days), Duration of neutropenia before blood culture, Neutropenia at the onset of bloodstream infection, severe neutropenia at the onset of bloodstream infection (<100/mm3), duration of neutropenia >21 days, neutrophil recovery, monocytopenia, neutropenia past month, current neutropenia, severe neutropenia past month, current severe neutropenia, febrile neutropenia, prolonged neutropenia, Neutrophils >500 vs Neutrophils 500, persistent neutropenia, neutropenia, 5x109/L neutrophil count while vancomycine resistant enterococcus (VRE) +, absolute monocyte count<100, monocytic acute myeloid leukemia, monocytes, 30-day mortality model: Neutropenia, Model 1: neutropenia, absolute neutrophile count 500 or more, ANC < 500/μl duration until BSI (days), neutrophil count, absolute neutrophil count, ANC<100/mmc, ANC<500/mmc, neutropenia, duration of neutropenia before infection, ANC less than 100 cells/mm³, neutropenia at any time., neutropenia, duration of neutropenia before BSI |
| Other | others(OPD/ ER), disease risk (favourable/intermediate/high), nasogastric tube, Disease status, combination therapy, mucositis, mucositis, < 0, DM, , nasogastric tube, etiology (secondary vs. de novo), white blood cell at diagnosis, Time period, prior therapies >2, Initial low tidal volume ventilation, status of disease, prone positionning treatment, extensive diagnosis, Indeterminable, type of CT last 3 months, palliative care, hemodynamic instability, adherence to protocol, etc.), skin lesion, dissemination, mean (± SD), At least one active drug), definitive treatment (One active drug, More than one active drugs)., BF |
| Other treatments/medications | current corticosteroid therapy, steroid administration, corticosteroids, steroid exposure, corticosteroid therapy, corticosteroid therapy, corticosteroid therapy, corticosteroid therapy., administration of steroid, radiotherapy dose in Gy, aciclovir, anthracycline, duration of vasopressors, hemodialysis, hemodialysis, corticosteroid use, receiving steroids, corticosteroid therapy, corticosteroid therapy, vasopressors treatment, use of vasopressors, vasopressors, vasopressors, previous radiation within 30 days, corticosteroid |
| Respiratory | Co-infections (respiratory), mechanical ventilation, positive culture in blood/respiratory tract/urinary tract/skin and soft tissue, respiratory symptoms, mechanical ventilation, mechanical ventilation, chronic lung disease, intubation, mechanical ventilation, bronchial stump fistula, mechanical ventilation, mechanical ventilation, respiratory source, severe acute respiratory distress syndrome, acute respiratory failure, acute respiratory failure, acute respiratory failure, mechanical ventilation, mechanical ventilation, mechanical ventilation, avute respiratory failure, mechanical ventilation |
| Surgery-related | emergent surgery, removal of invasive devices within the first 48 h, removal of invasive device or control deep infection, resection as hepatocellular carcinoma treatment, Surgery, presence of Drains postoperation; Infection-related model: Sample type (Urine/Blood culture), invasive procedures, invasive procedures, previous surgery within 30 days, invasive procedure, surgery |
| Transplantation-related | donor type (related/unrelated), human Leukocyte Antigen compatibility (match/mismatch), stem cell transplantation as consolidation therapy, prior hæmatologic stem cell transplant, Mortality 30 days model : Auto hematologic stem cell transplant treatment, Cord blood allograft, cytomegalovirus donor or receptor positive, hematologic cell transplant during 2010-2012, hematologic stem cell transplant HSCT, autologous HSCT, Post-transplant C. difficile infection, history of hemotologic stem cell transplantation, autologous transplant |
| Urinary | urinary catheter, source of bacteraemia (Urinary tract), urinary tract infection, Presence of indwelling urinary catheters, Model 1: Klebsiella producing carbapenemase-K. pneumoniae (KPC-KP) bloodstream infection (BSI) developing during inactive antibiotic treatment, KPC-KP BSI developing during inactive antibiotic treatment, foley catheter, urinary catheter at infection onset, urine tube, Urinary tract infection |
| Intravascular access | central catheterization, central venous catheter-related bloodstream infection (BSI), central venous catheter removal, central venous catheter removal within 48 h after blood culture collection, central venous catheter, central venous catheter removal within 48 hours, receiving total parenteral nutrition, blood transfusion, Model 1: central venous catheter removal, previous blood transfusion, catheter removal, CVC duration, CVC related candidemia, removal of CVC., Intravenous group, previous blood transfusion |
